# A graph-attentive GAN for rare-cell-aware single-cell RNA-seq data generation

**DOI:** 10.1371/journal.pcbi.1014601

**Published:** 2026-10-05

**Authors:** Ritwik Ganguly, Sana Aafrine, Sk Md Mosaddek Hossain, Sumanta Ray

**Affiliations:** 1 Computer Science and Engineering, Aliah University, Kolkata, India; 2 Computational Biology, Indraprastha Institute of Information Technology, New Delhi, India; 3 Data Science, The West Bengal National University of Juridical Sciences, Kolkata, India; The Chinese University of Hong Kong Faculty of Science, HONG KONG

## Abstract

A central challenge in downstream single-cell RNA sequencing (scRNA-seq) analysis is the high-dimensional, small-sample (HDSS) regime, often compounded by class imbalance from rare cell types. These factors hinder robust feature (gene) selection and cell clustering and limit the realism of samples generated by existing simulators. We introduce *GARAGE*, a **G**raph-**A**ttentive **RA**re-cell aware single-cell data **GE**neration that augments the generator’s input with a small, attention-weighted *‘leakage’* of real cells in addition to prior noise. Specifically, we build a *k*-nearest-neighbour cell graph and use a graph attention network (GAT) to prioritize nodes that likely represent under-sampled (rare) subpopulations; these high-attention cell embeddings are injected into the generator input to steer synthesis toward biologically plausible regions of the data manifold while respecting cell-type proportions. This attention-guided leakage accelerates training, reduces mode dropping, and yields realistic synthetic cells that preserve rare-cell structure. Across real scRNA-seq benchmarks, GARAGE improves downstream feature selection and clustering compared with state-of-the-art baselines. In summary, GARAGE directly addresses HDSS and rarity in scRNA-seq by coupling graph attention with adversarial generation to produce high-fidelity synthetic cells that enhance downstream analyses. The corresponding software is available at: https://github.com/RitwikGanguly/GARAGE.

## Author summary

Researchers frequently analyze single-cell transcriptomic data to unravel the mechanisms behind complex diseases. Yet, rare but important cell types are often too scarce in patient samples to study reliably. We introduce GARAGE - **G**raph-**A**ttentive **RA**re-cell aware single-cell data **GE**neration - a two-step framework that explicitly couples graph attention with adversarial learning. First, a neighborhood graph with attention weights surfaces “exemplar cells”, prioritizing rare populations. Second, we apply an attention-guided “leakage”: a small, curated set of these high-score real cells is injected into the GAN’s input alongside noise. This graph-attentive prior steers the generator to the full data manifold, accelerates and stabilizes GAN convergence, and prevents mode dropping, so rare cell types are synthesized faithfully. Across real scRNA-seq benchmarks, GARAGE produces more complete, biologically coherent datasets and consistently outperforms state-of-the-art methods in downstream feature selection and clustering. By tackling the high-dimensional, small-sample regime head-on, GARAGE delivers realistic synthetic cells that preserve rare-cell structure and diversity, providing a practical tool to power robust analyses and speed discovery.

## Introduction

The high-dimension, small-samples (HDSS) property of biological datasets, particularly single-cell RNA sequencing (scRNA-seq) data, creates a bottleneck in their interpretation. ScRNA-seq data are defined by each cell corresponding to its own gene expression, which involves thousands of genes, while the number of observations per cell remain limited to a few hundred. Such a high feature-to-sample ratio hinders statistical inference and predictive modeling and eventually leads to a decline in the stability of the analytical results (Vallejos et al. [1] and Ray et al. [2]). The challenge effectively introduces class imbalance in the dataset when the rare cell populations are concerned, which only amounts to a few sample representation. Although large-scale initiatives such as the Human Cell Atlas (HCA; Regav et al. [3]) and Mouse Cell Atlas (MCA; Han et al. [4]) enable significantly comprehensive representation of cellular populations across tissue, the ostracization of rare cells still lingers owing to their low abundance counts as compared to other dominant populations. The improper observation may eventually amount to low quality of synthesized samples and ruin the robustness of the conclusions drawn from downstream analyses (Button et al. [5]). Factors like ethical considerations, budget constraints, and limited patient materials might also be contributing to the dwindled availabilibilty of samples (Satija et al. [6]). At the same time, biological variation and technical artifacts—including amplification bias, dropout events, and batch effects—present another variability that must be kept in check during the experiments (Kiselev et al. [7]).

The analysis using scRNA-seq expression incorporates a number of steps: normalization, quality filtering, feature selection, clustering of expressions, and annotation of genes based on known marker profiles. Each step affect the consequent steps and play an important role in achieving satisfactory results. In particular, feature selection pose a significant step, directly impacting the heterogeneity of the expression space used in clustering of genes. Introducing predominantly noisy or irrelevant genes can obscure the distinction between expressional profiles, whereas an informative feature set inspire better cell separation and reduce the adverse effects of noisy and redundant data. Previous studies by Yang et al. [8] and Su et al. [9] highlight the role of a well-chosen subset of genes in improving the identification of distinct cellular populations and facilitating subsequent analyses. Gene selection is commonly approached through highly variable gene detection or differential expression testing, but both strategies falter in small-sample settings. When we are presented with a sparse dataset, even a few outliers can skew the variability estimates enough to reshape the resulting feature set (Liao et al. [10]). This is a real concern for scRNA-seq data, where thousands of genes are profiled at once but individual cell populations may be represented by only a small number of cells. The result is often an unstable feature subset, compounded by the computational burden of working in such high-dimensional space — a burden that grows further when stability must be assessed through repeated sampling or more complex modeling (Lu et al. [11]).

Since scRNA-seq datasets are so often sparse, researchers have increasingly turned to generative models and data augmentation to supplement what limited information is available. Goodfellow et al.’s introduction of generative adversarial networks (GANs) [12] was a key step in this direction. GANs consist of two competing components—a generator that produces synthetic samples and a discriminator that distinguishes generated samples from real data—and are trained jointly to approximate the underlying data distribution. Building on the original GAN framework, several variants have subsequently been proposed to address its limitations. Among these, the Wasserstein GAN (Arjovsky et al. [13]) improved training stability by employing the Wasserstein distance, while the *f*-GAN framework (Nowozin et al. [14]) provided a more general formulation based on *f*-divergences. Variational approaches (VAE) (Kingma et al. [15]) such as autoencoders have also been explored for simulating gene expression data (Zappia et al. [16]). In the context of scRNA-seq, several tailored generative methods have been proposed to augment small datasets with synthetic cells. For example, Lall et al. [17] developed LSH-GAN, which uses locality-sensitive hashing to guide the GAN training, producing realistic single-cell gene expression profiles from limited input samples. These efforts demonstrate that *in silico* generation of cells can, in principle, alleviate the HDSS problem by providing additional examples for downstream analysis.

Although several GAN variants have introduced conditioning or data-guided mechanisms, the form of guidance used in GARAGE is conceptually different. In a conditional GAN (cGAN), class labels or external covariates are supplied to the generator and discriminator so that generation is explicitly controlled by known labels. Similarly, Auxiliary Classifier GAN (AC-GAN) augments the discriminator with an additional class-prediction objective, thereby encouraging class-discriminative synthetic samples. These approaches are useful when reliable class labels are available and when the main objective is label-conditioned generation. However, they do not directly address the problem of rare-cell under-representation in high-dimensional small-sample scRNA-seq data, where minority populations may be poorly sampled and may not provide sufficient class-level information for stable conditional generation. LSH-GAN, in contrast, uses locality-sensitive hashing to guide GAN training from limited single-cell samples, but it does not learn graph-attention scores over the cell–cell neighbourhood structure to prioritize biologically informative or rare-cell-enriched seed cells. GARAGE differs from these approaches by constructing a *k*-nearest-neighbour cell graph, learning attention coefficients through a graph attention network, and using the resulting high-priority cells as leakage-controlled anchors in the generator input. Thus, the proposed attention-guided leakage mechanism is not a standard conditioning vector or an auxiliary classification loss, rather, it defines a graph-informed hybrid input distribution that combines stochastic noise with attention-selected real-cell embeddings to steer synthesis toward rare and biologically meaningful regions of the single-cell manifold. A detailed architectural comparison of GARAGE with standard GAN, cGAN, AC-GAN, WGAN/f-GAN, and LSH-GAN is provided in the subsection “Comparative positioning of GARAGE with related GAN variants” and Supplementary Table (1) in S1 File.

However, a notable limitation of existing simulators is their treatment of rare cell types. Most generative models aim to capture the overall distribution of the data, implicitly emphasizing common cell states at the expense of rare ones. As a result, minor subpopulations often remain underrepresented or entirely absent in the synthetic data. The class imbalance present in the original data is usually carried over into the generated samples. In practice, this means that a generative model might faithfully reproduce abundant cell types but fail to generate enough examples of a rare but important cell type (for instance, a rare stem cell or a rare pathogenic cell state), thereby failing to correct the imbalance that plagues small-sample analyses. Without explicitly addressing this issue, the value of synthetic data for improving downstream tasks like feature selection and clustering is limited – rare cell types, which might be the ones most in need of augmentation, remain challenging to recover.

In this work, we addressed these challenges by introducing *GARAGE*, a **G**raph-**A**ttentive **RA**re-cell Aware single-cell data **GE**neration framework. GARAGE is a novel GAN-based approach that explicitly targets rare cell subpopulations during data generation. The core idea of GARAGE is an attention-guided “leakage” mechanism that feeds a small amount of informative real data into the generator along with the usual random noise. Specifically, GARAGE constructs a *k*-nearest neighbor (*k*NN) graph of cells from the original dataset following Dong et al. [18], with each cell represented as a node and relationships between neighboring cells encoded as graph edges. A graph attention network (GAT) subsequently operates on this graph to assign importance scores to individual cells, enabling the identification of cells associated with poorly represented populations and those exhibiting distinctive transcriptional characteristics. We then sampled a few cells with the highest attention weights and leaked their embeddings (gene expression vectors) into the GAN generator’s input, together with the standard noise vector. These attention-guided cells provide additional information to the generator, directing the generative process toward regions of the cellular expression space that are inadequately represented in the training data. Rather than generating samples solely from random latent inputs, the model therefore receives guidance from real cells, which helps preserve biologically meaningful expression patterns associated with rare populations. The use of real cellular information as guidance also contributes to more stable adversarial training, helping the generator learn representative expression patterns for sparsely sampled populations and reducing the tendency toward mode collapse.

The effectiveness of GARAGE was assessed using real scRNA-seq datasets, with experiments showing improved performance in subsequent downstream analyses. The synthetic cell samples generated by GARAGE more faithfully recapitulate the characteristics of the original data (especially for rare cell classes) compared to those from existing state-of-the-art methods. In particular, when using GARAGE-generated data to augment small single-cell datasets, we observed improvements in feature selection and cell clustering accuracy over baselines such as standard GANs and the LSH-GAN approach. These improvements highlight GARAGE’s ability to not only produce realistic data in terms of distribution matching but also to enhance the biological signal available for identifying key genes and delineating cell types. In summary, by coupling graph-based attention with adversarial generation, GARAGE effectively addresses the dual challenges of HDSS data and rare-cell imbalance in single-cell analysis.

Our main contributions are as follows:

(i) We present a novel generative framework (GARAGE) that directly tackles the difficulties of gene selection and cell clustering in high-dimensional, small-sample scRNA-seq data.
(ii) GARAGE introduces an attention-guided leakage mechanism in GAN training: a graph attention network identifies rare-cell candidates on a cell–cell graph, and a small subset of these real cells (with high attention scores) is injected into the generator input along with random noise.
(iii) By incorporating this graph-attentive prior, GARAGE achieves faster and more stable training than conventional GANs. The generator converges more rapidly and avoids dropping modes, ensuring that rare cell types are retained in the synthetic data.
(iv) Empirical results show that GARAGE outperforms state-of-the-art baselines (including standard GAN variants and LSH-GAN) in generating realistic single-cell gene expression data. The synthetic datasets produced by GARAGE maintain correct cell-type proportions and capture rare subpopulations more effectively.
(v) Using GARAGE-generated samples for downstream analysis leads to improved feature selection and cell clustering outcomes. In our experiments, gene selection algorithms and clustering methods applied to the augmented data identified biologically meaningful genes and cell groupings that were missed under baseline methods, confirming the practical advantage of our approach.

## Materials and methods

This section explains the proposed methodology involved in developing and testing our *GARAGE* framework. Thereafter, we present the theoretical foundations of the fundamental components, the graph attention network (GAT), and the generative adversarial network (GAN), prior to discussing their integration within our proposed two-stage GARAGE architecture. The section concludes with the formal mathematical derivation of our model, giving an extended description of its innovative generative process.

### Workflow

The proposed framework uses single-cell RNA sequencing (scRNA-seq) data as its real-data input. Each cell is represented by a vector of gene expression values, providing a high-dimensional description of its transcriptomic state. These profiles preserve the biological variation among individual cells and form the basis of the proposed graph-guided generative model. The overall architecture is illustrated in Fig 1, and its main components are described below.

- **Data preprocessing:** Raw scRNA-seq data are subjected to preprocessing before being provided to the learning framework. Normalization corrects for differences in sequencing depth and library size across cells, bringing expression values onto a comparable scale. Quality control is applied alongside this to filter out cells or genes with poor expression coverage or other problematic characteristics. Together, these steps yield a cleaner transcriptomic representation and limit the extent to which technical noise influences model training downstream.
- **Cell neighbourhood graph construction:** Once preprocessing is complete, a cell neighbourhood graph is built to capture local relationships among cells based on transcriptomic similarity. Cells are represented as nodes, and edges connect cells whose expression profiles resemble one another. We use a *k*-nearest neighbours (KNN) approach, following Dong et al. [18], to identify each cell’s *k* most similar counterparts. The resulting graph captures the local structure of the cell population, grouping transcriptionally similar cells into connected neighbourhoods.
- **Graph attention network (GAT):** The generated cell graph is subsequently processed by the graph attention network (GAT) (Velivckovic et al. [19]). GAT utilizes an attention mechanism that allows for unequal contributions of the neighboring nodes when aggregating information on graphs. In the proposed framework, the GAT model generates representations of individual cells by aggregating information from their neighboring cells while giving more weight to the more informative interactions. Thus, the generated node representations are able to contain both transcriptomic information of individual cells and their interactions with neighboring cells. The attention mechanism offers additional means for selecting informative cells and interactions for the future generation process.
- **Generator:** The generator takes as input the embeddings extracted using the GAT along with a noise vector and generates synthetic cell profiles using the aforementioned inputs. The embeddings help bring structural and biological relevance to the data, while the noise helps introduce variability in the synthetic samples generated and also makes them less dependent on the individual training data points. The generator learns, using adversarial training, how to generate gene expression profiles similar to those of the actual scRNA-seq data. The aim is to produce diverse synthetic cells with biological significance contained within the original dataset.
- **Discriminator:** The discriminator network learns to differentiate between real scRNA-seq profiles and those created by the generator. In each round of training, both real and synthetic expressions are fed to the discriminator network, which determines if a particular sample comes from the actual or generated data. The resultant discriminator loss is then used to update the weights of the network through the process of backpropagation. At the same time, the generator is trained using the feedback received from the discriminator in order to create more realistic samples. This iterative process helps in generating more realistic samples from the generator network. The final generator network can then be used for generating synthetic scRNA-seq profiles for applications such as data augmentation and downstream analysis.

**Fig 1:**
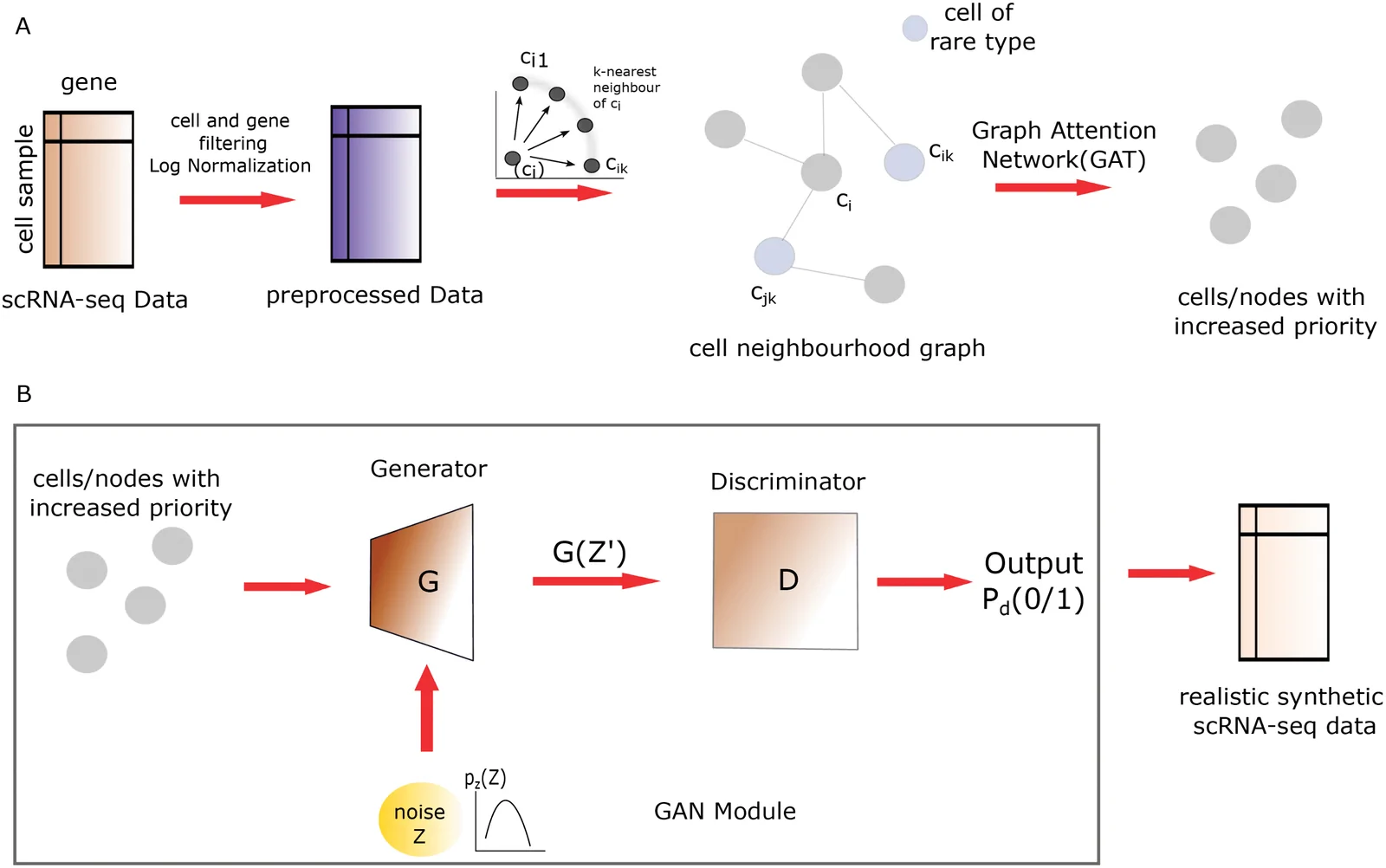
Workflow of the proposed GARAGE model. A: Preprocessed scRNA-seq data are transformed into a cell–cell neighbourhood graph. A graph attention network (GAT) assigns higher attention weights to rare or distinctive cells, and these prioritized cells are partially leaked into the generator input together with random noise. B: The GAN module (generator + discriminator) uses this graph-attentive input to produce realistic synthetic scRNA-seq data while preserving cell-type proportions and enhancing rare subpopulations.

### Underline theory that supports GARAGE

*GARAGE* model is a combination of two deep learning models: Graph Attention Network (GAT) introduced by Velivckovic et al. [19] and Generative Adversarial Network (GAN) introduced by Goodfellow et al. [12]. Each of them solves a different aspect of the problem of generating synthetic data in the two-stage *GARAGE* architecture. Specifically, GAT learns the relationships between cells and detects which cells or cellular neighborhoods contain more informative signal. The GAN then uses this information along with the stochastic information to learn the underlying distribution of the data and generate synthetic cells. Thus, GAT provides information based on the learned graph structure, and GAN is responsible for generating synthetic scRNA-seq data. In the following subsections, we describe the theory behind GATs and GANs and later discuss how they are implemented in the **GARAGE** architecture.

#### Graph attention network (GAT).

The GAT used for identifying informative cells in the *GARAGE* framework plays a vital role. The scRNA-seq data is first represented as a graph with the cells represented as nodes and edges between cells with similar transcriptomic profiles. In this way, we can consider the interactions between the cells in contrast to treating each cell as a separate observation. Graph Neural Networks (GNNs) are designed with the capacity to handle graph-structured data (Scarselli et al. [20]; McCulloch et al. [21]). The primary objective of the attention mechanism, first introduced in the field of computer vision (Guo et al. [22]), is to mitigate the computational cost of image processing by enhancing the performance of the image by bringing in a model that would target only certain parts of the image rather than the whole image.

A foundational GNN architecture is the graph convolutional network (GCN) (Kipf et al. [23]), which learns node representations by aggregating feature information from their local neighbourhoods. However, a key limitation of standard GCNs is their isotropic aggregation mechanism; they treat all neighbouring nodes with equal importance during the feature aggregation step. This can be suboptimal for scRNA-seq data, where certain cells within a neighbourhood may be more representative or influential. Thus, rather than focusing on the entire graph as a whole, we can focus on specific parts of the graph (nodes) and perform feature aggregation.

The graph attention network (GAT) directly overcomes this limitation by incorporating a self-attention mechanism into the graph convolutional operation. Instead of uniform aggregation, a GAT learns to assign a unique importance weight, or attention coefficient, to each neighbouring node when updating a target node’s representation. This allows the model to dynamically focus on the most relevant neighbours for a given task, leading to more powerful and expressive node embeddings.

In the *GARAGE* framework, we leverage this property of GATs in a novel way. We first train the GAT on a supervised task: classifying the cell-type labels of the nodes in the cell neighbourhood graph. Our central hypothesis is that the attention coefficients learned during this process serve as a direct proxy for a cell’s biological importance and representativeness. We mainly focused on the rare represented cell types and cells that the GAT consistently assigns high attention to, which are those that are most informative for defining the identity of their local neighbourhood. After training, we aggregate these learned attention scores for each cell to derive a global “importance score.” By ranking cells according to this score, we can select the top-k% of cells, which we term **“priority nodes**.” These GAT-selected cells form the high-fidelity seed set used to guide the generator in the subsequent adversarial training stage. Complete algorithmic details for the attention mechanism, including full theoretical derivations (attention mechanics, seed construction, convergence analysis, and GAT parameters), are provided in the Supplementary Note (3.1) in S1 File.

**Label requirement and possible weakly supervised extensions.** In the present implementation, cell-type labels are used to train the GAT module so that the learned attention coefficients prioritize cells that are informative for distinguishing known cell populations, including rare or under-represented classes. These labels are therefore used to construct a supervised attention prior and to support quantitative benchmarking through label-based metrics such as ARI, NMI, and macro-F1. However, the attention-guided leakage mechanism itself does not require labels once a cell-priority score has been obtained. In other words, GARAGE requires a ranked set of informative seed cells, but this ranking does not necessarily have to be derived from fully supervised cell-type annotation.

This distinction allows GARAGE to be extended to partially labelled, weakly supervised, or label-scarce settings. In a semi-supervised setting, the GAT module can be trained using a small labelled subset together with pseudo-labels, consistency regularization, or graph-based label propagation. In a weakly supervised setting, noisy labels can be generated from marker-gene rules or expert-defined marker signatures and then used to guide the attention module. Alternatively, when reliable labels are unavailable, the priority scores can be derived from unsupervised graph-based criteria such as local density, graph centrality, cluster uncertainty, neighborhood entropy, or outlier scores computed from an initial low-dimensional embedding. These variants preserve the central idea of GARAGE: a cell-priority mechanism first identifies informative or under-sampled regions of the single-cell manifold, and the resulting seed cells are then used to guide the generator through attention-guided leakage.

#### Generative adversarial network (GAN).

Our approach for generating high-fidelity single-cell RNA-seq data is built upon the generative adversarial network (GAN) framework, first proposed by Goodfellow et al. [12]. GANs are a class of deep generative models (Hinton et al. [24]) uniquely suited for learning complex, HDSS data distributions, making them a powerful tool for biological data synthesis. With the help of such an approach, we are able to create realistic synthetic samples, which will allow us to tackle the issues associated with research in the area of bioinformatics, such as the lack of data, expensive experiments, and the issue of patient anonymity.

The architecture of the GAN includes a zero-sum game of min-max between two neural networks: a Generator (*G*) and a Discriminator (*D*). The goal of the generator is to learn the distribution of the actual data. To do this, the generator uses a latent variable *z* and tries to transform it into some synthetic sample *G*(*z*) that cannot be differentiated from the real data. At the same time, the discriminator acts like a binary classifier and tries to distinguish the real data from the synthesized fake data. The two networks are trained in an iterative, adversarial manner: the generator tries to maximize the discriminator’s classification error, while the discriminator strives to minimize it. This competitive dynamic continues until an equilibrium is reached, at which point the generator produces samples whose distribution is very close to that of the real data.

From a statistical perspective, generative models (Harshvardhan et al. [25]) aim to learn the underlying probability distribution of a training dataset, denoted as *p*_data_(*x*), in order to draw new, realistic samples. Traditional approaches, such as the Hidden Markov Model (HMM) (Awad et al. [26]) or Gaussian Mixture Models (Viroli et al. [27]), are often explicit generative models. They define a parametric mathematical form for the probability density function (pdf) and learn its parameters, typically by maximizing the likelihood of the training data. However, for HDSS and complex data like single-cell transcriptomes, defining and optimizing such an explicit density function is often intractable.

GANs offer a fundamentally different and powerful alternative as an implicit generative model. Instead of defining a formula for the pdf, a GAN learns a procedure to sample directly from the target distribution. It achieves this by transforming a simple latent variable *z*, drawn from a prior distribution *p*_z_(*z*), through the complex, non-linear function incorporated by the generator network, G(z). This process induces a learned data distribution, *p*_g_. The statistical essence of the adversarial training lies in its mechanism for matching *p*_g_ to *p*_data_. The min-max game between the generator and discriminator functions as an effective approach to minimize a statistical divergence between the two distributions. In the original framework, it has been proven that the process is equivalent to the minimization of Jensen-Shannon (JS) divergence (Menendez et al. [28]). Such an indirect approach helps GANs capture all the statistical aspects of the data without having to calculate or even define its likelihood, hence making them extremely suitable for modeling the complicated, sparse, and multi-modal nature of scRNA-seq data. Formally speaking, the training of GAN is modeled after Goodfellow et al. [12]:


$$\begin{array}{c} \min_{G}\max_{D}V(D,G)={𝔼}_{x\sim p_{\text{data}}(x)}[\log D(x)]+{𝔼}_{z\sim p_{z}(z)}[\log(1-D(G(z)))] \end{array}$$ (1)

GANs provide several benefits over other generative algorithms such as Variational Autoencoders (VAEs) (Kingma et al. [15]), Restricted Boltzmann Machines (RBMs) (Fischer et al. [29]), Deep Belief Networks (DBNs) (Hinton et al. [30]), and denoising autoencoders (DAEs) (Vincent et al. [31]). These benefits become especially valuable when dealing with high-dimensional and sparse scRNA-seq data. The first one is the fact that the models learn the underlying distribution of the data implicitly. There is no need to explicitly define a probability density function as the generator approximates the distribution based on its adversarial interactions with the discriminator. This approach becomes especially useful for complicated and high-dimensional data sets where formulating a suitable probability model is problematic. In addition, the adversarial objective allows assessing the quality of generated samples based on the resemblance of their distribution to the true distribution. Finally, the generator allows generating samples directly using forward evaluation without iterations used in Markov chain-based methods. More details about GAN formulation are provided in the Subsection (3.2) in S1 File..

Despite these advantages, conventional GANs present several challenges, most notably training instability and mode collapse, in which the generator may produce samples concentrated around only a limited portion of the target distribution. These limitations are particularly important for scRNA-seq data, where rare cellular populations may occupy relatively small regions of the overall data distribution. To address this issue, we introduce **GARAGE**, an architectural extension that incorporates a graph attention network (GAT) into the generative framework. The GAT provides structure-aware information from the cellular neighbourhood graph and guides the generator toward informative regions of the data space. The proposed integration of graph-based attention with adversarial generation is described in the following section. The complete set of GAN parameters is provided in Subsection (3.2) in S1 File.

### The proposed GARAGE framework: A GAT-seeded generative model

The proposed *GARAGE* framework combines a graph attention network (GAT) and a generative adversarial network (GAN) within a two-stage architecture, as shown in Fig 1. The main motivation behind this design is to address the difficulty of training conventional GANs on high-dimensional, sparse single-cell data. Rather than allowing the generator to learn entirely from randomly sampled inputs, GARAGE first identifies cells that are informative from a biological and graph-structural perspective. These cells are subsequently used to guide the generation process, helping the model remain within a biologically meaningful region of the data space.

**The first stage** (Fig 1A) is concerned with finding the cells that are most useful as generation seeds. A neighbourhood graph is constructed from the preprocessed scRNA-seq data using the kNN graph approach [18]. This graph is subsequently used to train a GAT for cell-type classification. A notable characteristic of the GAT is that while aggregating information from neighbouring cells, it learns attention weights, which help to estimate the impact of individual cells on the learned representation. Attention weights allow identifying the cells that have the strongest impact on the representation through their attention values. After ranking cells by their aggregate attention scores, the top-k% are chosen as the seed set, which will be used further. The selected cells are representatives of the cellular states and will be sent to the generative phase.

**The second stage** (Fig 1B) represents the modification of the classic input for the generator of the GAN architecture, which is a novel aspect of the module design. In contrast to the conventional approach where the generator is provided only with a vector of random noise *z*, GARAGE uses the hybrid input $Z^{\prime }$, which is a combination of the seed cells and random noise vectors. Seed cells represent the reference states observed in the real data and random noise enables the generator to generate new synthetic states different from those provided by seed cells. Hybrid inputs are shuffled before being fed into the generator *G*, which produces the generated sample $G(z^{\prime })$.

The generator is trained by the adversarial method using the objective function defined by Eq. (1). The discriminator *D* receives real samples *x* from the complete dataset as well as synthetic samples generated by $G(z^{\prime })$, and learns to distinguish between the two. At the same time, the generator attempts to produce samples that are sufficiently realistic to make this distinction difficult for the discriminator. Here, $E_{x}$ denotes the expectation over the real data distribution, while $E_{z}$ represents the expectation associated with the hybrid input distribution $Z^{\prime }$ used by the proposed seeded generator. Through this iterative, adversarial training, the generator learns not only how to transform noise into coherent gene expression profiles but also how to produce plausible variations around the high-quality seed cells. This process continues until an equilibrium is established, at which point the discriminator can no longer reliably distinguish between the real data and the high-fidelity synthetic data generated by GARAGE.

### The mathematical formulation of GARAGE

As mentioned earlier, the *GARAGE* framework comprises two stages: (1) a GAT-based module that identifies a set of attention-prioritized, high-importance “seed” cells; and (2) a GAN whose generator is guided by these seeds through a batch-level hybrid input that mixes sampled GAT-prioritized seed cells with random noise vectors.

Let the preprocessed single-cell dataset be represented by a matrix $𝐗\in {\mathbb{R}}^{N\times M}$, where *N* is the number of cells and *M* is the number of genes.

**Stage 1: GAT-based representative cell selection**

• We model cellular relationships as a graph 𝒢=(𝒱,ℰ), where $𝒱=\{c_{1},...,c_{N}\}$ indexes cells and edges ℰ are built by a *k*-nearest-neighbor procedure in gene-expression space (KNN graph; Dong et al. [18]). Each node $c_{i}$ carries an input feature ${𝐱}_{i}\in {\mathbb{R}}^{M}$.

• A GAT produces attention coefficients $\alpha_{ij}$ per edge $(c_{i},c_{j})\in ℰ$. For a single head,


$$\begin{array}{c} \alpha_{ij}=\frac{\exp\;\left(LeakyReLU\;\left({𝐚}^{⊤}\left[𝐖{𝐡}_{i}\;\|\;𝐖{𝐡}_{j}\right]\right)\right)}{\sum_{k\in {𝒩}_{i}}\exp\;\left(LeakyReLU\;\left({𝐚}^{⊤}\left[𝐖{𝐡}_{i}\;\|\;𝐖{𝐡}_{k}\right]\right)\right)}, \end{array}$$ (2)

where ${𝐡}_{i}\in {\mathbb{R}}^{F}$ are node features (possibly a projection of ${𝐱}_{i}$), $𝐖\in {\mathbb{R}}^{F^{\prime }\times F}$ and $𝐚\in {\mathbb{R}}^{2F^{\prime }}$ are learnable, ‖ denotes concatenation, and ${𝒩}_{i}$ is the neighborhood of $c_{i}$.

• After training the GAT (e.g., for cell-type prediction), we define an importance score that aggregates incoming attention to node $c_{i}$:


$$\begin{array}{c} S(c_{i})\;=\;\sum_{j\in {𝒩}_{i}}\alpha_{ji}. \end{array}$$ (3)

Cells are ranked by $S(c_{i})$, and the top-*K* (or top-*q*%) form the set of representative *priority nodes*. We collect their raw or final-layer GAT embeddings into ${𝐗}_{\text{new}}\subset 𝐗$ and denote the index set by $S_{q}$.

**Stage 2: GAT-seeded adversarial generation**

The novelty of **GARAGE** lies in replacing the purely noise-driven generator input with a *batch-level hybrid input*. In the reported experiments, the GAT-selected priority cells were not collapsed into a single attention-pooled seed vector. Instead, the priority seed set ${𝐗}_{new}$ was used directly: at each GAN iteration, a fraction of the generator input batch was sampled from these GAT-prioritized seed cells, while the remaining fraction was sampled from the random noise prior. Thus, the leakage mechanism operates through batch-wise mixing of biologically informative real-cell anchors and stochastic noise inputs.

• *Conventional baseline:* A standard GAN solves the min–max game


$$\begin{array}{c} \min_{G}\max_{D}\;V(D,G)\;=\;{𝔼}_{𝐱\sim p_{\text{data}}}[\log D(𝐱)]\;+\;{𝔼}_{𝐳\sim p_{z}}[\log(1-D(G(𝐳)))], \end{array}$$ (4)

where *p*_data_ is the real data distribution and $p_{z}$ is a simple prior (e.g., 𝒩(0,I)).

• *Attention-ranked seed distribution:* After training the GAT, we aggregate the learned incoming attention coefficients to obtain an importance score for each cell:


$$\begin{array}{c} S(c_{i})=\sum_{j\in {𝒩}_{i}}\alpha_{ji}. \end{array}$$ (5)

Cells are ranked according to $S(c_{i})$, and the top-*K* cells form the GAT-prioritized seed set


$$\begin{array}{c} {𝐗}_{new}=\{{𝐱}_{i}|c_{i}\in TopK(S)\}. \end{array}$$ (6)

In the reported experiments, this discrete seed set was used directly. We did not collapse the selected cells into a single attention-pooled vector. Therefore, ${𝐗}_{new}$ should be interpreted as an empirical seed distribution from which high-attention real-cell anchors are sampled during GAN training.

• *Batch-level hybrid generator input used in the experiments:* For a generator input batch of size *m*, the leakage rate λ∈[0,1] determines how many GAT-selected seed cells are inserted into the batch:


$$\begin{array}{c} m_{seed}=\lfloor \lambda m\rfloor ,\;m_{noise}=m-m_{seed}. \end{array}$$ (7)

We sample $m_{seed}$ cells from ${𝐗}_{new}$ and $m_{noise}$ random noise vectors from $p_{z}(𝐳)$. The hybrid input batch is then


$$\begin{array}{c} {𝐙}^{\prime }=\{{𝐱}_{seed}^{\left(1\right)},...,{𝐱}_{seed}^{\left(m_{seed}\right)}\}\cup \{{𝐳}^{\left(1\right)},...,{𝐳}^{\left(m_{noise}\right)}\}. \end{array}$$ (8)

Here, ∪ denotes batch-wise concatenation followed by shuffling, not feature-wise concatenation into a single vector. This batch-level construction is the attention-guided leakage mechanism used in all reported experiments.

• *GARAGE objective:* Using the batch-level hybrid input distribution, the GARAGE adversarial objective is written as


$$\begin{array}{c} \min_{G}\max_{D}\;V_{GARAGE}(D,G)={𝔼}_{𝐱\sim p_{data}}[\log D(𝐱)]+{𝔼}_{{𝐳}^{\prime }\sim p_{{𝐙}^{\prime }}}[\log(1-D(G({𝐳}^{\prime })))]. \end{array}$$ (9)

Here, $p_{{𝐙}^{\prime }}$ denotes the empirical hybrid input law induced by the batch-wise mixture of GAT-selected seed cells and random noise vectors. When λ=0, ${𝐙}^{\prime }$ contains only random noise and GARAGE reduces to a conventional GAN. When λ>0, a controlled fraction of the generator input batch consists of attention-prioritized real-cell anchors, thereby guiding the generator toward biologically informative and rare-cell-relevant regions of the scRNA-seq manifold.

(i) The construction in Eqs. (7)–(8) clarifies that leakage is implemented at the batch level rather than by forming a single pooled seed vector.
(ii) The seed component of the batch is obtained from the GAT-prioritized empirical distribution ${𝐗}_{new}$, whereas the remaining component is sampled from the random noise prior $p_{z}(𝐳)$.
(iii) This description is consistent with Algorithm 1, which was the procedure used in all reported experiments.

For reproducibility, the architecture and training settings used in the reported experiments are summarized in Supplementary Tables (2) and (3) in S1 File. The GAT module was implemented as a two-layer GATConv network on a *k*-nearest-neighbour cell graph with *k* = 5. The first GAT layer used 8 attention heads with 32 hidden units per head, followed by a ReLU activation; the second GAT layer used a single attention head to produce the final cell-type prediction. The GAT was trained using cross-entropy loss and the Adam optimizer with learning rate 0.005 for 7501 epochs. The generator was implemented as an MLP with two hidden layers of 512 neurons each and LeakyReLU activation with negative slope 0.2, followed by a linear output layer. The discriminator was implemented as a smaller MLP with hidden layers of 256 and 128 neurons, also using LeakyReLU activation. The generator and discriminator learning rates were 0.0002 and 0.0004, respectively. GAN training was performed for 20001 iterations, with $n_{d}=5$ discriminator updates and $n_{g}=2$ generator updates per iteration. The hybrid input used in the reported experiments was the batch-level mixture defined in Eqs. (7)–(8), no attention-pooled seed vector or γ-controlled pooling was used in the reported results.

The detailed algorithm for the entire *GARAGE* framework can be found in the algorithm 1. The complete architecture and dataset-specific hyperparameters used in all reported experiments, including the GAT configuration, generator and discriminator layer sizes, optimizer settings, leakage rates, and batch sizes, are provided in Supplementary Tables (2) and (3) in S1 File.

**Algorithm 1. The GARAGE framework algorithm**.

**Require:** scRNA-seq data matrix $𝐗\in {\mathbb{R}}^{N\times M}$; cell-type labels $𝐲\in \{1,...,C{\}}^{N}$; number of nearest neighbours $k_{nn}=5$; GAT architecture: two GATConv layers, first layer with 8 heads and 32 hidden units per head, second layer with 1 head; GAT optimizer: Adam, learning rate 0.005, epochs $E_{GAT}=7501$; leakage percentage λ∈{0.10,0.20,0.30}; batch size *m*; GAN iterations $T_{GAN}=20001$; generator architecture [512,512] with LeakyReLU(0.2) and linear output; discriminator architecture [256,128] with LeakyReLU(0.2); generator learning rate 0.0002; discriminator learning rate 0.0004; discriminator/generator steps $n_{d}=5,n_{g}=2$.

**Ensure:** Trained generator network $G_{\theta_{g}}$.

**Stage 1: GAT-based representative cell selection**

1: Construct k-NN graph 𝒢=(𝒱,ℰ) from data 𝐗 using *k*_nn_.

2: Initialize GAT classifier $M_{GAT}$ with parameters ϕ.

3: **for** *epoch*=1 **to** $E_{GAT}$ **do**

4:  *Train* $M_{GAT}$ *on* 𝒢 *to predict labels* 𝐲 by minimizing cross-entropy loss.

5: **end for**

6: Extract final-layer attention coefficients $\left\{\alpha_{ij}\right\}$ from trained $M_{GAT}$.

7: **for** each cell $c_{i}\in 𝒱$ **do**

8:  Calculate importance score:

$S(c_{i})\leftarrow \sum_{j\in {𝒩}_{i}}\alpha_{ji}.$

9: **end for**

10: Select the set of top-*K* seed cells:

${𝐗}_{\text{new}}\leftarrow \{{𝐱}_{i}|c_{i}\text{ is in top }K\text{ based on }S(c_{i})\}.$

**Stage 2: GAT-seeded adversarial generation**

11: Initialize Generator $G_{\theta_{g}}$ and Discriminator $D_{\theta_{d}}$ with random weights.

12: **for** iteration = 1 **to** *T*_GAN_ **do**

13:  *Train Discriminator for step = 1 to* $n_{d}$

14:  **for** step =1 **to** $n_{d}$ **do**

15:   Sample a batch of real data $\left\{{𝐱}^{\left(1\right)},...,{𝐱}^{\left(m\right)}\right\}$ from 𝐗.

16:   $m_{seed}\leftarrow \lfloor m\cdot \lambda \rfloor$; $m_{noise}\leftarrow m-m_{seed}$.

17:   Sample $m_{seed}$ GAT-prioritized seed cells $\left\{{𝐱}_{seed}^{\left(1\right)},...,{𝐱}_{seed}^{\left(m_{seed}\right)}\right\}$ from ${𝐗}_{new}$.

18:   Sample $m_{noise}$ random noise vectors $\left\{{𝐳}^{\left(1\right)},...,{𝐳}^{\left(m_{noise}\right)}\right\}$ from $p_{z}(𝐳)$.

19:   Construct the batch-level hybrid input ${𝐙}^{\prime }\leftarrow \{{𝐱}_{seed}^{\left(1\right)},...,{𝐱}_{seed}^{\left(m_{seed}\right)}\}\cup \{{𝐳}^{\left(1\right)},...,{𝐳}^{\left(m_{noise}\right)}\}$.

20:   Shuffle ${𝐙}^{\prime }$ before feeding it to the generator.

21:   Generate fake samples ${𝐗}_{\text{fake}}\leftarrow G_{\theta_{g}}({𝐙}^{\prime })$.

22:   Update discriminator parameters $\theta_{d}$ by ascending the stochastic gradient of:

$\nabla_{\theta_{d}}\;\frac{1}{m}\sum_{i=1}^{m}[\log D_{\theta_{d}}({𝐱}^{\left(i\right)})+\log(1-D_{\theta_{d}}({𝐗}_{\text{fake}}^{\left(i\right)}))].$

23:  **end for**

24:  *Train Generator for step = 1 to* $n_{g}$

25:  **for** step =1 **to** $n_{g}$ **do**

26:   Sample and construct a new hybrid input batch ${𝐙}^{\prime }$ of size *m*.

27:   Update generator parameters $\theta_{g}$ by descending the stochastic gradient of:

$\nabla_{\theta_{g}}\;\frac{1}{m}\sum_{i=1}^{m}\log(1-D_{\theta_{d}}(G_{\theta_{g}}({𝐙}^{\prime \left(i\right)}))).$

28:  **end for**

29: **end for**

30: **return** Trained Generator $G_{\theta_{g}}$

### Empirical assessment of training stability

We empirically evaluated whether attention-guided leakage improves the stability of adversarial training. Specifically, we compared the no-leakage baseline (λ=0) with GARAGE under leakage settings λ∈{0.10,0.20,0.30} using the same generator and discriminator architecture, optimizer settings, preprocessing pipeline, and training schedule. The only difference between the baseline and GARAGE was the construction of the generator input batch: the baseline used random noise only, whereas GARAGE used a batch-level mixture of random noise vectors and GAT-prioritized seed cells.

For each dataset and leakage setting, we monitored the generator loss and discriminator loss across training iterations. We also quantified training stability by measuring loss oscillation over the final training window and by computing the final distributional similarity between real and generated cells. Lower loss oscillation indicates more stable adversarial training, while lower distributional distance indicates better agreement between real and generated transcriptomic distributions.

This analysis evaluates whether attention-guided leakage provides empirical stabilization relative to a conventional noise-driven GAN. The complete loss curves and run-wise stability summaries and detail explanations are provided in subsection “Training-stability ablation,” Supplementary Table (10) and Supplementary Figure (34) in S1 File.

**Practical implication of the convergence analysis.** The above analysis provides an intuitive explanation for the empirical stability of GARAGE. In a conventional GAN, the generator initially receives only random noise, and many generated samples may lie far from the real single-cell manifold, producing unstable discriminator feedback and increasing the risk of mode dropping. In GARAGE, a controlled fraction of the generator input is anchored by GAT-prioritized real-cell seeds. These seeds provide biologically meaningful reference points that guide the generator toward under-represented but valid regions of the data manifold. Consequently, the generator receives more informative gradients, the discriminator is less likely to saturate early, and adversarial training becomes more stable. Thus, the theoretical analysis supports the practical observation that attention-guided leakage can improve both convergence behavior and rare-cell preservation during synthetic scRNA-seq data generation.

## Results and discussion

In this section, first, we will provide an overview the of some popular scRNA-seq datasets and the preprocessing techniques employed in the present study. Thereafter, we will discuss the evaluation report of our proposed GARAGE model using different setups on the scRNA-seq datasets. Subsequently, we will compare our model with five state-of-the-art methods by utilizing some benchmark gene selection techniques, followed by clustering the generated data.

### Overview of datasets

To comprehensively evaluate the performance of our proposed method, **four public single-cell RNA-seq (scRNA-seq)** datasets were utilized: Yan, CBMC, Pollen, and Muraro. These datasets were chosen to span a range of biological systems, cell counts, gene dimensionalities, and class complexities, thereby providing a robust benchmark for generative models. A summary of their properties is provided in Table 1.

**Table 1:** Summary of the datasets utilized here.

Datasets	Gene Expression	Sample Number	Class Types
Yan	10564	90	6
CBMC	2000	7895	13
Pollen	9665	299	11
Muraro	19127	2126	10

#### Yan dataset.

The Yan dataset (Yan et al. [32]) originates from a study of human preimplantation embryos, capturing transcriptomic profiles of single cells from oocytes to blastocysts. The dataset used in our study comprises of 90 cell samples across 10,564 genes, which are annotated into six distinct classes representing key stages of early embryonic development. Due to its clear, well-separated cell states and relatively low cell count, the Yan dataset serves as a fundamental benchmark for evaluating a model’s ability to capture the primary structure and variance of a biological process.

#### CBMC dataset.

The CBMC (cord blood mononuclear cells) dataset (Stoeckius et al. [33]) provides a detailed snapshot of the immune cell landscape in human cord blood. It is a larger-scale dataset consisting of 7,895 cells which are categorized into 13 distinct cell types, including various T-cells, B-cells, and natural killer cells. The CBMC dataset presents a significant hurdle for generative models due to the complex hierarchy and subtle transcriptional differences among closely related immune cell subtypes, thus testing a model’s capacity for fine-grained and high-resolution data generation.

#### Pollen dataset.

The Pollen dataset (Pollen et al. [34]) is derived from a study of human pluripotent stem cells differentiating into various lineages. The version used in our study contains 299 cell profiles across 9,665 genes and is grouped into 11 distinct cell types. Its key characteristic is the relatively low cell count compared to the high number of gene features. This makes it an ideal test case for assessing a model’s ability to learn a complex, high-dimensional distribution from limited data (HDSS), which presents a primary motivation for the use of synthetic data generation in bioinformatics.

#### Muraro dataset.

The Muraro dataset (Muraro et al. [35]) is a seminal resource for studying the cellular composition of human pancreatic islets. It comprises 2,126 individual pancreatic cells with measurements for 19,127 genes, annotated into 10 endocrine and other cell types. Its high gene dimensionality and the presence of both rare and abundant, well-characterized cell types make it a robust benchmark for evaluating a model’s scalability and its ability to preserve the complex, multi-modal structure of a real biological tissue. For a detailed description of the datasets, please visit the Supplementary Note (1) in S1 File.

### Data preprocessing

Prior to model training, each scRNA-seq dataset underwent a standardized preprocessing pipeline to mitigate technical noise and ensure data quality (Hwang et al. [36], Tian et al. [37]), a critical step for the stability and accuracy of downstream graph construction and adversarial learning. The raw gene expression count matrices were subjected to a series of quality control (QC) and normalization steps.

First, the QC filtering is applied at the cell level. Cells with an unusually low number of expressed genes or a high percentage of mitochondrial gene counts—often indicative of low-quality captures or apoptotic cells—were removed (Hwang et al. [36]).

Following QC, the filtered count data employed a standard log-normalization approach: the counts for each cell were divided by the total counts for that cell, multiplied by a scale factor (here, 10,000), and then log-transformed with a pseudocount of one (log1p). Let 𝐗 be the raw gene expression count matrix, where $x_{ij}$ is the count of gene *j* in cell *i*. The normalized expression value, ${\hat{x}}_{ij}$, is calculated as:


$$\begin{array}{c} {\hat{x}}_{ij}=\log\left(1+\frac{x_{ij}}{\sum_{k=1}^{M}x_{ik}}\times S\right) \end{array}$$ (10)

where $\sum_{k=1}^{M}x_{ik}$ is the total number of counts for cell *i* across all *M* genes, and *S* is a scale factor, here 10,000.

For the underlying preprocessing for scRNA-seq data, we used scanpy version 1.9.6 (Py version 3.9.21) (Wolf et al. [38]).

The outcome of this pipeline is a clean, normalized, and quality-controlled gene expression matrix, which serves as the foundational input for constructing the cell neighbourhood graph and training the *GARAGE* model.

### Quantitative benchmarking of GARAGE against leading state-of-the-art models

To rigorously validate the performance of GARAGE, we expanded the benchmarking framework to include three groups of baselines. The first group contains generic generative models, including standard GAN, VAE, f-GAN, and WGAN. The second group contains scRNA-seq-specific generative models, including LSH-GAN, scGAN/cscGAN, scVAE, and a diffusion-based single-cell generative model. The third group contains simple rare-cell balancing controls, including standard GAN and VAE trained on class-balanced or artificially oversampled versions of the original data. These oversampling baselines were included to test whether GARAGE provides benefits beyond simply increasing the representation of rare cell types in the training set.

The oversampling controls were constructed by randomly upsampling minority cell types in the training data until each class reached the size of the largest class or a predefined balancing threshold. Standard GAN and VAE models were then trained on these balanced datasets using the same preprocessing and downstream evaluation pipeline as GARAGE. These baselines are denoted as GAN + ROS and VAE + ROS, respectively. This comparison directly evaluates whether graph-attention-guided seed selection provides an advantage over simple class balancing. The detailed comparison with the additional scRNA-seq-specific baselines and oversampling controls is provided in Supplementary Tables (6) and (7) in S1 File.

The novelty of our GARAGE framework lies in its unique two-stage architecture, which fundamentally rethinks the input to the generative process. While standard GANs and their variants learn to map a random latent space to the data distribution, GARAGE first employs a graph attention network (GAT) as an intelligent selection module. The GAT is trained on a k-NN graph (Dong et al. [18]) of the real data to identify a core subset of samples with the highest attention scores, indicating their importance in defining cell-type identities and the overall data manifold. These attention-driven nodes are then used to **“seed”** the GAN’s generator by being mixed with random noise in the input batch. This intelligent seeding strategy directs the generative process to known, high-quality cell states that enhance the data generation of synthetic data.

To ensure a fair and robust comparison, all SOTA models were trained on default parameters (stated in the Supplementary Note (3.5) in S1 File) and evaluated under a standardized protocol across four widely used scRNA-seq datasets: Yan, Pollen, CBMC, and Muraro (stated in Table 2). Performance was quantified using three key metrics: the Adjusted Rand Index (ARI) and Normalized Mutual Information (NMI) to assess clustering fidelity, and the macro-F1 score to evaluate the balance and class accuracy after clustering. Also, each model has been assessed under two feature selection methods (Fano index and CV2) and on three data regimes, namely generated, original, and combined (generated + original). This rigorous benchmarking framework allows us to directly compare the capacity of each generative model to preserve the complex biological signals in single-cell data.

**Table 2:** Performance comparison across datasets, generative methods, data types, feature selection methods, and metrics. For readability, the best-performing value within each dataset-specific comparison block is highlighted.

Dataset	Generator	Feature Selection: Fano Index									Feature Selection: CV2								
		Generated			Combined			Original			Generated			Combined			Original		
		ARI	NMI	F1	ARI	NMI	F1	ARI	NMI	F1	ARI	NMI	F1	ARI	NMI	F1	ARI	NMI	F1
Yan	LSH-GAN	0.845	0.862	0.143	0.803	0.835	0.111	0.714	0.751	0.044	0.717	0.822	0.192	0.874	0.907	0.067	0.819	0.913	0.278
	VAE	0.682	0.819	0.016	0.759	0.827	0.215				0.708	0.786	**0.192**	0.718	0.872	0.143			
	FGAN	0.89	0.905	0.148	**0.845**	0.862	0.0				0.798	0.841	0.067	0.807	0.845	0.067			
	GAN	0.867	0.886	0.059	0.767	0.823	0.057				0.738	0.823	0.143	0.742	0.877	0.25			
	WGAN	0.842	0.852	0.078	0.759	0.817	0.211				0.845	0.862	0.067	0.742	0.877	0.25			
	**GARAGE**	**0.935**	**0.93**	**0.307**	0.837	**0.871**	**0.243**				**0.937**	**0.971**	0.167	**0.973**	**0.971**	**0.167**			
Pollen	LSH-GAN	0.833	0.884	0.057	0.542	0.657	0.006	0.346	0.605	0.101	0.735	0.891	0.097	0.735	0.891	0.054	0.689	0.871	0.045
	VAE	0.159	0.289	0.013	0.216	0.38	**0.109**				0.798	0.883	0.002	0.744	0.861	0.026			
	FGAN	0.735	0.767	0.108	0.718	0.791	0.013				0.559	0.695	**0.097**	0.694	0.772	**0.093**			
	GAN	0.656	0.722	0.061	0.277	0.475	0.033				0.662	0.743	0.031	0.856	0.935	0.061			
	WGAN	0.783	0.815	**0.227**	0.279	0.478	0.013				0.612	0.777	0.06	0.735	0.891	0.043			
	**GARAGE**	**0.882**	**0.896**	0.035	**0.827**	**0.84**	0.006				**0.926**	**0.923**	0.082	**0.936**	**0.943**	0.062			
CBMC	LSH-GAN	0.163	0.258	0.068	0.116	0.235	0.043	0.103	0.208	0.021	0.47	0.535	0.024	0.394	0.453	0.015	0.108	0.217	0.044
	VAE	0.359	0.452	0.068	0.268	0.375	0.02				0.295	0.439	0.024	0.262	0.339	0.071			
	FGAN	0.299	0.286	0.108	0.142	0.266	0.033				0.13	0.188	0.065	0.258	0.292	0.021			
	GAN	0.276	0.357	0.108	0.318	0.358	**0.074**				0.449	0.447	0.069	0.432	0.514	0.021			
	WGAN	0.152	0.19	0.108	0.077	0.189	0.043				0.317	0.3	**0.069**	0.301	0.104	0.036			
	**GARAGE**	**0.541**	**0.613**	**0.13**	**0.512**	**0.576**	0.015				**0.542**	**0.595**	0.027	**0.445**	**0.508**	**0.067**			
Muraro	LSH-GAN	0.198	0.372	0.055	0.154	0.215	0.115	0.204	0.371	0.044	0.476	0.705	0.12	0.236	0.444	0.042	0.24	0.408	0.042
	VAE	0.012	0.027	0.022	0.002	0.014	0.115				0.256	0.445	0.104	0.237	0.448	0.048			
	FGAN	0.221	0.412	0.055	0.216	**0.366**	0.115				0.309	0.53	0.114	0.265	0.456	0.042			
	GAN	0.101	0.218	0.055	0.097	0.194	0.115				0.149	0.241	0.034	0.258	0.444	0.141			
	WGAN	0.188	0.33	0.166	0.007	0.019	0.115				0.188	0.351	**0.114**	0.246	0.43	0.042			
	**GARAGE**	**0.375**	**0.483**	**0.205**	**0.245**	0.341	**0.123**				**0.528**	**0.639**	0.047	**0.565**	**0.714**	**0.283**			

Across the benchmark datasets, GARAGE showed stronger performance than the evaluated generic generative models, scRNA-seq-specific generative models, and oversampling-based controls under the same preprocessing and downstream evaluation pipeline (Table 2). In particular, GARAGE achieved the highest cluster fidelity in terms of Adjusted Rand Index (ARI) and Normalized Mutual Information (NMI) across most of the datasets. For example, on the relatively straightforward Yan dataset, GARAGE nearly attained perfect clustering alignment (ARI ≈ 0.97, NMI ≈ 0.97 using CV2-selected features), substantially surpassing the best baseline (LSH-GAN, ARI ≈ 0.87, NMI ≈ 0.91). Similarly, in the more heterogeneous Pollen data, GARAGE reached ARI scores up to 0.93 (considering *CV*2 feature selection method), again exceeding the strongest alternative model by a wide margin (LSH-GAN at ARI 0.83). Even on the most challenging benchmarks, such as CBMC and Muraro datasets, where all methods showed lower absolute performance due to complex, imbalanced cell populations, GARAGE maintained a clear advantage. Notably, on CBMC its synthetic data obtained the highest ARI (0.54 vs. 0.47 for the best baseline) and NMI (0.61 vs. sub-0.54 for others), and on Muraro GARAGE likewise topped the baselines with ARI around 0.53 (compared to 0.48 from the next-best model). These improvements in ARI/NMI indicate that GARAGE-generated cells cluster with greater purity and alignment to ground-truth labels than those produced by any competing method, across datasets ranging from clean and simple to noisy and complex. For *GARAGE*, the parameters for ARI, NMI, F1, and all additional UMAP results are provided in the Supplementary Note (4) in S1 File.

Also, it is noteworthy that GARAGE showed better balancing when detecting all cell subpopulations, as evidenced by the highest macro-F1 scores at all times. The macro-F1 of GARAGE was always better than that of any other model and proved the ability of our model to accurately reproduce all types of cells, including the minority ones. For example, in the Yan dataset, the GARAGE macro-F1 score was 0.307, while in others, the scores were significantly lower (usually less than 0.15-0.20). This means that our approach does not sacrifice small clusters in favor of the larger ones. The same applies to the most difficult CBMC and Muraro datasets, in which the macro-F1 scores for GARAGE were 0.13 and 0.28, respectively. Again, we see that this score is better than any baseline and shows much more balanced detection of cell types. This is an important distinguishing characteristic because while the standard GAN and VAE generate mostly samples from the most represented cell states (hence poor recall of the minority cells), the attention-guided seeding in GARAGE ensures that even rare subpopulations are learned and reproduced.

To assess the sensitivity of the quantitative results to stochastic variation, we performed an additional repeated-run stability analysis for the main comparison setting. The full pipeline was repeated across independent random seeds for GARAGE and its competing baselines, including LSH-GAN, GAN, FGAN, and WGAN. For each run, model initialization, synthetic-data generation, and clustering initialization were varied, and ARI and NMI were summarized as mean ± standard deviation. These results are provided in Table 3.

**Table 3:** Repeated-seed stability analysis for the main comparison setting. Values are reported as mean ± standard deviation across independent random seeds.

Dataset	Method	ARI	NMI
Yan	GAN	0.8187 ± 0.0458	0.8503 ± 0.0203
Yan	WGAN	0.7462 ± 0.1101	0.8033 ± 0.0733
Yan	FGAN	0.7493 ± 0.0680	0.8037 ± 0.0487
Yan	LSH-GAN	0.7544 ± 0.0645	0.8156 ± 0.0433
Yan	GARAGE	0.9169 ± 0.0502	0.9210 ± 0.0408
Pollen	GAN	0.7245 ± 0.0096	0.8166 ± 0.0267
Pollen	WGAN	0.5954 ± 0.0914	0.7063 ± 0.0733
Pollen	FGAN	0.6719 ± 0.0516	0.7654 ± 0.0326
Pollen	LSH-GAN	0.6977 ± 0.1299	0.8063 ± 0.0509
Pollen	GARAGE	0.8812 ± 0.0500	0.9052 ± 0.0502
CBMC	GAN	0.2056 ± 0.0440	0.3781 ± 0.0527
CBMC	WGAN	0.3330 ± 0.0073	0.4346 ± 0.0156
CBMC	FGAN	0.1706 ± 0.1023	0.3072 ± 0.0848
CBMC	LSH-GAN	0.1896 ± 0.0193	0.3850 ± 0.0246
CBMC	GARAGE	0.4651 ± 0.0261	0.4656 ± 0.0505
Muraro	GAN	0.0964 ± 0.0100	0.2249 ± 0.0125
Muraro	WGAN	0.1820 ± 0.1245	0.3013 ± 0.1277
Muraro	FGAN	0.3548 ± 0.0960	0.5114 ± 0.0719
Muraro	LSH-GAN	0.3178 ± 0.3036	0.4490 ± 0.3503
Muraro	GARAGE	0.6136 ± 0.2136	0.6439 ± 0.1516

Overall, these benchmarking results establish GARAGE as a robust, generalizable generative framework that delivers state-of-the-art performance across diverse conditions. Its improvements were consistent under both gene selection strategies tested (Fano index and *CV*2) and were evident whether we evaluated only generated cells or a combined set of generated+original cells. In all scenarios, GARAGE not only surpassed the other generative models in cluster quality (achieving ARI/NMI values close to the theoretical maximum in easy cases, and the highest scores in difficult cases) but, in some instances, even matched or exceeded the clustering performance of the original dataset itself. These results suggest that GARAGE’s two-stage design, which leverages a araph attention network to seed the generator with high-value cell states, yields synthetic data that preserves the complex structure of single-cell transcriptomes with exceptional fidelity. The ability to maintain high cluster integrity and balanced class representation across multiple datasets and experimental settings underscores GARAGE’s potential for generating realistic single-cell data. The UMAPs with their respective ARI, NMI, and macro-f1 score for all the SOTA models of all four scRNA-seq datasets are provided in the Supplementary Note (5) in S1 File.

### Clustering evaluation using fixed biological marker genes

To disentangle the contribution of the generative model from the feature selection step, we performed an additional clustering analysis using a fixed biologically relevant marker-gene set for each dataset. In the previous analysis, feature selection was performed using Fano index and CV2, and therefore the reported ARI, NMI, and macro-F1 scores reflected the combined effect of generation, feature selection, and clustering. In contrast, the present analysis uses the same predefined marker-gene set for all generative methods within each dataset. These marker genes were selected independently of the generated data, based on known cell-type markers and/or marker information reported in the original dataset studies.

Using these fixed marker genes, we repeated the same clustering evaluation for real, generated, and combined datasets. Because the feature set was held constant across methods, differences in ARI and NMI more directly reflect the ability of each generative model to preserve biologically meaningful cell-type structure rather than differences introduced by feature selection. The full marker-gene lists and the corresponding fixed-marker clustering results are provided in Supplementary Tables (8) and (9) in S1 File.

### Biological validation of GAT-prioritized high-attention cells

To provide biological context for the cells selected by the GAT module, we performed a post hoc marker-gene validation on the CBMC dataset. This dataset was chosen because it contains diverse immune and progenitor populations, including both common and low-frequency cell states. After GAT training, cells were ranked according to their aggregated attention scores, and the top-20% and bottom-20% of cells were defined as high-attention and low-attention subsets, respectively. We then examined whether known cell-type marker genes showed elevated expression in the high-attention subset.

The analysis included 24 literature-supported marker genes representing eight immune or progenitor populations. For each marker gene, we computed the mean expression in all cells, high-attention cells, and low-attention cells, followed by $\log_{2}$ fold-change and Wilcoxon rank-sum testing between the high- and low-attention subsets. The marker genes were used only for post hoc biological interpretation and were not used to train the GARAGE model.

The high-attention subset showed consistently elevated marker expression relative to the low-attention subset. All 24 examined markers had positive $\log_{2}$ fold-change, and 15 markers reached Wilcoxon significance (*p* < 0.05). Significant markers included rare or distinctive population markers such as *HBA2* and *HBB* for erythroid cells, *PF4* and *PPBP* for megakaryocytes, *LILRA4*, *CLEC4C*, and *TCF4* for plasmacytoid dendritic cells, *CD34* and *KIT* for hematopoietic progenitors, and *CLEC10A* for conventional dendritic cells. Markers of common immune populations, including *S100A8*, *CST3*, and *CD14* for monocytes, *MS4A1* and *CD79A* for B cells, and *KLRF1* for NK cells, also showed increased expression in high-attention cells. These results indicate that GAT-prioritized cells capture biologically interpretable immune and progenitor signatures, supporting their use as biologically informative anchors for attention-guided leakage in GARAGE. The complete marker-expression summary is provided in Supplementary Table 11 in S1 File.

### Label-agnostic and label-scarce validation of GARAGE

Because the GAT module uses cell-type labels during training, we further evaluated GARAGE using validation strategies that do not rely solely on the same labels used for attention-guided seed selection. In addition to ARI, NMI, and macro-F1, we computed label-agnostic distributional similarity metrics between real and generated data on the full preprocessed gene-expression matrix, before feature selection. Specifically, we used Maximum Mean Discrepancy (MMD) with an RBF kernel and Sliced Wasserstein Distance (SWD) over random projections of the complete gene-expression space. These metrics assess whether the generated cells match the real transcriptomic distribution without using cell-type labels or clustering assignments. The full label-agnostic distributional similarity results are provided in Supplementary Table (4) in S1 File.

For two empirical distributions of real cells $𝒳=\{{𝐱}_{i}{\}}_{i=1}^{n}$ and generated cells $𝒴=\{{𝐲}_{j}{\}}_{j=1}^{m}$, the unbiased squared MMD was computed as


$$\begin{array}{c} {MMD}^{2}(𝒳,𝒴)=\frac{1}{n(n-1)}\sum_{i\neq i^{\prime }}k({𝐱}_{i},{𝐱}_{i^{\prime }})+\frac{1}{m(m-1)}\sum_{j\neq j^{\prime }}k({𝐲}_{j},{𝐲}_{j^{\prime }})-\frac{2}{nm}\sum_{i=1}^{n}\sum_{j=1}^{m}k({𝐱}_{i},{𝐲}_{j}), \end{array}$$

where k(·,·) is an RBF kernel and the kernel bandwidth was selected using the median heuristic. Lower MMD values indicate stronger similarity between the real and generated distributions. To complement MMD, we also computed SWD by projecting real and generated cells onto multiple random one-dimensional directions and averaging the one-dimensional Wasserstein distances across projections. This provides a scalable distributional comparison in the full gene-expression space.

To further assess utility in a realistic label-scarce setting, we performed a held-out rare-cell experiment. For each dataset, a rare or low-frequency cell type was selected, and a subset of its real cells was withheld from GARAGE training and used only for testing. Synthetic cells generated by GARAGE were then used to augment the limited labelled training set, and a downstream classifier was trained on the augmented data. The classifier was evaluated on the held-out real cells. This setup tests whether GARAGE-generated samples improve recognition of rare cell types in unseen real data, rather than merely improving label agreement on the training distribution. The complete held-out rare-cell utility results are reported in Supplementary Table (5) in S1 File.

### Analysis of leakage sensitivity of GARAGE across datasets

We systematically assessed the performance of **GARAGE model** across four single-cell RNA-seq (scRNA-seq) datasets, namely Yan, Pollen, CBMC, and Muraro, under three leakage levels conditions (10%, 20%, 30%). Therefore, at the time of synthetic scRNA-seq data generation, with this innovative approach, we combine the random noise with our “attention-weighted leakage.” To quantitatively observe the performance, we evaluated the fidelity of generated data over original data using the Wasserstein Distance (WD) (Ruschendorf et al. [39]), where the lower values signify a stronger similarity between the two distributions. Please refer to the Supplementary Note (3.4) in S1 File for the WD parameters of different leakage types.

As detailed in Table 4, we compared our model with different percentages of GAT-enabled real data leakage (10%, 20%, 30%) against a baseline with no leakage (0%). The results depict a clear and consistent improvement in WD when employing the seeded generation strategy. Across all diverse scRNA-seq datasets, the seeded generation produced better synthetic data, as a 0% leakage-based architecture, consistently producing the highest, and therefore worst, WD samples. The introduction of any level of data leakage gradually improved the quality of data generation and consequently decreased the WD between real and generated data. This suggests that our **GARAGE** framework, by guiding the generator with a curated set of real data samples, provides a robust and significant improvement over the traditional methods in synthetic scRNA-seq data generation.

**Table 4:** Wasserstein distance for varying leakage percentages across datasets.

Dataset	No Leakage (0%)	10%	20%	30%
Yan	4.2016	2.0277	1.9218	**1.6862**
Pollen	1.4514	1.2982	**1.2185**	1.4592
CBMC	0.0044	**0.0020**	0.0032	0.0033
Muraro	0.0571	**0.0415**	0.0434	0.0441

For more robustness, at the UMAP-based adjusted rand index(ARI), normalized mutual information(NMI) calculation, we saw the difference in clusters. The data cluster obtained from the generated data not only becomes separated, but also they are significant (in most cases). We performed two feature selection (FS) strategies (Fano index and CV2). The ARI, NMI and macro F1-score were the performance metrics used to facilitate the evaluation on three kinds of dataset samples - generated, real, and combined(generated + real). The methodical structure provides insight into understanding the role played by the intrinsic structure of each dataset and the adopted feature selection method, under controlled leakage levels, in influencing generative fidelity and manifold stability.

*GARAGE* was evaluated with attention-weighted leakage of 10%, 20%, and 30% over four scRNA-seq datasets (Yan, Pollen, CBMC, Muraro) for both distributional fidelity using Wasserstein Distance (WD) and clustering performance using ARI, NMI, and macro-F1 in combination with Fano and CV2 feature selection applied on generated, original, and concatenated (generated + original) data (Tables 4 and 5). The following observations were made. In comparison with the no-leakage setting, any positive level of leakage caused a decrease in WD, implying better approximation of synthetic by real distributions; the largest effect of WD reduction was observed at an intermediate leakage value. For example, Yan had WD values 4.20 and 1.69 for 0% and 30% leakages, respectively; Pollen did not exhibit a monotonic WD dependence on leakage but had the smallest WD (1.22) at 20%. Finally, in the CBMC dataset where the difference in global distributions was quite small, leakage of 10% additionally decreased WD (to 0.0020), suggesting that even limited seeding sharpens local distributional details.

**Table 5:** Clustering performance (ARI, NMI, Macro F1) for different datasets and leakage percentage(%) using Fano and CV2 feature selection.

Dataset	Leakage (λ)	Feature Selection using Fano Index									Feature Selection using CV2								
		Generated			Combined			Original			Generated			Combined			Original		
		ARI	NMI	F1	ARI	NMI	F1	ARI	NMI	F1	ARI	NMI	F1	ARI	NMI	F1	ARI	NMI	F1
Yan	λ=10%	0.823	0.859	0.063	0.837	0.871	0.243	0.714	0.751	0.044	0.884	0.889	0.154	0.898	0.927	0.292	0.819	0.913	0.278
	λ=20%	0.935	0.930	0.307	0.726	0.800	0.225				0.923	0.926	0.188	0.973	0.971	0.167			
	λ=30%	0.837	0.846	0.011	0.779	0.828	0.005				0.973	0.971	0.167	0.973	0.971	0.167			
Pollen	λ=10%	0.761	0.861	0.097	0.821	0.845	0.064	0.346	0.605	0.101	0.926	0.923	0.082	0.861	0.875	0.025	0.735	0.891	0.05
	λ=20%	0.882	0.896	0.035	0.827	0.84	0.006				0.732	0.838	0.108	0.732	0.763	0.052			
	λ=30%	0.779	0.824	0.144	0.308	0.449	0.096				0.808	0.835	0.079	0.936	0.943	0.062			
CBMC	λ=10%	0.371	0.305	0.025	0.395	0.363	0.028	0.103	0.208	0.021	0.533	0.550	0.056	0.413	0.380	0.010	0.108	0.217	0.044
	λ=20%	0.497	0.478	0.019	0.485	0.473	0.012				0.495	0.498	0.050	0.461	0.489	0.009			
	λ=30%	0.541	0.613	0.130	0.512	0.576	0.015				0.542	0.595	0.027	0.445	0.508	0.067			
Muraro	λ=10%	0.342	0.423	0.171	0.146	0.282	0.075	0.204	0.371	0.044	0.258	0.450	0.053	0.248	0.438	0.063	0.240	0.408	0.042
	λ=20%	0.375	0.483	0.205	0.234	0.326	0.051				0.528	0.639	0.047	0.565	0.714	0.283			
	λ=30%	0.173	0.299	0.016	0.245	0.341	0.123				0.510	0.598	0.130	0.385	0.523	0.114			

Clustering analyzes echoed these trends and clarified the role of leakage as a guidance signal that must be balanced against overfitting. On generated data, ARI and NMI typically rose from 0% to a moderate leakage level, often achieving near-perfect alignment on well-structured datasets with CV2 features (e.g., Yan ARI ≈0.973, NMI ≈0.971). Leakage also positively affected Macro-F1, which depends on the restoration of minority cells, with improvements being prominent while transitioning from 0% leakage to 10%-20%, as shown in high macro-F1 values for Muraro combined data at 20% (≈0.283). More difficult scenarios such as CBMC still saw the positive effect of leakage on generated-data clustering (e.g., peak ARI value around 0.54 at 30%), meaning that noisy manifolds might need more robust seeds from real signals in order to be separable. On the other hand, heterogeneous datasets, such as Pollen, were sometimes negatively impacted by excessive leakage (30%), especially on combined data—consistent with over-anchoring the generator to training labels and reducing generative flexibility.

To make the choice of leakage rate more interpretable, we consolidated the leakage-sensitivity results across datasets and related the observed trends to dataset-level properties such as class imbalance, rare-cell representation, and manifold complexity. The results suggest that λ should not be treated as a universal fixed hyperparameter. Moderate leakage values, particularly λ=0.10–0.20, provide a reasonable starting range for datasets with relatively clear subtype structure or moderate imbalance. For datasets with stronger imbalance or weaker cell-state separation, a larger value such as λ=0.30 may be beneficial because stronger real-cell anchoring can help the generator recover underrepresented regions of the manifold. However, increasing λ should be treated cautiously, because excessive leakage may over-anchor the generator to selected seed cells and reduce generative flexibility. The complete leakage-performance trends across datasets are summarized in Supplementary Figure (36) in S1 File.

**Sensitivity to leakage and seed-selection hyperparameters.** In addition to the leakage-rate analysis, we examined the sensitivity of GARAGE to the top-*q*% seed-selection threshold. The leakage rate λ controls the fraction of GAT-prioritized seed cells inserted into the generator input batch, whereas the top-*q*% threshold controls the size of the high-attention seed set ${𝐗}_{new}$. We varied these parameters while keeping the remaining model settings fixed. The *k*-NN graph size was fixed at $k_{nn}=5$ in all reported experiments because GARAGE relies on local neighbourhood structure for rare-cell-aware attention scoring, and varying $k_{nn}$ requires reconstructing the cell graph, retraining the GAT, recomputing the seed set, and rerunning adversarial training. The sensitivity results indicate that GARAGE is stable across moderate leakage and seed-selection ranges. Moderate seed-selection thresholds provide sufficient biological anchoring without over-constraining the generator, whereas overly large seed sets may reduce generative flexibility. The detailed sensitivity results are provided in the subsection “Focused hyperparameter sensitivity analysis” and in Supplementary Table (12) in S1 File.

### UMAP-based evaluation of model performance

We further examined how attention-guided leakage shapes the geometry of the learned embeddings using UMAP, reporting ARI, NMI, and macro-F1 for generated, original, and combined (generated + original) data under both Fano Index and coefficient of variance (CV2) feature selection strategies (Figs 2–5). Across datasets, the visual and quantitative trends align with the leakage-sensitivity analysis: small to moderate leakage typically sharpens cluster boundaries and increases alignment with ground-truth labels. In contrast, excessive leakage can compress variability and introduce over-anchoring to the training labels.

**Fig 2:**
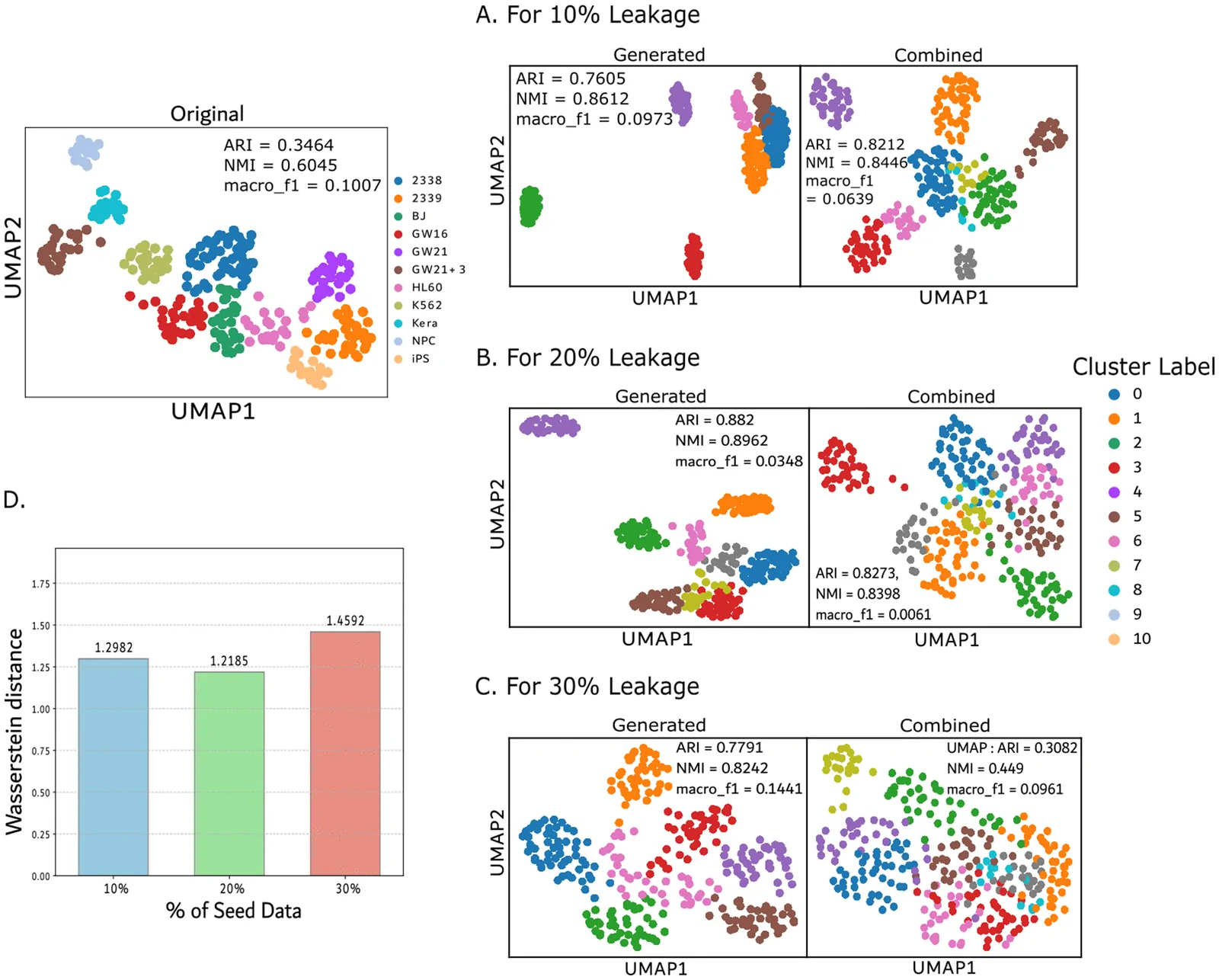
Leakage stress test on the Pollen dataset using the Fano index feature selection strategy. A: UMAP projections of real, generated, and combined data at 10% leakage, with clustering metrics (ARI, NMI, macro-F1) indicated. B: UMAP projections at 20% leakage with corresponding metrics. C: UMAP projections at 30% leakage with corresponding metrics. D: Distributional shift between real and generated data measured by the 1-Wasserstein distance across leakage levels (10%, 20%, 30%).

**Fig 3:**
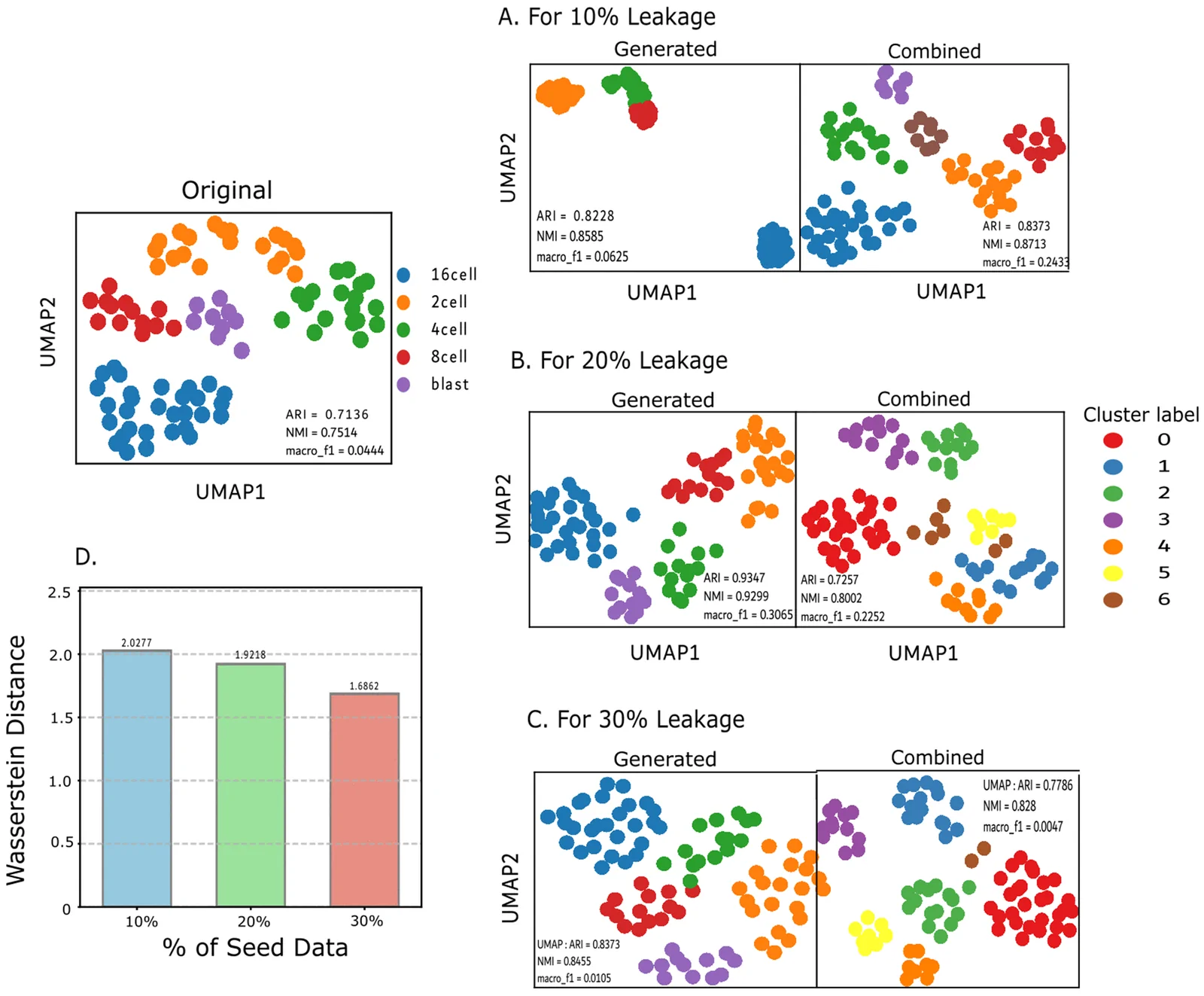
Leakage stress test on the Yan dataset using the Fano index feature selection strategy. A: UMAP projections of generated, and combined data at 10% leakage, with clustering metrics (ARI, NMI, macro-F1) indicated. B: UMAP projections at 20% leakage with corresponding metrics. C: UMAP projections at 30% leakage with corresponding metrics. D: Distributional shift between real and generated data measured by the 1-Wasserstein distance across leakage levels (10%, 20%, 30%).

**Fig 4:**
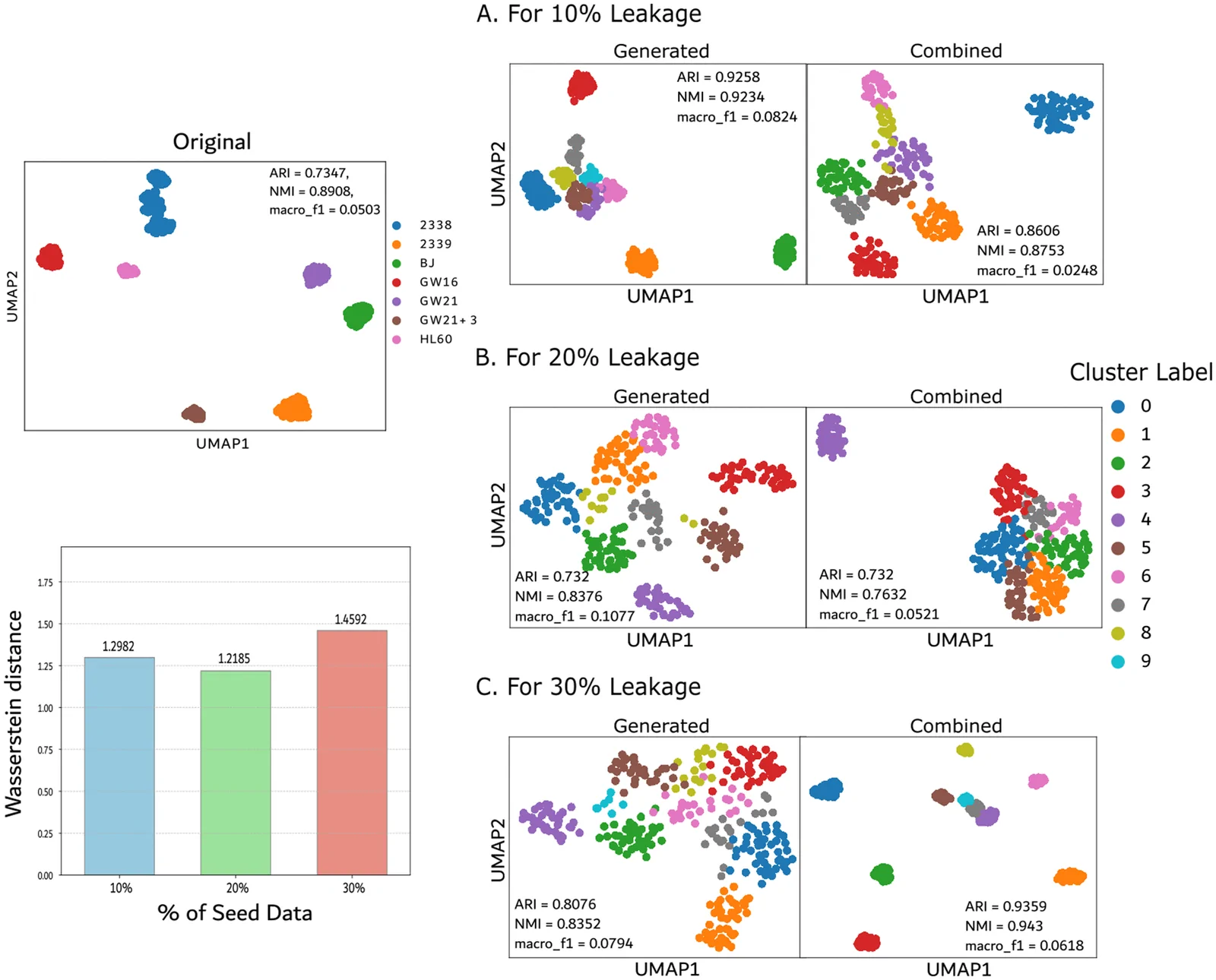
Leakage stress test on the Pollen dataset using the CV2 feature selection strategy. A: UMAP projections of generated and combined data at 10% leakage, with clustering metrics (ARI, NMI, macro-F1) indicated. B: UMAP projections at 20% leakage with corresponding metrics. C: UMAP projections at 30% leakage with corresponding metrics. D: Distributional shift between real and generated data measured by the 1-Wasserstein distance across leakage levels (10%, 20%, 30%).

**Fig 5:**
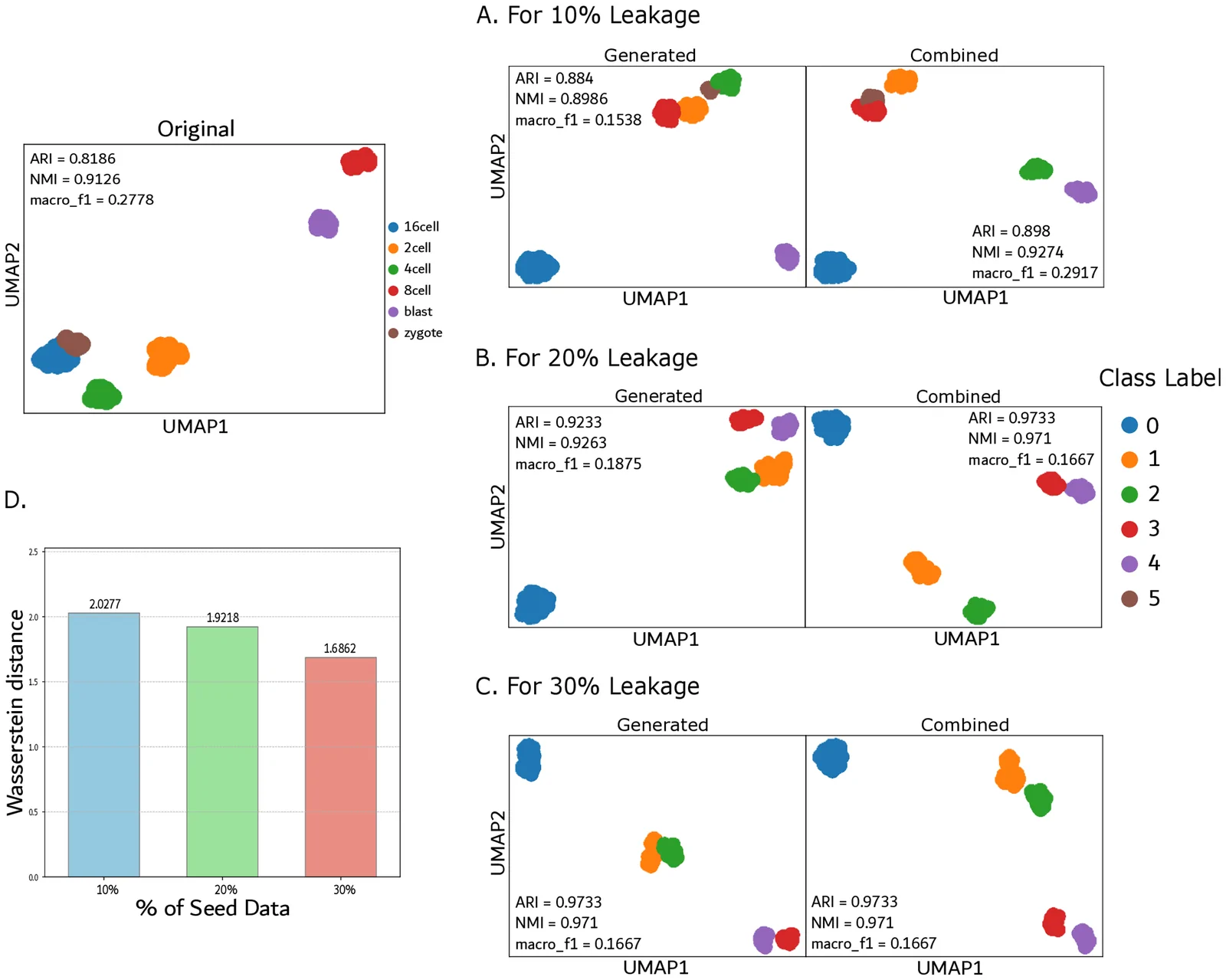
Leakage stress test on the Yan dataset using the CV2 feature selection strategy. A: UMAP projections of generated, and combined data at 10% leakage, with clustering metrics (ARI, NMI, macro-F1) indicated. B: UMAP projections at 20% leakage with corresponding metrics. C: UMAP projections at 30% leakage with corresponding metrics. D: Distributional shift between real and generated data measured by the 1-Wasserstein distance across leakage levels (10%, 20%, 30%).

#### Feature selection using Fano index.

Under Fano-selected genes, UMAP projections consistently show that introducing leakage transforms diffuse manifolds into compact, well-separated clusters for both well-structured and heterogeneous datasets. At 10–20% leakage, generated embeddings typically exhibit the largest gains in ARI/NMI with visible tightening of cluster cores and clearer inter-cluster margins; macro-F1 improves concurrently, indicating better recovery of minority populations. These effects are most apparent in datasets with strong intrinsic structure (e.g., Yan), where generated and combined embeddings approach near-perfect alignment at moderate leakage, and remain evident in more heterogeneous settings (e.g., Pollen), albeit with a non-monotonic response. At 30% leakage, several datasets display signs of over-anchoring: clusters become overly compact and inter-cluster geometry is partially distorted, which coincides with a drop in ARI/NMI and, in some cases, macro-F1. Distributional trends measured by 1-Wasserstein distance mirror the geometry: leakage lowers real–synthetic divergence most at moderate levels (e.g., Pollen’s WD decreases from 1.2982 at 10% to 1.2185 at 20% before worsening at 30%), while datasets with simpler geometry can tolerate larger leakage without severe degradation (e.g., Yan shows steadily decreasing WD from 2.0277 → 1.9218 → 1.6862).

#### Feature selection using CV2.

With CV2-selected genes, baselines are stronger and the leakage response is correspondingly smoother. Low leakage (10%) is often sufficient to align generated embeddings closely with ground truth while preserving manifold diversity; combined embeddings frequently improve further due to complementary coverage from real and synthetic cells. For moderate leakage (20%), GARAGE achieves optimal ARI/NMI performance and the best macro-F1 score for recovering rare cells, since it is capable of embedding minority groups while avoiding variability collapse. In certain heterogeneous conditions, a step-up to 30% forces an extremely rigid behavior of the combined embedding (higher ARI/NMI score in the mixture case), despite minimal gains in the generated embedding alone—a result of over-restrictive supervision forcing synthetic points into dominant real clusters. The behavior of WD is consistent with the former two metrics: drops between 10–20% represent good real-synthetic correspondence, while increases at 30% suggest overfitting to dataset-specific properties (Table 5).

Overall, the UMAP analyses confirm a common operating regime: attention-guided leakage reliably improves geometric fidelity and clustering agreement, with 10–20% striking the best balance in most datasets and CV2 providing a more stable, variance-aware substrate for embedding quality. Where manifolds are especially noisy, slightly higher leakage can be beneficial, but excessive guidance risks eroding generative flexibility and degrading the joint geometry. All the extended UMAPs and their detailed explanations are provided in the Supplementary Note (4) in S1 File.

## Conclusion

In this work, we introduced *GARAGE*, a novel two-stage generative framework designed to address the significant challenge of generating high-fidelity synthetic single-cell RNA-seq data. We identified a key limitation in traditional generative adversarial networks (GANs) - their potential for training instability and the generation of biologically implausible cell states when learning from a random noise distribution alone. To overcome this, GARAGE uniquely integrates a graph attention network (GAT) as an intelligent selection module to identify biologically representative “seed” cells, which are then used to guide and anchor the generative process of a subsequent GAN. From the experimental analysis, it can be concluded that GARAGE provides beyond satisfactory results across multiple datasets and performance metrics. Based on quantitative assessment using Wasserstein distance, the GARAGE variants (with 10–30% GAT-informed data leakage) demonstrate closer similarities to the real data consistently as opposed to synthesized samples generated by a traditional GAN (with 0% leakage) across all four benchmark samples. Furthermore, in a careful study comparing top generative models like VAE, FGAN, and WGAN, GARAGE consistently achieved the best results in downstream clustering fidelity. This was achieved using the adjusted Rand index (ARI), normalized mutual information (NMI), and macro-F1 score metrics. These findings indicate that not only do the datasets generated by GARAGE exhibit the statistical properties of real datasets, but the populations and cells’ identities remain intact, thus providing valuable data for biological studies.

The achievement of the GARAGE framework in terms of synthetic dataset generation has significant consequences for bioinformatics. First, it provides an effective technique for data augmentation, which will be useful in cases when it is hard to obtain a sufficient amount of data to analyze due to its scarcity. Second, it enables generating privacy-protected synthetic datasets that can be distributed to researchers to use for developing new techniques or testing different algorithms without endangering patient privacy. The capability of creating synthetic data that correctly reflects complicated population structures is an important milestone toward achieving more dependable and reproducible single-cell data analysis.

While there are many positive aspects of using GARAGE, we would like to emphasize several possible directions for further research in the future. One possible direction of future studies might concern the scalability of both k-NN graph construction and GAT training, which may become quite time-consuming when dealing with huge amounts of data. In addition, according to our findings, the optimal value of the leakage parameter λ varies depending on the specific dataset used; thus, a method for determining this hyperparameter could be considered as another possible direction for future research. Another interesting option for further investigation concerns expanding the scope of the GARAGE model to other single-cell techniques, such as scATAC-seq and spatial transcriptomics.

In conclusion, we consider GARAGE as a novel and highly efficient framework for the generation of synthetic single-cell data. By applying discriminative graph attention to the generation process, GARAGE offers an innovative method of overcoming the limitations of the previously developed techniques and sets the stage for more dependable use of the synthetic data in computational biology.

### Data and code availability

The benchmark datasets used in this study are publicly available from their original sources. The Yan dataset was obtained from Yan et al. [32]; the raw data are available through the Gene Expression Omnibus (GEO) under accession GSE36552. The CBMC dataset was obtained from the CITE-seq study of Stoeckius et al. [33]; the data are available through GEO under accession GSE100866, with associated BioProject PRJNA393315 and SRA accession SRP111194. The Pollen dataset was obtained from Pollen et al. [34]; the dataset is available through the Human Cell Atlas Data Portal under the project “Low-coverage single-cell mRNA sequencing reveals cellular heterogeneity and activated signaling pathways in developing cerebral cortex.” The Muraro dataset was obtained from Muraro et al. [35]; the raw data are available through GEO under accession GSE85241.

The GARAGE source code, preprocessing scripts, model-training scripts, synthetic-data generation scripts, biological validation scripts, and processed input files used directly by the models, where permitted by file-size constraints, are available in the project repository: https://github.com/RitwikGanguly/GARAGE. For public datasets not redistributed directly in the repository, the repository provides scripts or instructions to download the data from the original sources and reproduce the preprocessing workflow. The dedicated *GARAGE* documentation is available at https://garage-docs.readthedocs.io/en/latest/.

**Computational requirements and reproducibility settings.** All experiments were performed using Python-based implementations of the GARAGE pipeline. Two dedicated Conda environments–ritwik_base (Python 3.12.5) and scrna (Python 3.9.25)-were used to isolate the model-training and validation workflows, respectively. The corresponding dependency files are provided in the GitHub repository as requirements_garage.txt (model-training environment, including the core GARAGE pipeline, the state-of-the-art and single-cell-specific benchmarking baselines, the ablation studies, and the biological analysis modules) and requirements_validation.txt (validation environment, including feature selection, Leiden clustering, adjusted Rand index, normalised mutual information, macro-averaged F1-score, UMAP visualisation, Wasserstein distance, and marker-gene enrichment analysis).

The model-training environment (ritwik_base) was configured with Python 3.12.5, PyTorch 2.7.1 (CUDA 11.8), PyTorch Geometric 2.7.0, and NetworkX 3.6.1. The validation environment (scrna) was configured with Python 3.9.25, Scanpy 1.10.3 and other relevant packages present in the corresponding requirements file at GitHub.

All model-training experiments were performed on a single NVIDIA RTX A6000 GPU (48 GB VRAM, CUDA 11.8) with an AMD EPYC 7713P 64-core processor (64 threads) running Ubuntu 24.04 LTS and 314 GB system RAM. All the benchmarking datasets has been run on GPU for efficient training. Wasserstein distance computations and clustering evaluations were performed on CPU.

The repository README and the ReadTheDocs documentation (https://garage-docs.readthedocs.io/en/latest/) provide exact setup commands, per-script usage examples, expected outputs, and a step-by-step end-to-end tutorial for reproducing the core GARAGE results on the four built-in datasets (Yan, Pollen, CBMC, and Muraro). Users who wish to apply GARAGE to custom single-cell RNA-seq data are referred to the Preparing Your Data section of the documentation.

## Supporting information

S1 File Appendix. (PDF)
