# Supplementary material for: A graph-attentive GAN for rare-cell-aware single-cell RNA-seq data generation
Source: PLoS Comput Biol. 2026 Oct 5;22(10):e1014601. doi: 10.1371/journal.pcbi.1014601 (PMC13638049; doi:10.1371/journal.pcbi.1014601)
Supplement: Supplementary file 1 [file pcbi.1014601.s001.pdf]

A Graph-Attentive GAN for Rare-Cell–Aware Single-Cell RNA-Seq Data Generation

Ritwik Ganguly[1,2], Sana Aafrine[1], Sk Md Mosaddek Hossain[1,*], and Sumanta Ray[3,*]

[1]Computer Science and Engineering, Aliah University, Kolkata, India
[2]Computational Biology, Indraprastha Institute of Information Technology, New Delhi, India
[3]Data Science, The West Bengal National University of Juridical Sciences, Kolkata, India
[*]mosaddek.hossain@gmail.com, sumantaray@nujs.edu

1 Introduction

Recent breakthroughs in scRNA-seq have enabled us to detect intercellular heterogeneity at an unmatched level of detail. However, this technology faces two main challenges: the limited availability of samples across many experimental conditions and the significant imbalance between common and rare cell types. These challenges are particularly important in high-dimensional small sample (HDSS) contexts. In these situations, tasks like gene selection and clustering often suffer from reduced statistical power and skewed representations of cell populations (Vallejos et al. [1]). These problems underscore the need for computational methods that can generate realistic synthetic data to enhance limited datasets while accurately preserving the structure of rare subpopulations.

GARAGE is a novel approach that integrates graph-based attention with adversarial training to address problems in HDSS data and the sparse availability of rare cell types. The central concept is to leverage pre-existing knowledge from the cell–cell graph. Using Graph Attention Networks (GATs), GARAGE identifies underrepresented cells, particularly rare subpopulations, and retains them in training. In contrast to typical GANs, which rely solely on random noise, GARAGE supplies the generator with a chosen list of real cells with high attention scores. This data-driven approach accelerates convergence and stabilizes training.

We evaluated GARAGE on simulated and actual scRNA-seq data, with an emphasis on scenarios featuring imbalanced or sparse cell types. Across all our experiments, GARAGE generated data that better reflected the original distributions than other techniques. Additional analysis demonstrated its utility: feature selection identified more reliable marker genes, and clustering more accurately delineated cell-type structure and rare groups. These findings demonstrate both GARAGE's technical potential and its functional advantage for enhancing scRNA-seq analysis in cases of limited data.

In summary, GARAGE represents a principled step forward in generative modeling for single-cell biology. By weaving together graph attention and adversarial training, it introduces a biologically grounded mechanism for generating realistic and informative single-cell data. Our findings indicate that GARAGE can substantially enhance both the statistical power and biological resolution of downstream analyses, making it a versatile tool for researchers working with high-dimensional yet undersampled single-cell datasets.

2 Supplementary Note 1: Dataset Description

In our evaluation, we have utilized a total of four distinct datasets - YAN, MURARO, CBMC, and POLLEN, each selected for its unique relevance and contribution to our analysis. Below, we'd like to provide a brief overview of each dataset, highlighting its characteristics and the specific aspects it addresses within our research framework. This comprehensive approach allows us to draw more nuanced insights and ensures that our findings are well-rounded and robust.

2.1 YAN Dataset

The YAN dataset (Yan et al. [2]) is a scRNA sequencing dataset that focuses on early embryonic development. It includes 90 cells measured across 10,654 genes, capturing detailed transcriptional states. The cells represent six consecutive stages of development: blastocyst, 16-cell, 8-cell, 4-cell, 2-cell, and zygote. This tracks the progression from a single cell to a multicellular structure. Overall, YAN provides valuable insights into the earliest changes after fertilization.

2.2 CBMC Dataset

7,895 mononuclear cells from cord blood scRNA-seq data, each of which was examined for 2,000 specific genes, are included in the CBMC dataset (Stoeckius at al. [3]). The dataset includes both common and rare populations of immune and progenitor cells, such as progenitors, monocytes, B cells, NK cells, T cells, erythroid cells, and dendritic cells. Because of its diversity, the CBMC dataset is useful for assessing clustering algorithms, finding marker genes, and creating synthetic data—particularly when class imbalance is present.

2.3 MURARO Dataset

The Muraro dataset (Muraro et al. [4]) is an scRNA-seq resource cataloging human pancreatic islet cells, with 2,126 individual cells characterized across 18,902 gene features. It includes several endocrine and exocrine cell types, such as somatostatin-secreting δ cells, glucagon-secreting α cells, insulin-secreting β cells, pancreatic polypeptide (PP) cells, acinar cells, stellate cells, and ductal cells. This collection of data showcases the diverse range of cell types present in the pancreas. It includes data for 18,902 genes from a relatively small number of cells, making it a challenging test case for methods like dimensionality reduction, clustering, and generative modeling.

2.4 POLLEN Dataset

The Pollen dataset (Pollen et al. [5]) is an scRNA-seq dataset with 299 cells profiled along 9,665 gene features and is a collection of diverse human cell lines and primary cell types. It comprises fibroblasts, keratinocytes, neural progenitors, induced pluripotent stem cells, hematopoietic cell lines, and cells at various developmental stages. Its constitution covers biologically different and transcriptionally related populations, and thus it is a difficult target for clustering, classification, and generative modeling. Since there are significantly more genes than cells in the Pollen dataset, the data is sparse and difficult to identify distinct expression patterns without denoising or dimensionality reduction. In high-dimensional gene expression space, it is frequently used to assess how well computational techniques preserve overall population structure while capturing minute cell-type variations.

3 Supplementary Note 2: Models

3.1 Graph Attention Networks for Cell Prioritization in the GARAGE Framework

An important component of the GARAGE architecture is employing the use of GAT, a graph attention-based network, to recognize and rank highly biologically relevant cells. To interpret relations in scRNA-seq data, we initially construct a graph where every node is a cell, and edges connect cells sharing similar transcriptomic profiles. This graph enables us to locate both local and global relationships. It surpasses pairwise similarity alone, offering a deeper, richer structure for advanced deep learning paradigm.

Graph Neural Networks (GNNs) are suitable in this context. They apply updates to node representations by exchanging information with local nodes. One typical variant is the GCN - Graph Convolutional Network, which accumulates features between adjacent nodes, typically averaging or summing them. Although useful in a wide range of applications, regular GCNs are isotropic in their aggregation: they handle all neighboring nodes equally, regardless of their relative informativeness. In the case of scRNA-seq, in which some neighboring cells can capture biologically important variation

but others capture noise, doublets, or unimportant similarities, the homogeneous treatment can result in suboptimal embeddings and loss of important information regarding rare subpopulations.

The Graph Attention Network (GAT) generalizes the GCN formulation by adding a self-attention mechanism to the neighborhood aggregation step. Particularly, for every node, the GAT calculates attention coefficients that express the relative importance of every neighbor at feature aggregation. The attention coefficients are derived by feeding the concatenated features of a node and a neighbor through a shared attention function (usually a forward feeding neural layer accompanied by a LeakyReLU nonlinearity). The raw attention scores are subsequently normalized within the neighborhood using softmax as the function, resulting in a group of weights that sum up to one. The representation of a new node is calculated as a weighted aggregation of its neighbors' features. The neighbors that convey more informative information weigh more in the computation. This enables the model to attend to significant neighbors while dampening the effect of noisy or less informative signals.

The attention mechanism provides flexibility as well as interpretability. Flexibility is provided by learning task-specific weights rather than employing a shared strategy, which may vary across nodes and iterations of training. Interpretability assumes the form of attention coefficients, demonstrating which neighbors contribute most to the representation of a node. This is particularly beneficial in biological domains such as scRNA-seq analysis, where it helps to identify cells that predominantly define their local neighborhoods.

GARAGE employs this capability by first training the GAT on a supervised cell-type labeling problem. From this training, the model acquires attention coefficients that identify the most unique neighbors. Cells with high attention weights consistently are interpreted as conveying transcriptomic information relevant to determining close cell types. This approach is especially beneficial for under-represented or rare populations, which are frequently lost to uniform sampling or global clustering methods.

Following training, we compute the global importance score of each cell by summing up its attention coefficients across all neighborhoods it belongs to. We then sort the cells by these scores and take the top k% as priority nodes. This high-quality seed set (Xnew) comprises common and low-frequency cell types. It provides a significant blueprint aimed for the subsequent adversarial training step, ensuring that the generator retains and enhances important cellular states.

By directly pairing neighborhood focus with downstream cell selection, the GARAGE model guarantees that significant biological signals—most importantly, those from minor or functionally significant cells—are preserved and enhanced, as opposed to being watered down by homogeneous aggregation or random sampling. This combination of GAT-based prioritization with adversarial generation is one of the primary innovations in our method, unifying representation learning with biologically guided data curation.

Dynamic Neighboring Integration using Attention Mechanism

Graph Attention Networks (GATs) employ an adaptive weighting mechanism that allows each node to discern its neighbors' relative importance when aggregating information. The dynamic neighbor integration is achieved through an attention mechanism, which assigns higher weights to more relevant neighbors. The GAT framework generally consists of four key stages [6], which are outlined below.

1. Feature Projection At the start, every node i in the graph is represented by a characteristic vector $\mathbf{x}_i \in \mathbb{R}^F$, which reflects its inherent properties. These features are projected into a latent space using a shared linear transformation:

$$\mathbf{z}_i = \mathbf{W}\mathbf{x}_i \tag{1}$$

Here, $\mathbf{W} \in \mathbb{R}^{d\times F}$ is a learnable weight matrix that maps transforming the initial feature space into a d-dimensional embedded space, enabling comparisons and interactions across nodes. This step ensures that the model can capture meaningful cross-node relationships in a common representational space.

2. Pairwise Relevance Scoring Next, an attention mechanism computes the unnormalized affinity (or relevance) score between connected nodes (i, j):

$$s_{ij} = \text{LeakyReLU}\left(\boldsymbol{\theta}^\top(\mathbf{z}_i \oplus \mathbf{z}_j)\right) \tag{2}$$

where $\oplus$ denotes vector concatenation, and $\boldsymbol{\theta} \in \mathbb{R}^{2d}$ parameterizes the attention kernel. The LeakyReLU activation function, characterized by a small slope (typically around 0.2), allows gradients to flow even in negative regions, stabilizing training. This stage quantifies the relevance of each one of the neighbors in influencing the refined node representation i.

3. Stochastic Neighborhood Focusing To normalize the attention scores and allow meaningful comparisons, the raw scores are converted into attention coefficients using a masked softmax function:

$$\alpha_{ij} = \frac{\exp(s_{ij})}{\sum_{k \in \mathcal{M}_i} \exp(s_{ik})} \tag{3}$$

where $\mathcal{M}_i$ denotes the set of neighbors of node i. This ensures that $\sum_{j \in \mathcal{M}_i} \alpha_{ij} = 1$, effectively creating a probability distribution over neighbors. The masking ensures that only valid neighbors contribute to the attention computation.

4. Context-Aware Representation Finally, each node refreshes its representation by combining the features of its neighboring nodes, weighted by the attention coefficients:

$$\mathbf{z}'_i = \phi \left(\sum_{j \in \mathcal{N}_i \cup \{i\}} \alpha_{ij} \mathbf{z}_j \right) \tag{4}$$

where ϕ is a non-linear activation function, typically ELU, and self-connections (the term $i \in \mathcal{N}_i \cup \{i\}$) are included to allow nodes to preserve their own information during aggregation. This stage produces embeddings that are both neighborhood-aware and context-sensitive.

Multi-Head Attention

To capture diverse types of interactions and increase the model's expressive power, GATs employ multiple independent attention heads in parallel. Each head computes its own attention distribution and node embeddings:

$$\mathbf{z}'_i = \Big\Vert_{k=1}^{K} \phi \left(\sum_{j \in \mathcal{N}_i} \alpha_{ij}^{(k)} \mathbf{W}^{(k)} \mathbf{x}_j \right) \tag{5}$$

where $\Vert$ denotes concatenation and K is the number of attention heads. The resulting embeddings incorporate information from multiple perspectives, allowing the model to capture different types of node interactions. In the final layer, averaging rather than concatenation is often used to maintain dimensionality:

$$\mathbf{z}'_i = \phi \left(\frac{1}{K} \sum_{k=1}^{K} \sum_{j \in \mathcal{N}_i} \alpha_{ij}^{(k)} \mathbf{W}^{(k)} \mathbf{x}_j \right) \tag{6}$$

This multi-head design improves the robustness of the embeddings, reduces overfitting, and allows the focus to shift towards complementary subsets of neighbors.

Algorithmic Overview

The following algorithm summarizes the computation of a single GAT layer, highlighting the key steps of feature projection, attention scoring, normalization, and neighborhood aggregation.

Algorithm 1 Computation of a GAT Layer

Require:
The input node features set as $\mathbf{h} = \{\mathbf{h}_1, \mathbf{h}_2, \ldots, \mathbf{h}_N\}$, $\mathbf{h}_i \in \mathbb{R}^F$
A graph structure defining neighborhoods $\mathcal{N}_i$ for each node i
Ensure:
Node features updated as, $\mathbf{h}' = \{\mathbf{h}'_1, \mathbf{h}'_2, \ldots, \mathbf{h}'_N\}$, $\mathbf{h}'_i \in \mathbb{R}^{F'}$
Model Parameters:
weight matrix $\mathbf{W} \in \mathbb{R}^{F' \times F}$
Attention mechanism parameters $\mathbf{a}$
for all node i **do**
  Project input features: $\mathbf{z}_i \leftarrow \mathbf{W}\mathbf{h}_i$
end for
for all node i **do**
  for all neighbor $j \in \mathcal{N}_i$ **do**
    Compute attention score: $e_{ij} \leftarrow \text{LeakyReLU}(\mathbf{a}^\top [\mathbf{z}_i \| \mathbf{z}_j])$
  end for
end for
for all node i **do**
  Normalize attention scores: $\alpha_{ij} \leftarrow \frac{\exp(e_{ij})}{\sum_{k \in \mathcal{N}_i} \exp(e_{ik})}$
  Aggregate features: $\mathbf{h}'_i \leftarrow \sigma\left(\sum_{j \in \mathcal{N}_i} \alpha_{ij} \mathbf{z}_j\right)$
end for
return $\mathbf{h}'$

This formulation allows each node to selectively attend to the most informative neighbors, enhancing representation learning for graph-structured data. Multi-head attention further improves feature richness and stability, making GATs particularly effective for tasks like single-cell transcriptomic data integration, where local neighborhood structures encode important biological signals.

3.2 Generative Adversarial Network (GAN)

In order to facilitate the generation of prime-quality scRNA-seq data, we build on the framework of Generative Adversarial Networks (GANs), originally introduced by Goodfellow et al. GANs constitute a kind of deep generative model crafted to master complex, high-dimensional, and low-frequency data distributions, making them particularly well-suited for single-cell transcriptomics. By producing realistic synthetic data, GANs help overcome several critical challenges in bioinformatics, including limited sample sizes, high experimental costs, and privacy concerns, while enabling robust computational analysis and benchmarking of downstream methods.

A Generative Adversarial Network (GAN) involves two competing neural networks: a **Generator** (G) and a **Discriminator** (D). These networks participate in a competitive process similar to a zero-sum game. The generator is tasked to produce samples of data that resemble real scRNA-seq data, beginning with a basic noise vector z sampled from a latent space. The discriminator functions as a binary classifier, differentiating between actual data points and the synthetic samples produced by G. During training, the generator attempts to outmaneuver the discriminator, while the discriminator consistently hones its skill to recognize counterfeit samples. This competitive process iterates until an equilibrium is reached, at which point the generator produces synthetic cells that closely mimic the true distribution, both statistically and biologically.

From a statistical perspective, generative models aim to approximate the probability distribution $p_{\text{data}}(x)$ representing the training data to produce new, realistic samples. Traditional approaches, such as Hidden Markov Models or Gaussian Mixture Models, are *explicit* generative models: they define a mathematical form for the probability density function and learn its parameters, usually by maximizing the likelihood of the observed data. However, for high-dimensional, sparse, and complex data like scRNA-seq, these methods often become intractable.

GANs take a fundamentally different approach as *implicit* generative models. Instead of explicitly defining a probability distribution, the generator learns a mapping from a simple latent variable $z \sim p_z(z)$ to the complex data distribution $p_g(x)$. The discriminator guides this learning by evaluating

how well the generated data matches the real data distribution. The optimization objective of a GAN can be expressed as a min-max problem:

$$\min_G \max_D V(D,G) = \mathbb{E}_{x \sim p_{\text{data}}(x)}[\log D(x)] + \mathbb{E}_{z \sim p_z(z)}[\log(1 - D(G(z)))] \quad (7)$$

This configuration is mathematically equivalent to optimizing the Jensen-Shannon divergence between the distribution generated by the generator and the real data distribution, with a solid statistical foundation for adversarial training.

In comparison with other generative methods such as Variational Autoencoders (VAEs), Restricted Boltzmann Machines (RBMs), Deep Belief Networks (DBNs), or denoising autoencoders, GANs have some key benefits for scRNA-seq data. They are able to capture sparse and multimodal distributions without access to the explicit probability function, produce realistic samples with rich biological detail, and generate new cells at low cost in a single forward pass through the generator.

However, one of the big problems of the traditional GANs is suffering from training instability and mode collapse, which can restrict diversity. To mitigate this, we introduce a new GAN architecture developed specifically for single-cell data. Our architecture maintains cell-type heterogeneity, preserves gene-gene relationships, and stabilizes training so that synthetic data closely resembles real scRNA-seq datasets with biological fidelity for downstream analysis and validation of the model.

3.3 Comparative positioning of GARAGE with related GAN variants

To clarify the architectural novelty of GARAGE, we compared it with representative GAN-based generative frameworks, including standard GAN, cGAN, AC-GAN, WGAN/f-GAN, and LSH-GAN. This is demonstrated in the table 1. The comparison focuses on the generator input, conditioning or guidance mechanism, supervision type, discriminator objective, and treatment of rare cell populations. Unlike conventional conditioning-based GANs, GARAGE does not rely only on class labels or auxiliary classifier supervision. Instead, it constructs a cell–cell graph, learns attention-based node priorities, and injects selected high-attention real-cell embeddings into the generator input through a leakage-controlled hybrid input. This makes the generator input graph-informed and rare-cell-aware rather than purely noise-driven or label-conditioned.

4 Supplementary Note 3: GARAGE Model Parameters

4.1 Supplementary Note 3.1: GAT Model and Training Parameters:

The Graph Attention Network (GAT) in GARAGE identifies a core set of biologically representative cells to guide the generative process. The implementation and training of this module were configured with the following parameters.

(i) First, a kNN algorithm was used for constructing **a cell-cell neighborhood graph (KNN Graph)** from the input data matrix. Each cell was connected to its **5 nearest neighbors** (n_neighbors=5).

(ii) The GAT model built upon this graph is a two-layer architecture.

(iii) The initial GATConv layer employs a **multi-headed attention strategy featuring 8 attention heads** to capture diverse relational patterns, transforming the input gene features into **a 32-dimensional embedding space per head.**

(iv) Following a ReLU activation function, a subsequent GATConv layer with a single attention head aggregates these features to produce the final predictions for cell-type classification.

(v) During training, we also incorporated a mechanism to manually boost the importance of pre-selected rare cell types (index_list).

(vi) applying a **priority_weight of 2** to their intermediate representations to ensure they were given special consideration by the network.

(vii) The GAT model underwent training for a total of 7501 epochs to achieve thorough convergence.

Table 1: Conceptual and architectural comparison of GARAGE with related GAN variants.

**Method**	**Generator input**	**Conditioning / guidance signal**	**Supervision type**	**Discriminator objective**	**Rare-cell treatment / distinction from GARAGE**
Standard GAN	Random noise vector z	None	Unsupervised adversarial learning	Real/fake discrimination	No explicit mechanism for rare-cell preservation; minority modes may be dropped during adversarial training. GARAGE modifies the generator input using graph-attentive real-cell anchors.
cGAN	Noise vector z with class label or condition y	External class label or known covariate	Label-conditioned adversarial learning	Real/fake discrimination conditioned on y	Depends on reliable external labels and does not itself identify rare-cell-enriched anchors. GARAGE learns cell priorities from the cell–cell graph rather than using only a label code.
AC-GAN	Noise vector z, usually with class information	Class-label information through an auxiliary classifier	Supervised or semi-supervised class-aware generation	Real/fake discrimination plus auxiliary class prediction	Encourages class-discriminative generation but does not directly solve rare-cell under-sampling. GARAGE does not add an auxiliary classifier head; instead, rare-cell guidance is introduced through GAT-selected leakage into the generator input.
WGAN / f-GAN	Random noise vector z	None, but uses alternative adversarial divergence/objective	Unsupervised adversarial learning	Wasserstein distance or f-divergence-based adversarial objective	Improves stability of adversarial training but does not explicitly preserve rare cell states. GARAGE changes the generator input distribution using graph-attentive seed leakage rather than only changing the adversarial loss.
LSH-GAN	Noise vector with LSH-guided training strategy	Locality-sensitive hashing-based similarity structure	Unsupervised or data-guided adversarial learning	GAN-based real/fake discrimination	Uses hashing-based similarity to guide generation from limited samples, but does not learn attention scores over a cell–cell graph. GARAGE uses GAT-derived attention to select biologically informative and rare-cell-enriched seed cells.
GARAGE	Hybrid input $\mathbf{Z}'$ containing GAT-selected seed cells and random noise vectors	GAT-selected high-priority real-cell anchors from a kNN cell graph	GAT-guided seed selection followed by adversarial generation	Real/fake discrimination with graph-attentive hybrid generator input	Explicitly prioritizes biologically informative and rare-cell-relevant regions of the scRNA-seq manifold through attention-guided leakage. This is distinct from standard noise input, label conditioning, auxiliary classification, or hashing-based guidance.

Table 2: Reproducibility summary of the GARAGE architecture and training configuration used in the reported experiments.

**Component / hyper-parameter**	**Setting used in GARAGE experiments**
Input data	Log-normalized scRNA-seq expression matrix $\mathbf{X} \in \mathbb{R}^{N\times M}$ after quality control and preprocessing.
Cell graph construction	k-nearest-neighbour graph constructed in gene-expression space with $k_{\text{nn}} = 5$.
GAT architecture	Two-layer GATConv network. The first GATConv layer used 8 attention heads with 32 hidden units per head, followed by ReLU activation. The second GATConv layer used a single attention head to produce cell-type predictions.
GAT training objective	Supervised cell-type prediction using cross-entropy loss.
GAT optimizer and learning rate	Adam optimizer with learning rate 0.005.
GAT training epochs	7501 epochs.
Rare-cell priority weighting	A priority weight of 2 was applied to preselected rare-cell-type representations during GAT training.
Attention score aggregation	Incoming attention coefficients were aggregated as $S(c_i) = \sum_{j\in\mathcal{N}_i} \alpha_{ji}$. Cells were ranked by $S(c_i)$ to form the GAT-prioritized seed set $\mathbf{X}_{\text{new}}$.
Seed set size	For a leakage setting λ, the number of selected seed cells was $K = \lfloor \lambda N \rfloor$, unless otherwise stated.
Hybrid input form	Batch-level mixture of GAT-prioritized seed cells and random noise vectors: $\mathbf{Z}' = \{\mathbf{x}^{(1)}_{\text{seed}}, \ldots, \mathbf{x}^{(m_{\text{seed}})}_{\text{seed}}\} \cup \{\mathbf{z}^{(1)}, \ldots, \mathbf{z}^{(m_{\text{noise}})}\}$, where $m_{\text{seed}} = \lfloor \lambda m \rfloor$ and $m_{\text{noise}} = m - m_{\text{seed}}$. The union denotes batch-wise concatenation followed by shuffling.
Use of γ	The γ parameter associated with attention-pooled seed-vector construction was not used in the reported experiments. The reported implementation used discrete GAT-selected seed-cell leakage.
Generator architecture	Multilayer perceptron with two hidden layers: $[512, 512]$. Hidden layers used LeakyReLU activation with negative slope 0.2. The output layer was linear to generate real-valued log-normalized gene-expression profiles.
Discriminator architecture	Multilayer perceptron with hidden layers $[256, 128]$, using LeakyReLU activation with negative slope 0.2. An intermediate layer mapped learned features back to the input dimension before final real/fake classification.
Generator learning rate	0.0002.
Discriminator learning rate	0.0004.
GAN training iterations	20001 iterations.
Discriminator / generator update steps	$n_d = 5$ discriminator updates and $n_g = 2$ generator updates per iteration.
Label smoothing	One-sided label smoothing was used: real labels = 0.9 and fake labels = 0.1.
Gradient clipping	Discriminator gradient clipping with maximum norm 1.0.
Batch size	Full-batch training was used; therefore, $m = N$ for each dataset.
Leakage rates	$\lambda \in \{0.10, 0.20, 0.30\}$ were evaluated for GARAGE. $\lambda = 0$ corresponds to the conventional GAN/no-leakage control.

Table 3: Dataset-specific GARAGE training settings. Full-batch training was used, so the batch size m equals the number of cells N for each dataset.

**Dataset**	**Cells** (N)	**Genes** (M)	**Batch size** (m)	k_{nn}	**Leakage rates** λ	γ
Yan	90	10564	90	5	0.10, 0.20, 0.30	Not used
Pollen	299	9665	299	5	0.10, 0.20, 0.30	Not used
CBMC	7895	2000	7895	5	0.10, 0.20, 0.30	Not used
Muraro	2126	19127	2126	5	0.10, 0.20, 0.30	Not used

(viii) The training procedure employed the **Adam optimizer** with a **learning rate set at 0.005** and utilized the **Cross-Entropy Loss function**, which is commonly used for multi-class classification problems.

(ix) After training, the learned attention coefficients from the final layer were aggregated for each cell to compute an "importance score," quantifying its influence on its neighbors. The cells were then ranked by this score, and the top-k cells were selected as the high-priority seed set (Xnew) for the subsequent GAN stage.

Dynamic Neighboring Integration using Attention Mechanism

GAT estimates the relative importance of neighbors through a learnable attention kernel and then aggregates neighbor features with those data-dependent weights. We summarize the computation in four phases (cf. Velicković et al. [6]), extending each phase with options that are useful for sparse, high-dimensional scRNA-seq graphs.

Projection of features Each node (cell) i with input feature $\mathbf{x}_i \in \mathbb{R}^F$ is mapped to a head-specific latent:

$$\mathbf{z}_i \;=\; \mathbf{W}\,\mathbf{x}_i \;\in\; \mathbb{R}^d, \qquad \mathbf{W} \in \mathbb{R}^{d\times F}. \tag{8}$$

Optionally apply feature dropout on $\mathbf{x}_i$ and/or layer normalization on $\mathbf{z}_i$ to improve stability in HDSS regimes. Self-loops are added by setting $i \in \mathcal{N}_i$.

Pairwise relevance scoring For each edge (i, j), an attention MLP produces an unnormalized affinity score. A standard, efficient form is:

$$s_{ij} \;=\; \text{LeakyReLU}\Big(\boldsymbol{\theta}^\top \big[\mathbf{z}_i \oplus \mathbf{z}_j\big] \;+\; b\Big), \qquad \boldsymbol{\theta} \in \mathbb{R}^{2d}, \tag{9}$$

where $\oplus$ denotes concatenation and the negative-slope of LeakyReLU is typically 0.2. If a prior edge affinity κ_{ij} (e.g., cosine similarity in a PCA space) is available, it can be injected as a soft bias:

$$s_{ij} \;\leftarrow\; s_{ij} \;+\; \delta\,\kappa_{ij}, \qquad \delta \geq 0, \tag{10}$$

which helps denoise spurious edges without overriding learned attention.

Stochastic neighborhood focusing Scores are normalized over the (masked) neighborhood via a temperature-scaled softmax:

$$\omega_{ij} \;=\; \frac{\exp\big(s_{ij}/\tau\big)}{\sum\limits_{k\in\mathcal{N}_i} \exp\big(s_{ik}/\tau\big)}, \qquad \sum_{j\in\mathcal{N}_i} \omega_{ij} = 1, \;\; \tau > 0, \tag{11}$$

where smaller τ sharpens focus (useful for rare-cell neighborhoods) and larger τ smooths across noisy neighbors. To regularize, apply dropout on attention and (optionally) prune to the top-t neighbors:

$$\tilde{\omega}_{ij} \;=\; \text{Dropout}_{p_{\text{att}}}\big(\omega_{ij}\big), \tag{12}$$

$$\tilde{\omega}_{ij} \;=\; \frac{\mathbf{1}\{j \in \text{Top-}t(\omega_{i:})\}\,\omega_{ij}}{\sum\limits_{k\in\text{Top-}t(\omega_{i:})} \omega_{ik}} \quad \text{(optional top-}t\text{ renormalization).} \tag{13}$$

Context-aware representation Neighbor messages are aggregated with attention weights; an output projection and residual pathway stabilize training and preserve cell-specific signal:

$$\mathbf{m}_i = \sum_{j \in \mathcal{N}_i \cup \{i\}} \tilde{\omega}_{ij}\, \mathbf{z}_j, \tag{14}$$

$$\mathbf{z}'_i = \text{LayerNorm}\Big(\phi\big(\mathbf{W}_o\, \mathbf{m}_i + \mathbf{b}_o\big) + \mathbf{R}\, \mathbf{z}_i\Big). \tag{15}$$

Here, ϕ is typically ELU; $\mathbf{W}_o, \mathbf{b}_o$ are output parameters; and $\mathbf{R}$ is either the identity (residual) or a lightweight gate (e.g., $\mathbf{R} = \text{Diag}(\boldsymbol{\sigma})$ with $\boldsymbol{\sigma} = \text{sigmoid}(\mathbf{U}\mathbf{z}_i)$). Feature dropout on $\mathbf{z}_j$ can be incorporated before (14).

Multi-Perspective Attention

To capture heterogeneous cell–cell relations, K independent heads run in parallel. For head k:

$$s_{ij}^{(k)} = \text{LeakyReLU}\Big((\boldsymbol{\theta}^{(k)})^\top \big[\mathbf{W}^{(k)}\mathbf{x}_i \oplus \mathbf{W}^{(k)}\mathbf{x}_j\big] + b^{(k)}\Big), \tag{16}$$

$$\omega_{ij}^{(k)} = \frac{\exp\big(s_{ij}^{(k)}/\tau\big)}{\sum_{u \in \mathcal{N}_i} \exp\big(s_{iu}^{(k)}/\tau\big)}, \qquad \tilde{\omega}_{ij}^{(k)} = \text{Dropout}_{p_{\text{att}}}\big(\omega_{ij}^{(k)}\big), \tag{17}$$

$$\mathbf{m}_i^{(k)} = \sum_{j \in \mathcal{N}_i \cup \{i\}} \tilde{\omega}_{ij}^{(k)}\, \mathbf{W}^{(k)}\mathbf{x}_j. \tag{18}$$

Intermediate layers concatenate head outputs to increase capacity:

$$\mathbf{z}'_i = \big\|_{k=1}^{K} \phi\big(\mathbf{W}_o^{(k)}\mathbf{m}_i^{(k)} + \mathbf{b}_o^{(k)}\big), \tag{19}$$

whereas the final layer typically averages to preserve dimensionality and improve stability:

$$\mathbf{z}'_i = \phi\left(\frac{1}{K}\sum_{k=1}^{K} \mathbf{W}_o^{(k)}\mathbf{m}_i^{(k)} + \mathbf{b}_o^{(k)}\right). \tag{20}$$

A modest temperature $\tau < 1$ sharpens heads around rare-cell neighborhoods; $\tau > 1$ makes heads more tolerant to noise. Head-wise dropout and weight decay on $\{\mathbf{W}^{(k)}, \boldsymbol{\theta}^{(k)}\}$ mitigate overfitting.

From attention to priority nodes To derive node salience used by *GARAGE*, accumulate incoming attention across layers/heads:

$$s_i = \frac{1}{LK}\sum_{\ell=1}^{L}\sum_{k=1}^{K}\sum_{j \in \mathcal{N}_i} \alpha_{ji}^{(\ell,k)}, \qquad \pi_i = s_i \cdot r_i, \tag{21}$$

where $\alpha_{ji}^{(\ell,k)}$ are the normalized attentions at layer ℓ, head k, and r_i is an optional rarity prior (e.g., inverse local density). The top-q% by π_i define the *priority nodes* that seed the GARAGE generator.

4.2 Supplementary Note 3.2: GAN Model and Training Parameters:

The adversarial generation (Goodfellow et al. [7]) module of GARAGE consists of a Generator and a Discriminator, both implemented as Multi-Layer Perceptrons (MLPs), which are trained in a competitive min-max game to produce high-fidelity synthetic cell samples.

(i) The Generator network is designed to map the hybrid input batch (composed of GAT-selected seed cells and random noise) to the high-dimensional gene expression space. Its architecture consists of two hidden layers, each with 512 neurons (hsize_G = [512, 512]), providing substantial capacity for learning complex gene-gene correlations.

(ii) LeakyReLU activation function with a negative slope set to 0.2, was used in the hidden layers to counter the issue of vanishing gradient. With the final output layer having no activation function, the network was allowed to produce unconstrained real-valued gene expression profiles suitable for log-normalized data.

(iii) Conversely, the Discriminator network is intentionally designed with a smaller capacity to help stabilize training and prevent it from overpowering the generator. It is structured with two primary hidden layers of 256 and 128 neurons, respectively (hsize_D = [256, 128]), also using LeakyReLU activations.

(iv) The **Discriminator** is made smaller for the purpose of stabilizing training and comprises two hidden layers with 256 and 128 neurons (hsize_D = [256, 128]) and likewise utilizing LeakyReLU activations.

(v) An intermediate layer is another notable aspect of the discriminator that maps the learned features back to the dimension of the original input before the final output of classification.

(vi) To ensure the discriminator learns effectively and provides a robust gradient signal to the generator, the discriminator was used with a higher learning rate (lr_d = 0.0004) compared to the generator (lr_g = 0.0002). This configuration encourages a balanced and stable adversarial dynamic throughout the training process.

4.3 Supplementary Note 3.3: GARAGE Model Training Parameters:

The competitive training of the GARAGE model is conducted over 20,001 iterations to ensure convergence. Each iteration consists of two main phases: training the discriminator, followed by training the generator. The entire training process operates on a full-batch basis, where the complete real dataset and a corresponding full-sized synthetic batch are processed in each step.

(i) Real Data Batch (X_batch): This batch consists of the entire preprocessed real scRNA-seq dataset (x_plot). It serves as the ground truth distribution, replicating which is the goal of the generator.

(ii) The discriminator is trained for a set number of steps (nd_steps = 5) for every generator update, a common practice to keep the discriminator slightly ahead in the adversarial game.

(iii) To stabilize training and prevent the discriminator from becoming overconfident, we employ one-sided label smoothing. Instead of using hard labels of 1 for real and 0 for fake, soft labels were used (real: 0.9 and fake: 0.1). The discriminator's total loss (disc_loss) is the aggregation of the Binary Cross-Entropy losses for the real and fake batches against these soft labels.

(iv) The discriminator's weights are updated by backpropagating the disc_loss. To to improve stability and reduce the risk of exploding gradients, a gradient clipping was employed with a maximum norm - 1.0 before the optimizer (disc_optimizer) takes a step.

(v) Following the discriminator's updates, the generator is trained for ng_steps = 2.

(vi) same training is applied for the generator step, as same as the discriminator.

4.4 Supplementary Note 3.4: GARAGE Model Wasserstein Distance (WD) Parameters

In our *GARAGE* pipeline, we generated the 6 different sizes of synthetic data, all have the same gene topology (feature set), but all the synthetic data differ in their sample size.

We employ 0.25 to 1.5 times with a step size of 0.25 of the actual sample size for the synthetic data. (Iteration 0 to 5 for sample sizes 0.25 to 1.5, respectively)

Below, we measured the Wasserstein distance (WD) between the best generated data distribution and the original data, and we mentioned the per-dataset specific synthetic data that minimized the WD in our GARAGE.

4.4.1 YAN Dataset

- 0% Leakage – Iteration 5, generated dataset from Traditional GAN
- 10% Leakage – Iteration 3, generated dataset from GARAGE
- 20% Leakage – Iteration 3, generated dataset from GARAGE
- 30% Leakage – Iteration 3, generated dataset from GARAGE

4.4.2 Pollen Dataset

- 0% Leakage – Iteration 5, generated dataset from Traditional GAN
- 10% Leakage – Iteration 5, generated dataset from GARAGE
- 20% Leakage – Iteration 3, generated dataset from GARAGE
- 30% Leakage – Iteration 5, generated dataset from GARAGE

4.4.3 CBMC Dataset

- 0% Leakage – Iteration 3, generated dataset from Traditional GAN
- 10% Leakage – Iteration 5, generated dataset from GARAGE
- 20% Leakage – Iteration 5, generated dataset from GARAGE
- 30% Leakage – Iteration 5, generated dataset from GARAGE

4.4.4 MURARO Dataset

- 0% Leakage – Iteration 1, generated dataset from Traditional GAN
- 10% Leakage – Iteration 5, generated dataset from GARAGE
- 20% Leakage – Iteration 5, generated dataset from GARAGE
- 30% Leakage – Iteration 5, generated dataset from GARAGE

4.5 Supplementary Note 3.5: Benchmarking state-of-the-art (SOTA) models parameters

For the synthetic data generation, all the SOTA models generated data for all the four scRNA-seq data. Here, for all the SOTA models, architecture building and the synthetic data generation pipeline, used Python (v3.7.12) and Tensorflow (v2.11.0).

Below, we are stating the parameters for the models -

4.5.1 GAN

- latent dimension used 100 & batch size as 64.
- The generator is structured with a three-layer architecture with 32 neurons in the hidden layer and utilizing the tanh activation function.
- The discriminator is structured with 3 layer architecture with 32 neurons in the hidden layer and a ReLU activation function.
- Finally, a linear layer that need to distinguish the sample between fake and real.
- Used sigmoid cross entropy loss and Adam optimizer with 0.0001 learning rate.

4.5.2 fGAN

- latent dimension used 100, batch size as 64, number of critics is 5 & used constraint penalty weight as 10 (default)
- At the generator, the hidden layer was constructed with a 3-layer architecture with 128 neurons and LeakyReLU activation.
- At the discriminator, a 3-layer architecture with 128 neurons and LeakyReLU activation at the hidden layer was built.
- Finally, a linear layer that need to distinguish the sample between fake and real.
- Used Adam optimizer with $5e-4$ learning rate.

4.5.3 wGAN

- latent dimension used 100, batch size as 64, number of critic is 5 & used clip value (weight clipping) as 0.1 (default)
- The generator was built with 3 layer architecture with the hidden layer having 32 neurons and tanh activation.
- The discriminator was built with 3 layer architecture with the hidden layer having 32 neurons and tanh activation.
- Finally, a linear layer that need to distinguish the sample between fake and real.
- Used RMSProp optimizer with 0.0005 learning rate.

4.5.4 LSH-GAN

- Used the actual default parameters from the paper of Lall et al. [8].
- Found the nearest neighbors using LSH with n_estimators = 10.
- At the generator, 4 4-layer architecture with 16 neurons each and LeakyReLU activation at each hidden layer.
- At the discriminator built 4 layer architecture with 16 neurons and LeakyReLU activation at each hidden layers.
- Finally, a linear layer that needs to distinguish the sample between fake and real.
- Used sigmoid cross-entropy loss and RMSProp optimizer with 0.001 learning rate.
- Here, the generators and discriminator run 10 times both internally in each epoch.

4.5.5 VAE

- latent dimension used 100, batch size as 64.
- At the generator, 3 3-layer architecture was built with the hidden layer having 128 neurons and LeakyReLU activation.
- At the discriminator built 3 layer architecture with 128 neurons and tanh activation at the hidden layer.
- Performed the VAE, reparameterization trick (sample from latent distribution)
- The discriminator was built with 3 layer architecture with the hidden layed having 32 neurons and tanh activation.
- Finally, a linear layer that need to distinguish the sample between fake and real.
- Used Adam optimizer with $5e-4$ learning rate.

5 Supplementary Note 4: GARAGE Model Results

As we discussed earlier, the GARAGE models have been made to discover the new form of science at the **single cell synthetic data generation**. We have performed our model to the 4 different datasets. On each dataset, we performed a conjugation approach where the synthetic data was generated not only by the random noise, but we also added k% (10, 20, and 30) leakage samples of real data with it. It, therefore enhance the generated data performance and also it bits in data quality and in metric the SOTA models.

Later to the data generation, we sampled the best features as the highly variable genes (HVGs) using PCA & Fano Index techniques and created 3 combination of data, as - Feature selection (FS) performed using real data and then applied to the real data, FS conducted on the generated data and subsequently applied to the real data, and lastly, FS executed on the combined dataset (both generated and real) before being applied to the real data.

Thus after this whole processing we would have having 3 combination of data, where we inject our validation technique to prove the difference in the broad scale at every dataset.

With this conjugation training approach we did the data generation and after feature selection done the data validation. Where by setting different adjusted neighbors and PCA component we performed the UMAP and performed 3 metrics as - ARI score, NMI and macro-f1 score. Here, ARI evaluates our clusters against the known ground truth or the known labels. NMI also tells us about the same story but it also consider about the shared context between the evaluated clusters and the ground truth information. Macro-f1 tells somewhat other story, as it describes the classification performance in the multi-class settings with the imbalance class distribution.

5.1 YAN Dataset

As discussed, in the supplementary - 2, the dimension of the yan dataset is (90, 10654). We choose the leakage samples as 10, 20 and 30 here.

As discussed earlier, there are 6 cell types for the entire YAN datasets. At the time of evaluating the generated data, we did the label encoded so the all 6 subtypes are labelled as 0 to 5. The reverse transformation are: 0 = 16cell; 1 = 2cell; 2 = 4cell; 3 = 8cell; 4 = blast; 5 = zygote

5.1.1 Feature Selection using Fano index

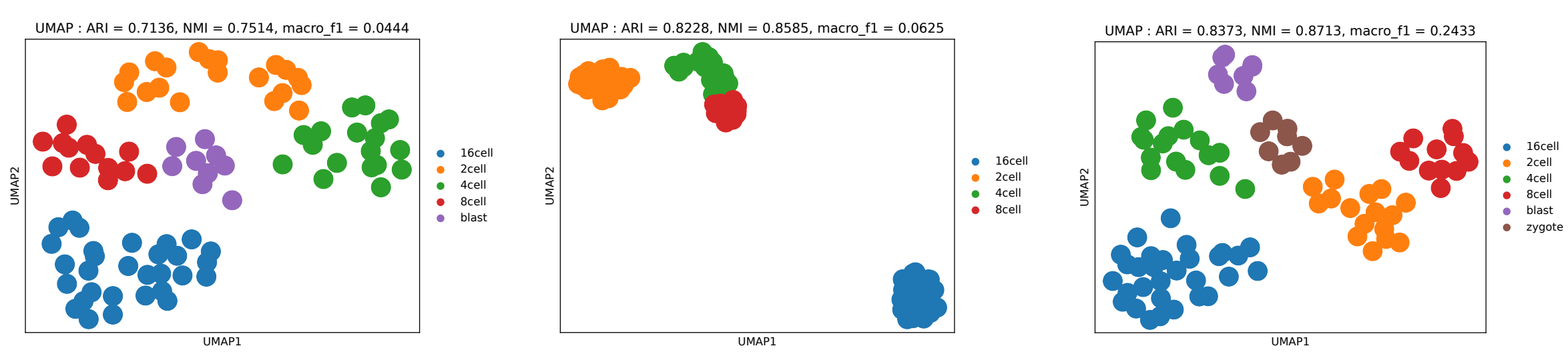

(a) YAN Real UMAP (b) YAN Generated UMAP (c) YAN Combined UMAP

Fig 1: YAN Dataset: k = 10 (10%)

These are the UMAP result with different metrics when the leakage is 10%. Here the real data umap will be same for all the cases but we can see the metrics improved, specifically the ARI and NMI, with the generated data to the combined data. For all 3 metrics the values closes to 1 means, it is perfect clusters and the larger f1 score defines that the class are predicted correctly irrespective of imbalance class distribution.

These are the UMAP result with different metrics when the leakage is 20%. Here, the results demonstrates the critical-dose effect of the GAT dependent real data leakage(k), that improve the generator ability to learn and replicate the complex pattern in the real scRNA-seq data. In the figure 1 the generator might experience a **mode-collapse** its learning is performed only at a subset

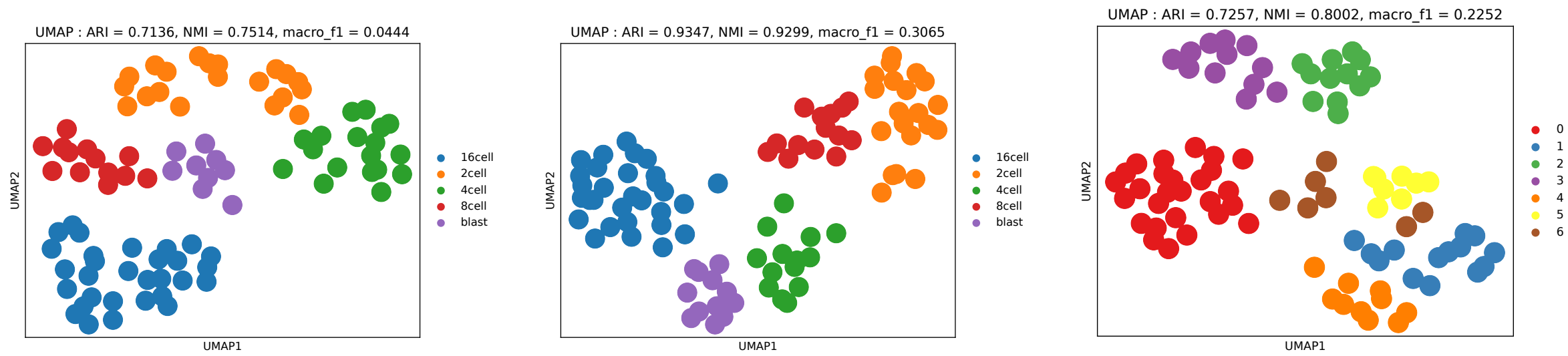

(a) YAN Real UMAP (b) YAN Generated UMAP (c) YAN Combined UMAP

Fig 2: YAN Dataset: k = 20 (20%)

of the real data. And, at higher data leakage (20%), the model might get the sufficient knowledge to overcome the mode-collapse and successfully learn the entire data manifold. Withe the sufficient real data knowlege at 20% the model become the high-performace generative-engine for the dataset.

The generator data cluster scores surpasses the real data ones, that depicts the model not only replicate the real scrna-seq data but also it learns the **central tendency of each cell types**.

Also, the combination scores are better than real but drastically lower than the generated data, which supports the denoising hypothesis. When clean generated data, mixed with noisy real data, the overall clustering efficiency dropped. The real data noise, prevents the data to achieve the near-perfect score.

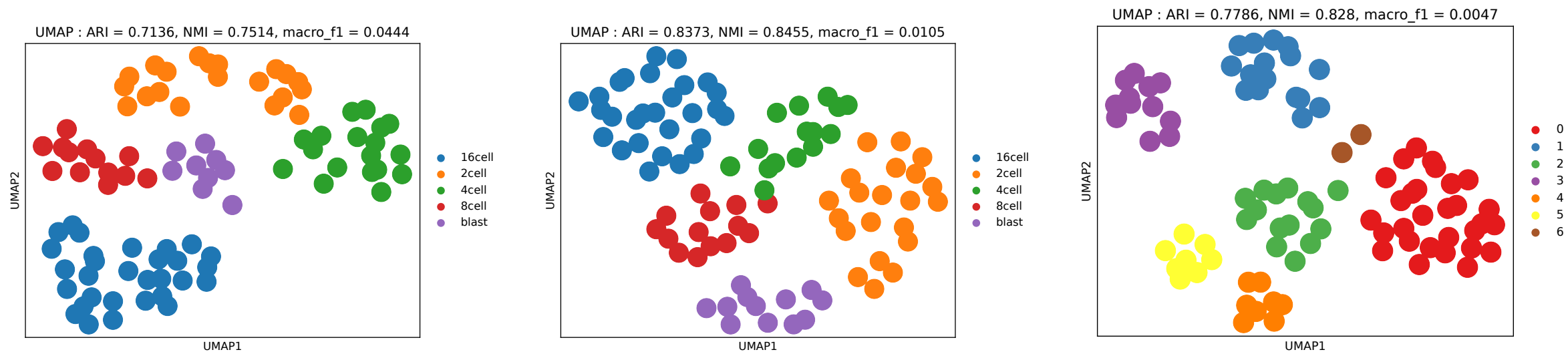

(a) YAN Real UMAP (b) YAN Generated UMAP (c) YAN Combined UMAP

Fig 3: YAN Dataset: k = 30 (30%)

These are the UMAP result with different metrics when the leakage is 30%. Here we can see the performance is degradated than the k = 20% data leakage, it depicts the excessive leakage (in this case) can force the GAN into a state of **overfitting or mode biasing**, where the generator tries to replicate the leakage sample too closely to make the data generation more generalizable. Also the poor f1-score describes about the bad cell type classification.

Thus, for the synthetic data generation of YAN dataset the k = 20% is the **sweet spot** that correctly describe the data generation.

Parameters: For generated data, at 10% leakage, utilized n_neighbors, n_pcs, resolution as (20, 18, 1.22) respectively; similarly for the 20% leakage, the gen data utilized (20, 19, 1.0), and for 30%, gen data utilized (20, 22, 1.0) respectively. (for all iter-3 gen data)

Now for comb data, the (n_neighbors, n_pcs, resolution) are (20, 20, 0.8); (20, 20, 0.8); (20, 18, 1.32) for 10%, 20%, and 30%, respectively. (for all iter-3 gen data)

The real data UMAP parameters are - (20, 20, 0.8) for n_neighbors, n_pcs, and resolution, respectively. (for all iter-3 gen data)

5.1.2 Feature Selection using CV2

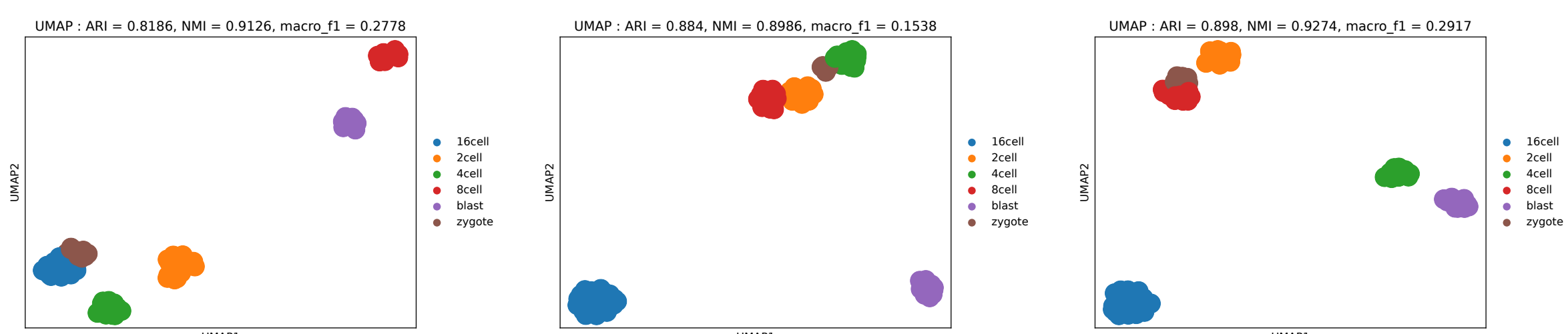

(a) YAN Real UMAP (b) YAN Generated UMAP (c) YAN Combined UMAP

Fig 4: YAN Dataset: k = 10 (10%)

Figure 4 contains the UMAP results for yan dataset at 10% leakage for fearures selected using CV2 method. The UMAP results for the real data, which remains same for each evaluation, show that the yan dataset shows a strong baseline performance even without any data leakage, achieving ARI = 0.8186 and NMI = 0.9126. This indicates that the inherent cell-type separability is high, and the embedding space already captures most of the structural variance between classes.

At 10% leakage, the measurement metrics show considerable improvement over the baseline for the generated data, with the UMAP displaying compact, well-organized clusters that maintain the underlying topology of the real data while slightly enhancing separation in certain regions. The combined dataset scores surpass both the real and generated alone, suggesting that at low leakage the generator is not only preserving subtype relationships but also providing a complementary denoising effect when merged with the original measurements.

When the leakage increases to 20%, the generated data achieves higher metric scores compared to baseline and lower leakage samples, indicating a more complete capture of the manifold structure as indicated in Figure 5. The combined data reaches its peak value here, with the UMAP plots

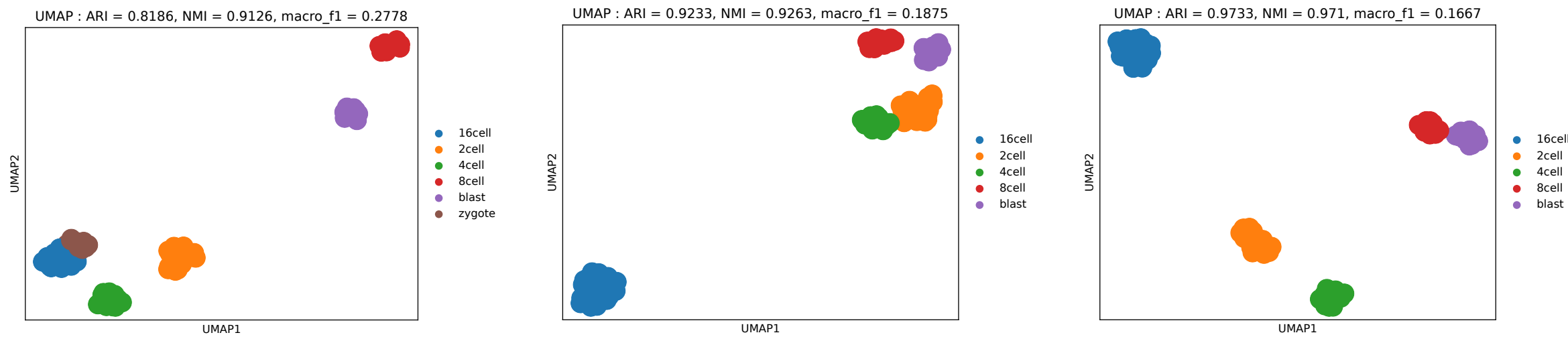

(a) YAN Real UMAP (b) YAN Generated UMAP (c) YAN Combined UMAP

Fig 5: YAN Dataset: k = 20 (20%)

showing that the synthetic and real samples are almost indistinguishable in the embedding space, with near-perfect cluster boundaries. This sharp gain in the combined scores suggests that the generator benefits from having enough real data exposure to eliminate residual variance and stabilize the cell-type centroids without overfitting.

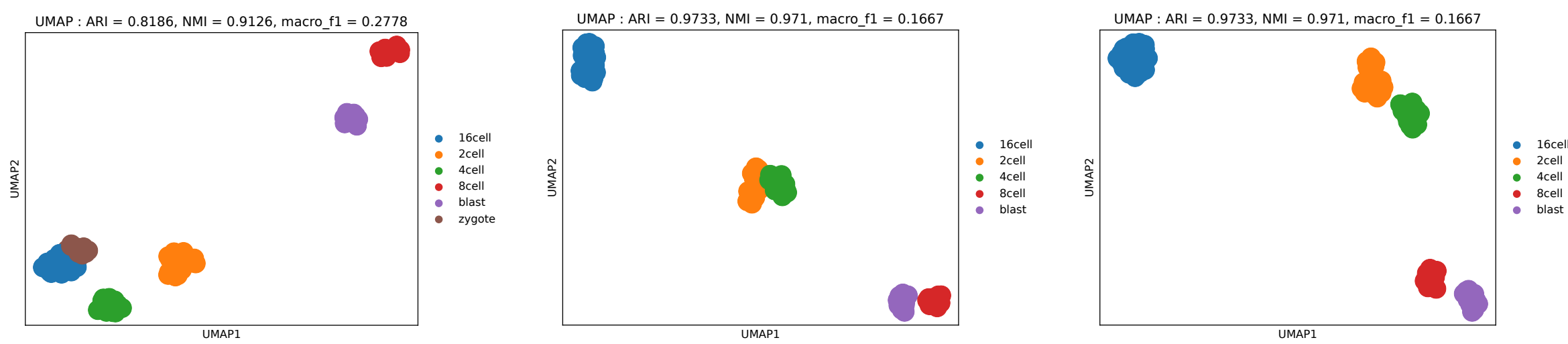

(a) YAN Real UMAP (b) YAN Generated UMAP (c) YAN Combined UMAP

Fig 6: YAN Dataset: k = 30 (30%)

Figure 6 contains the UMAP results for yan dataset at 30% leakage. A clear convergence of scores of the performance metrics for the generated data and combined data is observed. The UMAP shows distinct, neatly bounded clusters with a slight overlapping in case of synthesized data alone while the combined clusters are well-defined and readily distinguishable. However, the alignment between the two distributions, with almost complete intergration of synthetic data points into real measurements is quite noticeable. Although this is the maximum fidelity in terms of measurable, the fact that both 20% and 30% leakage cases have similar combined scores means that the model achieves its generative saturation point around 20% leakage for this data. After that, further leakage does not add measurable value and might actually reinforce previously learned associations.

Parameters: For generated data, at 10% leakage, the parameters (n_neighbors, n_pcs, resolution) are (20, 20, 1.55); for 20% leakage, they are (20, 20, 1.0); and for 30% leakage, they are (20, 20, 0.93), respectively. Now for combined data, the (n_neighbors, n_pcs, resolution) are (20, 17, 1.21); (20, 20, 1.0); and (20, 20, 1.0) for 10%, 20%, and 30% leakage, respectively. For real data, the (n_neighbors, n_pcs, resolution) are (20, 5, 1.2) for each leakage level.

5.2 POLLEN Dataset

The pollen dataset, comprising for 11 distinct cell types, was label encoded to facilitate clustering evaluation and the encoded cell types stood at: 0 = 2338, 1 = 2339, 2 = BJ, 3 = GW16, 4 = GW21, 5 = GW21+3, 6 = HL60, 7 = K562, 8 = Kera, 9 = NPC, and 10 = iPS. We proceeded with leakage samples at 10, 20 and 30 here.

5.2.1 Feature Selection using Fano index

Figure 7 shows the UMAP results for 10% leakage. The real data UMAP remains constant across all experiments, serving as baseline reference for visual and metric comparison. Here, both ARI and NMI for generated data are significantly larger than the real data baseline at this leakage level, and even the

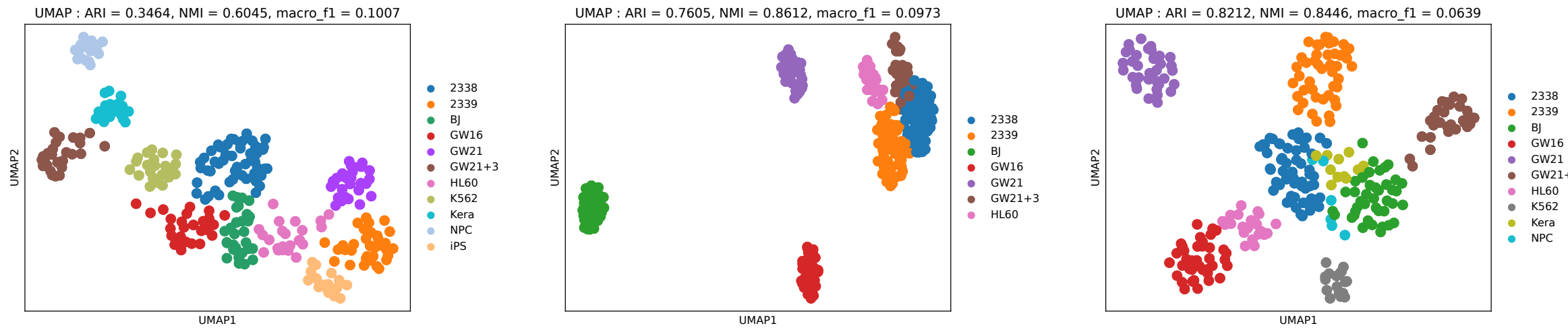

(a) POLLEN Real UMAP (b) POLLEN Generated UMAP (c) POLLEN Combined UMAP

Fig 7: POLLEN Dataset: k = 10 (10%)

combined data improves. The data already shows very good correspondence with the actual cell type distribution, and the joint UMAP shows a lot of overlap between real and synthetic points, implying that even a minimal leakage can assist the generator in approximating the underlying manifold well.

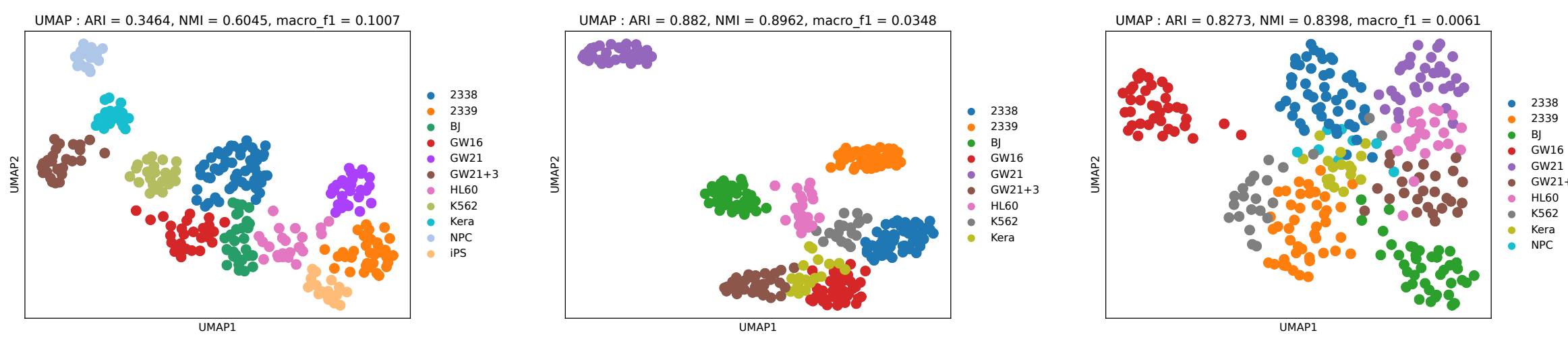

(a) POLLEN Real UMAP (b) POLLEN Generated UMAP (c) POLLEN Combined UMAP

Fig 8: POLLEN Dataset: k = 20 (20%)

Figure 8 shows the UMAP plots for the Pollen dataset with 20% leakage. The generator is performing its best at this level, where ARI and NMI reach their highest for the synthesized data. This means that the model has learned sufficient real data information to surpass possible partial learning effects observed at smaller leakage, allowing it to encode the complete complexity of the scRNA-seq manifold. Remarkably, though generated data scores are higher than those of the actual dataset, the overall scores are still slightly lower than the generated in isolation, which would indicate a possibilty that when combined with noisier actual data, the clustering evaluation leads to lower overall efficiency.

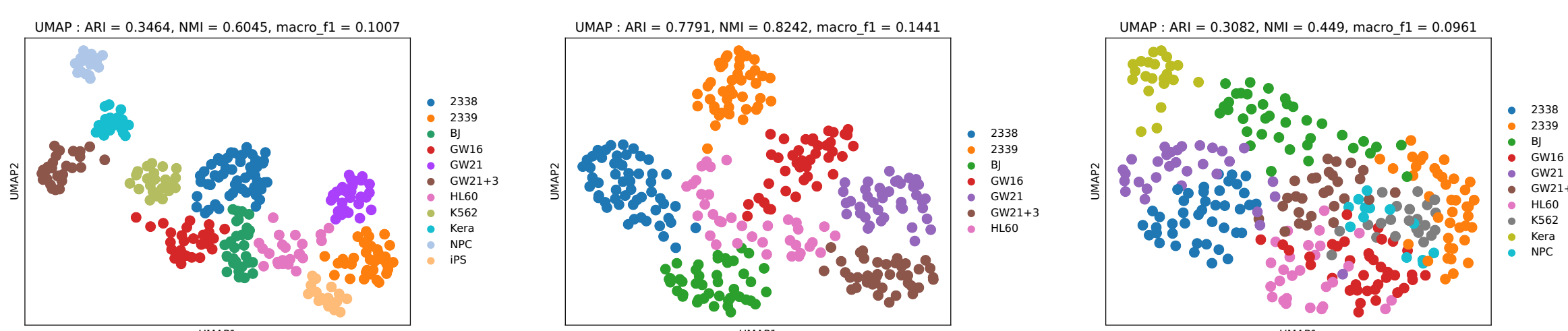

(a) POLLEN Real UMAP (b) POLLEN Generated UMAP (c) POLLEN Combined UMAP

Fig 9: POLLEN Dataset: k = 30 (30%)

At 30% leakage, as noticed in the UMAP results for the pollen dataset displayed in Figure 9, there is degradation in the metric scores for generated data, with the clusters losing compactness, and significant dispersion is observed at the edges. However, more notably, a peculiar and drastic drop is observed in the performance of the combined dataset. The UMAP plot highlights inconspicuous boundaries and concerning overlap between subtypes as the clusters lose definition and sharpness. This turnaround indicates that heavy real data leakage could overfit the generator to noisy features of the original set, diminishing the denoising benefit and deteriorating cluster separation. For the merged case, the degradation is even more significant, showing that interaction between highly real-like generated

data and true noisy points can destabilise the embedding and cause cluster fragmentation.

Overall, the Fano index-based feature selection trial with the Pollen dataset indicates 20% leakage as the optimal setting for high-fidelity synthetic data generation, yet 10% leakage as the optimal compromise in enhancing generated and combined datasets without causing noise-related instability. The performance at 30% leakage highlights the value of real data exposure control in maintaining the generator's denoising capacity and avoiding amplification of noise in integrated embeddings.

Parameters: For generated data, at 10% leakage, utilized n_neighbors, n_pcs, resolution as (30, 18, 1.5) respectively; similarly for the 20% leakage, the gen data utilized (30, 17, 2.01), and for 30%, gen data utilized (20, 17, 0.92) respectively. (for all iter-5 gen data)

Now for comb data, the (n_neighbors, n_pcs, resolution) are (30, 18, 1.8); (30, 30, 1.77); (30, 18, 1.51) for 10%, 20%, and 30%, respectively. (for all iter-5 gen data)

The real data UMAP parameters are - (30, 22, 1.16) for n_neighbors, n_pcs, and resolution, respectively. (for all iter-5 gen data)

5.2.2 Feature Selection using CV2

The Pollen dataset, evaluated after applying the coefficient of variation (CV2) based feature selection, reflects good baseline clustering quality in its actual data with ARI = 0.6891 and NMI = 0.8710. This confirms that the chosen features already retain good subtype separability among the cell population before any generation of synthetic data, which is a good starting point for generative modelling. The UMAP results for real data remains constant for each evaluation.

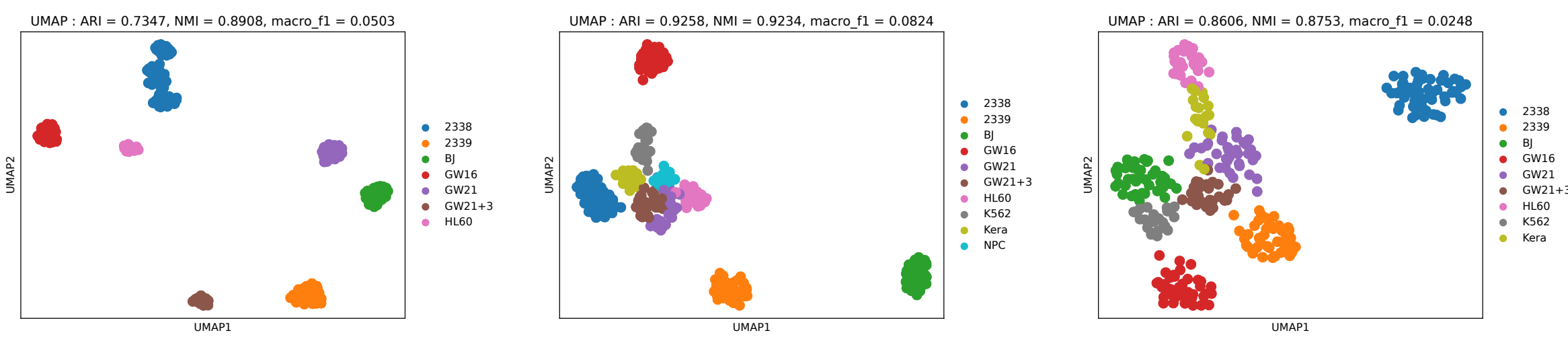

(a) POLLEN Real UMAP (b) POLLEN Generated UMAP (c) POLLEN Combined UMAP

Fig 10: POLLEN Dataset: k = 10 (10%)

The figure 10 displays the UMAP results for pollen dataset at 10% leakage, the generated data reaching its best performance in this test run. The UMAP projection contains well-separated, tightly packed clusters with synthetic points hugging the edges of the original subtypes. The score of the merged dataset, though still good, is ever so slightly lower than that of the synthetic-only data, indicating that the real data adds some variation that the synthetic data had removed. This emphasizes the fact that the generator, even when constrained by a small amount of true data, not only mimics the overall topology but also increases the local consistency of clusters, basically serving as a step of data refinement.

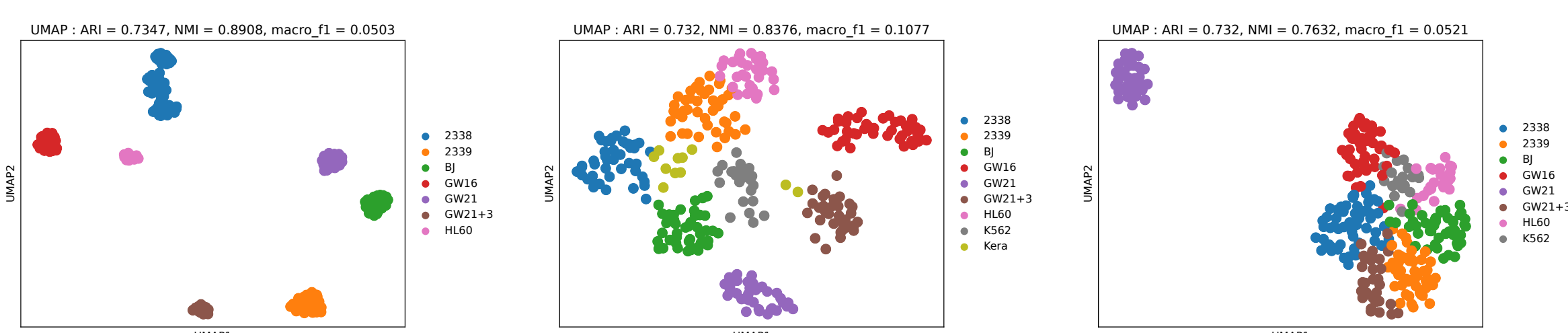

(a) POLLEN Real UMAP (b) POLLEN Generated UMAP (c) POLLEN Combined UMAP

Fig 11: POLLEN Dataset: k = 20 (20%)

With the leakage increasing to 20%, the UMAP plots for pollen dataset in figure 11 shows distinctively the significant decline in the performance metrics of the generated and combined samples.

Although higher metric values than baseline points of reference are still retained in real data, the sharpening of the clustering is below that of the previous leakage level. The UMAP plots indicate that although the clusters remain identifiable, they start to become less dense, with increased dispersion along cluster boundaries. This decrease indicates that the generator is likely overfitting to some local patterns at this leakage level and possibly losing some of the generalization advantage seen in the lower-leakage situation. The decrease in both NMI and ARI means that, within this feature-scaled region, too much intermediate leakage is not necessarily bolstering manifold learning and can upset the heretofore well-aligned structure.

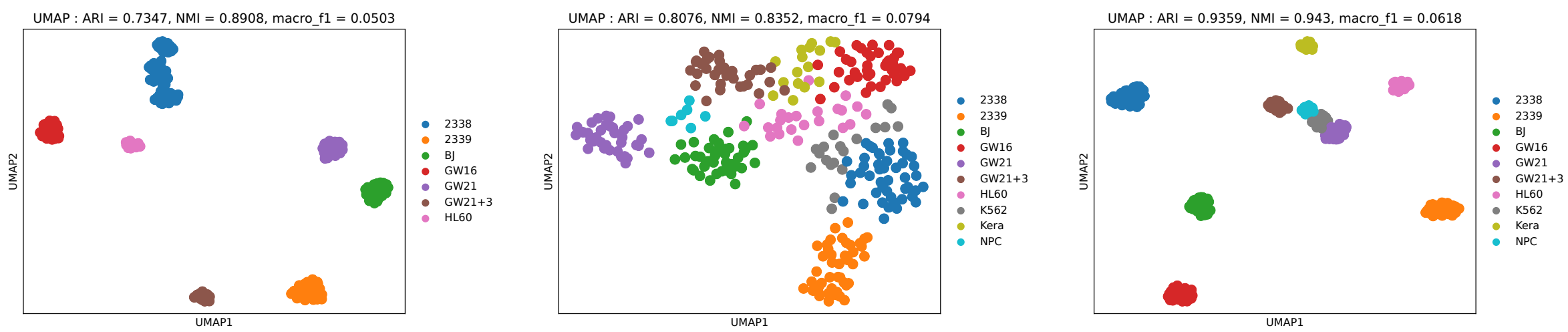

(a) POLLEN Real UMAP (b) POLLEN Generated UMAP (c) POLLEN Combined UMAP

Fig 12: POLLEN Dataset: k = 30 (30%)

A surprising improvement in shown in the clustering evaluation for generated data and the combined data reaches its highest performance scores at the leakage level of 30%, as illustrated in the UMAP results shown in Figure 12. The UMAP plots for synthesized data alone has comparatively distinct clusters as compared to 20% leakage but still displays dispersion at edges with no clear boundaries as in leakage at 10%. The combined samples show clear-cut, bounded and distinct clusters in this configuration, and indicates high overlap for subtypes between real and generated data in the combined space. This flip-over — where combined data outperforms generated-only — indicates that, at high levels of leakage, the generator generates data that is highly well-aligned with the real distribution, and the inclusion of real samples reinforces instead of destroying the cluster structure. In this scenario, generator and true data seem to complement each other best, leading to essentially perfect combined clustering measures.

Overall, the clustering analysis for CV2 feature selection indicates that the best leakage setting for synthetic-only performance is at the lower end (10%), but for overall combined dataset clustering efficiency, a higher leakage of 30% produces the optimal result. The contrast displays the role of generator acting as denoiser at low leakage point to eventually a manifold expander and structural stabilizer for higher leakage value.

Parameters: For generated data, the (n_neighbors, n_pcs, resolution) are (20, 22, 1.91); (20, 15, 1.25); and (20, 25, 1.80) for 10%, 20%, and 30% leakage, respectively. Now for combined data, the (n_neighbors, n_pcs, resolution) are (20, 25, 2.71); (20, 27, 1.44); and (20, 20, 1.95) for 10%, 20%, and 30% leakage, respectively. For real data, the (n_neighbors, n_pcs, resolution) are (30, 20, 3.76) for each leakage level.

5.3 CBMC Dataset

The CBMC dataset, with dimension (7895, 2000), contains a total of 13 annotated cell types, which underwent label encoding prior to clustering analysis, resulting in the following encoded labels: 0 = B, 1 = CD14+ Mono, 2 = CD16+ Mono, 3 = CD34+, 4 = CD8 T, 5 = DC, 6 = Eryth, 7 = Memory CD4 T, 8 = Mk, 9 = NK, 10 = Naive CD4 T, 11 = T/Mono doublets, and 12 = pDCs. With the leakage levels decided at 10, 20 and 30, we performed the evaluation.

5.3.1 Feature Selection using Fano index

Figure 13 contains the UMAP results with corresponding metrics for the cbmc dataset when the leakage is 10%. The original dataset yielded baseline clustering metrics of ARI = 0.1027 and NMI = 0.2082, indicating moderate subtypes separation within the embedding space. The UMAP for real data remains the same in each leakage evaluation. For 10% leakage, already the generated data shows a

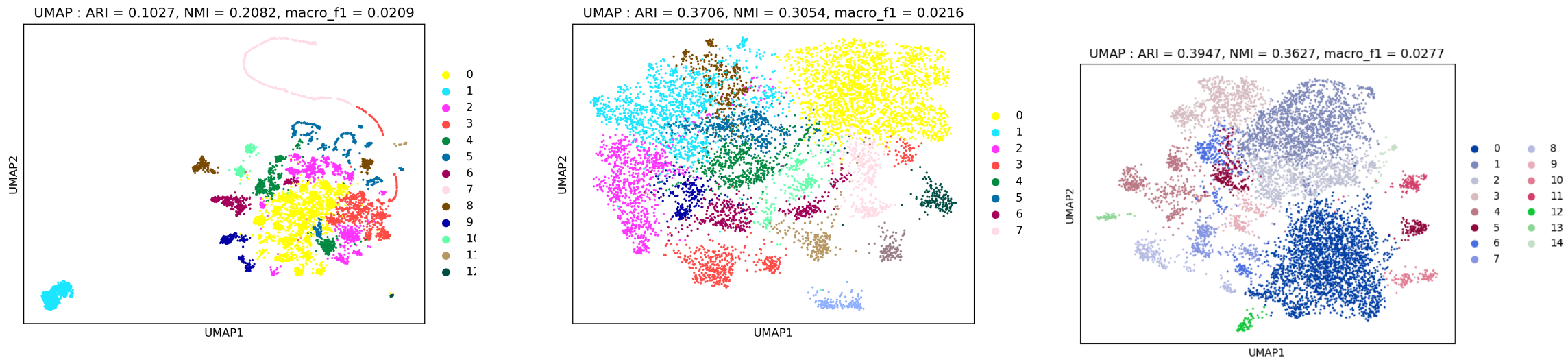

(a) CBMC Real UMAP (b) CBMC Generated UMAP (c) CBMC Combined UMAP

Fig 13: CBMC Dataset: k = 10 (10%)

significant boost in clustering quality with the UMAP creating better-defined clusters than the baseline but still with signification dispersion, and the combined data has higher overlap between synthetic and real cells. The neglible difference between generated and combined UMAP results demonstrates that at low leakage the synthetic distribution is already closely aligned with the original manifold.

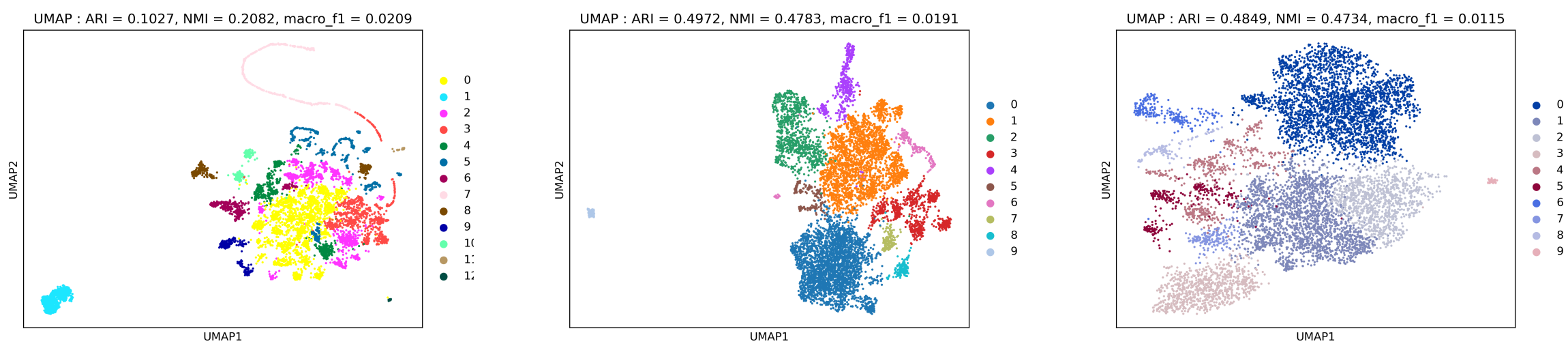

(a) CBMC Real UMAP (b) CBMC Generated UMAP (c) CBMC Combined UMAP

Fig 14: CBMC Dataset: k = 20 (20%)

Figure 14, which shows the UMAP results for cbmc dataset at 20% leakage, exhibits clear distinction in cluster formation compared to lower leakage and real results. The UMAP visualization illustrates compact, clearly separated clusters in the synthetic data, complimenting the significant hike in the metric scores. The combined scores have also shown clear inflation, but still remains slightly lower than the generated results, likely indicating the integration of synthetic data with more noisy real measurements decreases the cluster sharpness slightly. In this case, the model seems to have acquired a richer and fuller description of the cellular landscape, with little mode collapse and clear cell type demarcation.

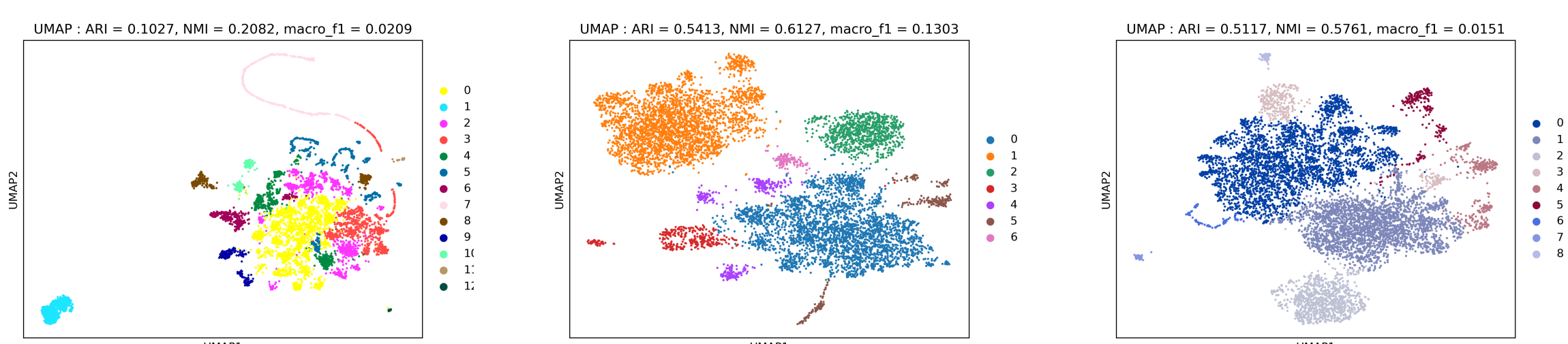

(a) CBMC Real UMAP (b) CBMC Generated UMAP (c) CBMC Combined UMAP

Fig 15: CBMC Dataset: k = 30 (30%)

The UMAP results for consequent metrics for cbmc dataset with 30% leakage is illustrated in Figure 15. Here, the results for level 30 in both the generated and combined cases outperforms the

corresponding cases in level 20. The plots visualize clear-cut and dense synthetic clusters, which suggests that the generator has learned sufficient real data patterns to optimize subtype separation. However, the combined data results still falls short of generated data results, likely being contrained by the noisiness in the real data. While the increased leakage continues to improve synthesized result performance, the decreasing relative gain on the combined dataset suggests that the reward from additional leakage hint that the benefit from further leakage may plateau at this point.

5.3.2 Feature Selection using CV2

For CBMC dataset using CV2-based feature selection, the baseline performance of true expression profiles was comparatively constrained, yielding low performance scores. These modest clustering metrics are indicative of inherent noise and high-dimensional complexity of CBMC single-cell data, in which several highly correlated immune subtypes tend to overlay each other in the latent space. Against this background, the graph-attentional generative method displays a robust denoising capability and a reliable capacity for extracting biologically relevant structure.

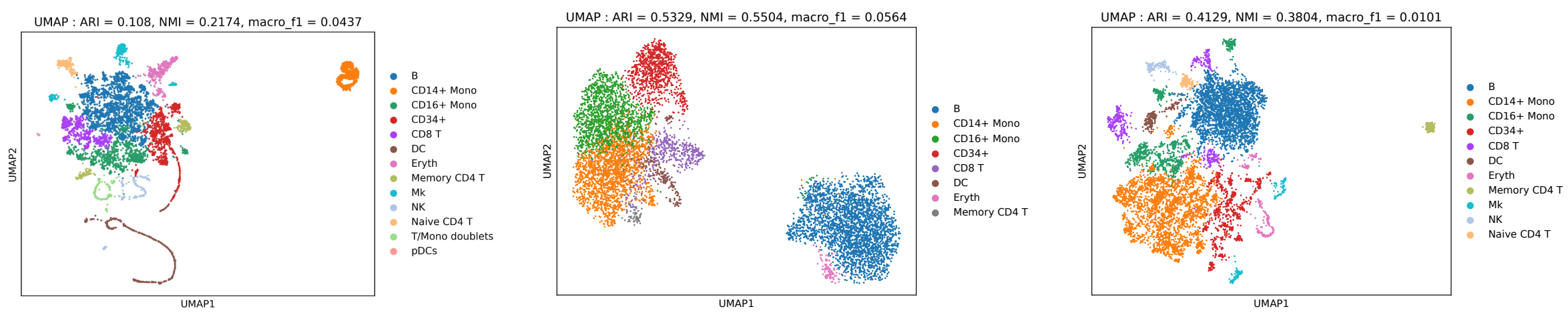

(a) CBMC Real UMAP (b) CBMC Generated UMAP (c) CBMC Combined UMAP

Fig 16: CBMC Dataset: k = 10 (10%)

Figure 16 shows the UMAP results for CBMC dataset using CV2 based feature selection at 10% leakage, the performance being especially heartening. The learned embedding produced ARI and NMI values almost five times more than the baseline, demonstrating the strength of the model in capturing latent cellular relationships in the data that are not apparent from raw data. Both joint and learned embeddings also retain high clustering fidelity, which reveals that generated data closely match actual profiles. This retention of subtype structure despite partial mixing of data proves the robustness of the model in unearthing and aligning inherent transcriptomic manifolds.

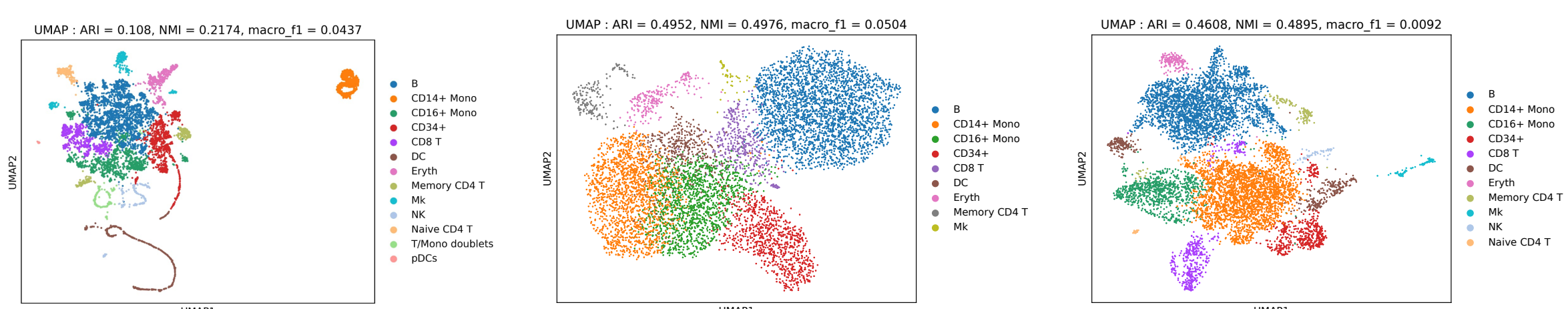

(a) CBMC Real UMAP (b) CBMC Generated UMAP (c) CBMC Combined UMAP

Fig 17: CBMC Dataset: k = 20 (20%)

At 20% leakage, the generative embedding was still robust, though slightly worse than at 10%, according to the UMAP output (Figure 17). Despite this slight decline, the scores are many orders of magnitude over the real-data baseline, proving that GARAGE preserves clustering quality under increased leakage. Surprisingly, the aggregated embedding worked very well, with ARI and NMI scores being an order of magnitude better than the baseline. This enhancement indicates that adding real-data noise assists the model in more accurately modeling sophisticated gene relationships, with overall alignment of clustering being improved despite the loss in the produced data.

The generated data performed best at 30% leakage, with synthetic transcripts reaching peak metric scores, as clearly shown in the UMAP results (Figure 18). This consistent recovery at greater leakage indicates that not only does the model denoise, but it also uses the increased information flow to

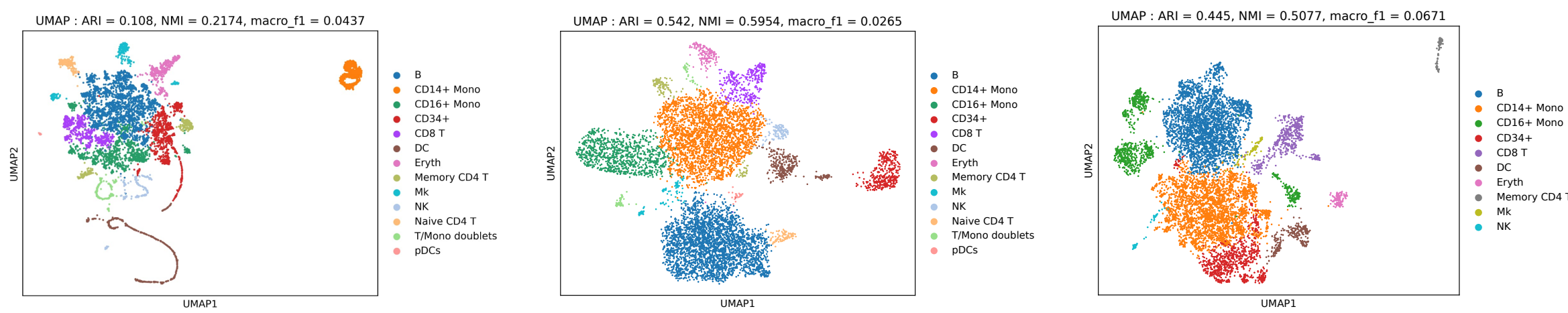

(a) CBMC Real UMAP (b) CBMC Generated UMAP (c) CBMC Combined UMAP

Fig 18: CBMC Dataset: k = 30 (30%)

strengthen subtype boundaries. The combined embedding, although slightly more variable in its values, continued to have values that consistently perform better than the baseline. Notably, the stability of the generative embeddings under greater mixing further confirms the stability of the model's manifold learning process.

As a whole, these findings affirm that the GARAGE model, under CV2 feature selection, achieves significant and stable clustering accuracy improvements in the CBMC dataset. The ARI and NMI enhancements at all levels of leakage denote robust denoising performance and improved subtype discrimination. Furthermore, the macro-F1 increase under moderate leakage indicates an obvious benefit in balancing subtype prediction in diverse immune populations. Overall the model not only enhances clustering accuracy but also maintains stability at various levels of real-data leakage, proving to be effective for robust single-cell data integration. blah blah blah blah

Parameters: For generated data, the (n_neighbors, n_pcs, resolution) are (30, 17, 0.66); (30, 13, 0.62); and (30, 16, 0.52) for 10%, 20%, and 30% leakage, respectively. Now for combined data, the (n_neighbors, n_pcs, resolution) are (30, 17, 0.27); (30, 17, 0.31); and (30, 16, 0.29) for 10%, 20%, and 30% leakage, respectively. For real data, the (n_neighbors, n_pcs, resolution) are (30, 17, 0.38) for each leakage level.

5.4 MURARO Dataset

The Muraro dataset employed in this research includes pancreatic cell populations covered across 10 annotated subtypes, label-encoded as below: 0 = acinar, 1 = alpha, 2 = beta, 3 = delta, 4 = ductal, 5 = endothelial, 6 = epsilon, 7 = gamma, 8 = mesenchymal, and 9 = unclear. The data set has 2,126 cells assayed over 18,902 genes, representing the high-dimensional complexity of pancreatic cell types. For generative modeling, real-data leakage was created at three intensities—10%, 20%, and 30%—to test how differing levels of true biological structure affect the capacity of the model to replicate and maintain subtype-specific patterns in synthetic and blended data.

5.4.1 Feature Selection using Fano index

Following Fano index–based feature selection, the Muraro dataset reveals comparatively low baseline clustering for the real data, so subtype differentiation is only weakly evident. This indicates a more difficult and less clear manifold than for datasets such as Pollen or YAN, offering a tough challenge for the generative model to recover significant structure from sparse leakage. As a reference, we carried out our assessment using the real-data UMAP.

Figure 19 outlines the UMAP results for muraro dataset at 10% leakage, markedly displaying that generated dataset in this configuration yeilds marginally better results than the baseline. The UMAP projection shows more organized and partially aggregated clusters than the real baseline, showing that the generator is starting to retain the essential structure of the cell populations. However, the joint dataset performance declines significantly, indicating that when synthesized samples are combined with the true measurements, noise in actual data overwhelms the embedding structure, weakening the advantage of the smoother distribution of the generator. This means that even at low leakage, although the generator can be a denoiser on its own, it is still not powerful enough to counteract variation in the actual data when clustering jointly. Considering higher level of leakage might be the solution.

The UMAP results for muraro dataset at 20% leakage, illustrated in the Figure 20, shows the improved metric scores obtained, with the synthesized only samples reaching its best performance

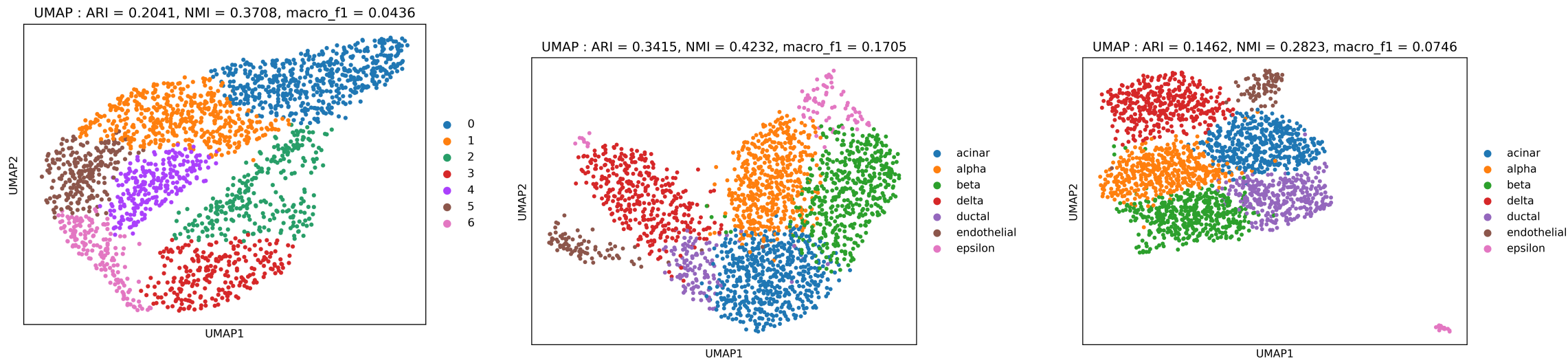

(a) MURARO Real UMAP (b) MURARO Generated UMAP (c) MURARO Combined UMAP

Fig 19: MURARO Dataset: k = 10 (10%)

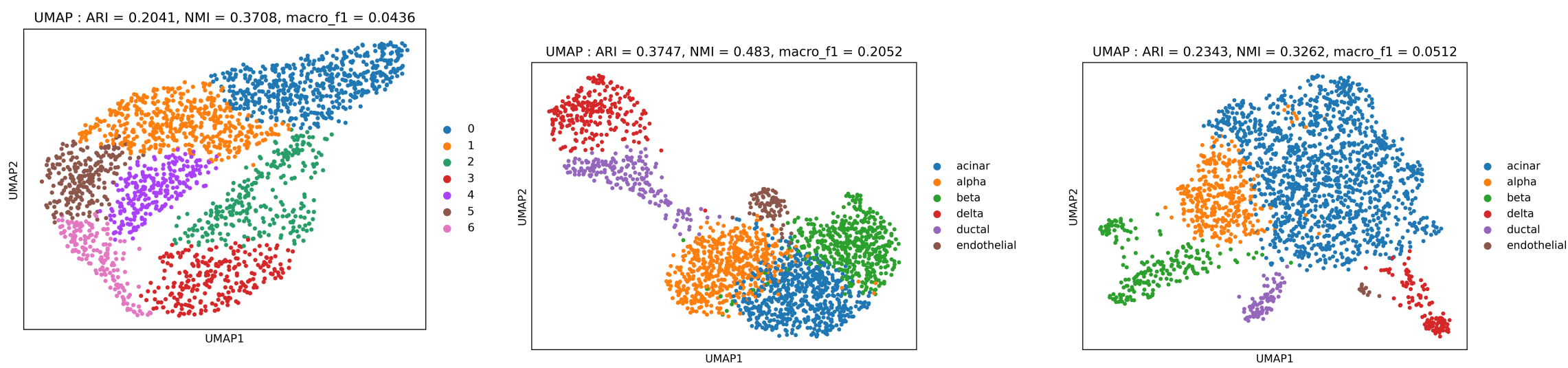

(a) MURARO Real UMAP (b) MURARO Generated UMAP (c) MURARO Combined UMAP

Fig 20: MURARO Dataset: k = 20 (20%)

among all levels of leakage tested. The UMAP plots also have tighter and more uniform clusterings, with certain cell types coming close to full separation. This indicates that the model can learn from further training on real data at this point so that it can represent more nuanced transitions between the subtypes. Surprisingly, the composite dataset also gets better compared to the 10% scenario, but not quite up to the synthetic-only values. This recovery demonstrates that with greater knowledge of real data, the generator starts to exert a stabilizing effect on the joint manifold, but still-inherent noise restricts the best possible clustering quality when both sources are combined.

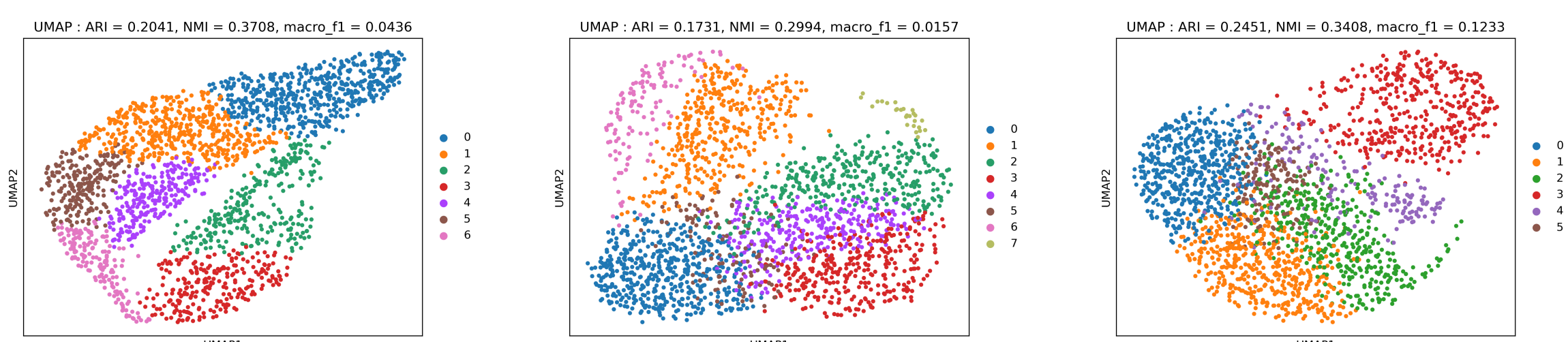

(a) MURARO Real UMAP (b) MURARO Generated UMAP (c) MURARO Combined UMAP

Fig 21: MURARO Dataset: k = 30 (30%)

When there is 30% leakage, the performance metrics of the generated data drop steeply to even lower than the baseline real one in ARI. This degradation is also apparent on the UMAP plots, where clusters generated lose clarity and start diffusing irregularly, indicating overfitting to individual patterns of noise in the actual data rather than generic structural aspects. Contrarily, the combined dataset scores are marginally better than the 20% combined case, but the improvement is minimal. This means that at larger leakage, the generator and actual data find themselves in a position where their errors cancel each other partially in the combined space, but the synthetic data in isolation experiences reduced generalisation ability.

In conclusion, the Fano index-based Muraro experiment demonstrates an asymmetric correlation

between leakage and clustering performance. For results based on synthetic only, 20% leakage seems to be the sweet spot, giving the cleanest cluster formation and best ARI/NMI. But for combined data, gains are small and are still constrained by the noisier nature of the true dataset.

5.4.2 Feature Selection using CV2

The Muraro dataset, following CV2-based feature selection, gives a modest baseline clustering performance for the real data. The low metric scores indicate that while the set of selected features retains some cell-type separability, the clusters are not highly compact, and there is a significant overlap between subtypes in the embedding space. This presents a difficult beginning for the generator and thus serves as an informative case to test the effect of different levels of real data leakage on generative quality.

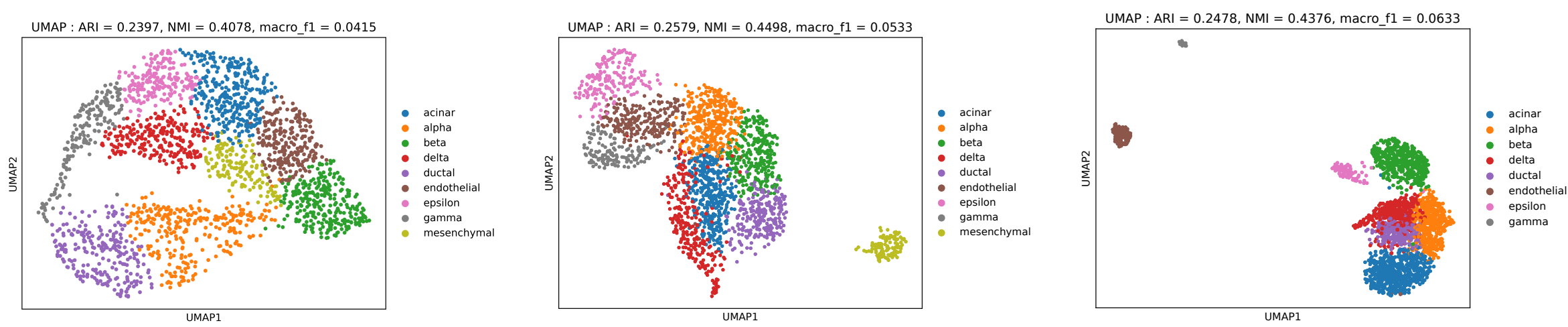

(a) MURARO Real UMAP (b) MURARO Generated UMAP (c) MURARO Combined UMAP

Fig 22: MURARO Dataset: k = 10 (10%)

Figure 22 shows UMAP results for muraro dataset at 10% leakage, displaying a mild increase in the performance for synthesized only samples in comparison to the baseline reference. The UMAP projection demonstrates subtle tightening of some clusters and a bit improved margin separation, showing that even such minimal exposure of real data allows the generator to better perceive more unique subtype boundaries. There remains some dispersion at cluster edges. The union dataset is only a marginal improvement on the baseline, and this suggests that with low leakage, the synthetic data's ability to denoise is not great. Synthetic points are aligned better internally, but introducing real data introduces some variation and noise, so overall gains in cluster alignment are capped.

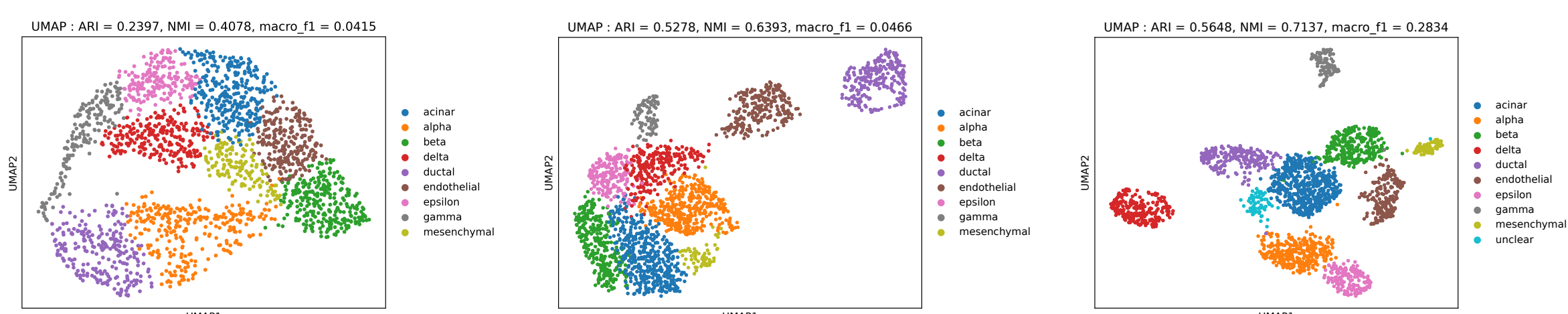

(a) MURARO Real UMAP (b) MURARO Generated UMAP (c) MURARO Combined UMAP

Fig 23: MURARO Dataset: k = 20 (20%)

By 20% leakage, both generated and combined data exhibit balanced performance improvement as evident from the UMAP plots (Figure 23). The ARI for the synthetic data alone is twice that of the baseline, and UMAPs indicate clean clusters with minimal mixing of cell types. Although combined data contain slightly less NMI than the generated-only situation, it is still considerably better than the baseline. This outcome indicates that at this level of leakage, the generator has learned sufficient structural information from actual data in order to represent the global manifold more accurately. The synthetic data seems to serve as a stabilising influence in the joint embedding, smoothing out some of the noise of real measurements to some extent and resulting in more regularised clustering configuration. This is a straightforward demonstration of the denoising effect at work — the generated data smoothes out the cluster edges, resulting in an embedding that is both visually and measurement-wise better than the input.

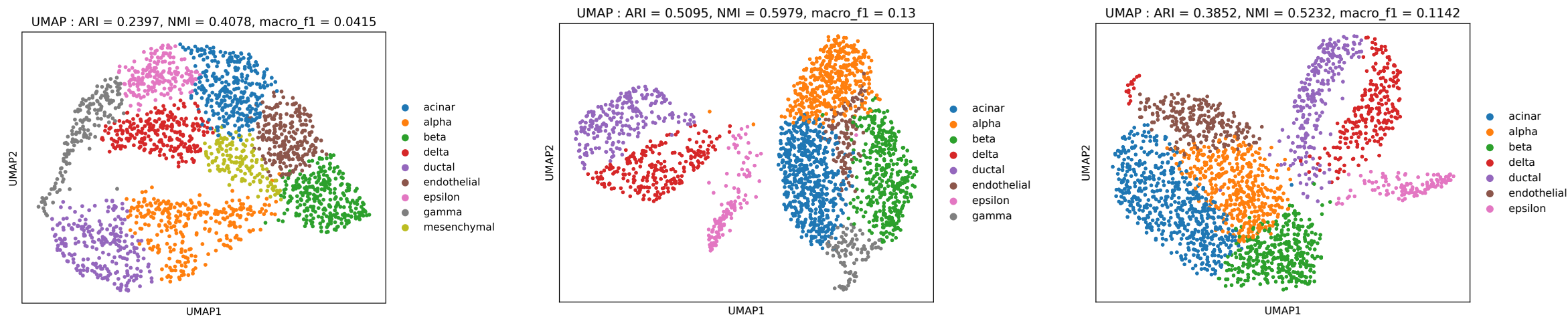

(a) MURARO Real UMAP (b) MURARO Generated UMAP (c) MURARO Combined UMAP

Fig 24: MURARO Dataset: k = 30 (30%)

In the 30% leakage scenario, as illustrated in the UMAP results in Figure 24, the generated data has decently high clustering scores, but both scores falls short from the 20% highs. The UMAP visualization shows that even though the clusters are still recognizable, there is some loosening of compactness and a lot of overlap on the edges. More disturbingly, the joint set suffers a fairly significant drop, indicating that although the generator is still generating fairly well-aligned synthetic data, too much exposure to real data might be causing some overfitting, with the generated samples picking up some of the noise patterns from the original data. Rather than strengthening structure, the actual-synthetic blend at this high leakage condition seems to spoil the cleaner structure alignment created at 20%, watering down the denoising benefit and causing a suboptimal cluster organization.

Together, these findings show that for the Muraro dataset with CV2 feature selection, optimal generative fidelity and overall dataset clustering quality both occur at approximately 20% leakage. At this point, the generator benefits from sufficient real data to capture the manifold's complexity while still leveraging its own capacity to reduce measurement noise. Low leakage does not introduce sufficient structure to enhance embedding fully, whereas high leakage can increase noise instead of sharpening clustering. It is thus essential to balance information gain and noise in order to effectively enhance synthetic–real integration in the embedding space.

Parameters: For generated data, the (n_neighbors, n_pcs, resolution) are (30, 20, 1.02); (30, 20, 0.79); and (30, 20, 0.70) for 10%, 20%, and 30% leakage, respectively. Now for combined data, the (n_neighbors, n_pcs, resolution) are (30, 17, 0.95); (30, 20, 0.47); and (30, 17, 0.61) for 10%, 20%, and 30% leakage, respectively.

6 Supplementary Note 5: Label-agnostic and label-scarce validation

To address the possibility of circularity in label-based evaluation, we performed additional validation analyses that do not rely solely on the cell-type labels used during GAT training. In the main manuscript, ARI, NMI, and macro-F1 were used to evaluate clustering consistency and downstream biological separability. However, because the GAT module uses cell-type labels during training, we further evaluated the generated data using label-agnostic distributional metrics and a label-scarce rare-cell utility experiment.

6.1 Label-agnostic distributional similarity

We computed Maximum Mean Discrepancy (MMD) and Sliced Wasserstein Distance (SWD) between real and generated cells using the full preprocessed gene-expression matrix before feature selection (see table 4). These metrics compare the real and generated transcriptomic distributions without using cell-type labels or clustering assignments. Lower values indicate stronger agreement between the real and generated data distributions.

6.2 Held-out rare-cell utility experiment

To evaluate whether GARAGE-generated cells provide practical utility in a label-scarce setting, we performed a held-out rare-cell experiment. For each dataset, a rare or low-frequency cell type was

Table 4: Label-agnostic distributional similarity between real and generated data computed on the full preprocessed gene-expression matrix before feature selection. Lower MMD and Sliced Wasserstein Distance indicate stronger agreement between real and generated distributions.

Dataset	Method	MMD	Sliced Wasserstein Distance	Feature selection used?
Yan	GAN	0.01356	0.408971	No
Yan	LSH-GAN	0.147763	1.114654	No
Yan	GARAGE	0.083074	0.832318	No
Pollen	GAN	0.569172	3.557356	No
Pollen	LSH-GAN	0.348763	2.543817	No
Pollen	GARAGE	0.198969	2.00228	No
CBMC	GAN	0.125039	0.433174	No
CBMC	LSH-GAN	0.190374	0.550998	No
CBMC	GARAGE	0.011865	0.178887	No
Muraro	GAN	0.215768	0.449762	No
Muraro	LSH-GAN	0.186402	0.417911	No
Muraro	GARAGE	0.104456	0.300054	No

selected, and a subset of its real cells was withheld from training and used only for testing (see table 5). Synthetic cells generated by each model were then used to augment the limited labelled training set. A downstream classifier was trained on the augmented data and evaluated on the held-out real cells. This experiment tests whether the synthetic cells improve recognition of rare cell types in unseen real data rather than merely reproducing label agreement within the training set.

Table 5: Held-out rare-cell utility experiment. A subset of rare-cell examples was withheld from training and used only for testing. Classifiers were trained using real data only or real data augmented with synthetic cells. Higher rare-cell recall, rare-cell F1, and macro-F1 indicate better utility for label-scarce rare-cell recognition.

Dataset	Held-out rare cell type	Training data	Rare-cell recall	Rare-cell F1	Macro-F1
Yan	2cell	Real only	1.0	0.8571	0.9052
Yan	2cell	Real + GAN	1.0	0.6	0.8762
Yan	2cell	Real + LSH-GAN	1.0	0.6	0.8762
Yan	2cell	Real + GARAGE	1.0	0.8571	0.9451
Pollen	GW21	Real only	0.0	0.0	0.5745
Pollen	GW21	Real + GAN	0.5	0.1026	0.444
Pollen	GW21	Real + LSH-GAN	0.5	0.125	0.5286
Pollen	GW21	Real + GARAGE	0.65	0.250	0.8602
CBMC	pDCs	Real only	1.0	0.9796	0.7649
CBMC	pDCs	Real + GAN	0.9583	0.9583	0.7651
CBMC	pDCs	Real + LSH-GAN	0.9167	0.5867	0.4452
CBMC	pDCs	Real + GARAGE	0.9583	0.9583	0.7814
Muraro	endothelial	Real only	1.0	1.0	0.7781
Muraro	endothelial	Real + GAN	1.0	1.0	0.7746
Muraro	endothelial	Real + LSH-GAN	1.0	1.0	0.7751
Muraro	endothelial	Real + GARAGE	1.0	1.0	0.7797

Together, these supplementary analyses provide label-independent and label-scarce evidence for evaluating the fidelity and practical utility of GARAGE-generated cells beyond ARI, NMI, and macro-F1.

7 Supplementary Note 6: Benchmarking Against Existing Generative Models

In order to determine the performance of GARAGE, we compared it with some of the leading generative models for single-cell transcriptomic data, viz., FGAN, WGAN, GAN, LSH-GAN, VAE, scGAN/cscGAN, scVAE, scDiffusion, and oversampling-based GAN/VAE controls. The baselines in-

clude a variety of generative methods, ranging from adversarial training and latent-space reconstruction to single-cell-specific and diffusion-based generative modeling. All the models were trained using the same datasets with equal preprocessing, post-processing, and default or recommended hyperparameters to allow a fair comparison. Benchmarking across datasets of different complexity and noise enabled us to test generative fidelity, denoising, rare-cell preservation, and clustering alignment.

Table 6: Expanded baseline set used for benchmarking GARAGE.

**Baseline group**	**Method**	**Rationale for inclusion**	**Rare-cell handling**
Generic GAN baseline	Standard GAN	Tests performance of adversarial generation from random latent noise.	No explicit rare-cell handling.
Generic VAE baseline	VAE	Tests latent-variable generation and reconstruction-based modeling.	No explicit rare-cell handling.
Adversarial divergence baseline	f-GAN	Tests whether alternative adversarial divergence improves generation.	No explicit rare-cell handling.
Adversarial stability baseline	WGAN	Tests whether Wasserstein objective improves training stability and sample quality.	No explicit rare-cell handling.
scRNA-seq-specific GAN baseline	LSH-GAN	Tests locality-sensitive hashing-guided GAN generation for limited scRNA-seq samples.	Similarity-guided, but not graph-attention-based.
scRNA-seq-specific GAN baseline	scGAN/cscGAN	Tests single-cell-specific GAN generation and conditional generation of cell types.	Can generate cell-type-conditioned samples when labels are available.
scRNA-seq-specific VAE baseline	scVAE	Tests a VAE framework designed specifically for single-cell gene-expression data.	Models count-based single-cell expression but does not use graph-attention-guided rare-cell seeding.
Diffusion-based single-cell baseline	scDiffusion	Tests a recent diffusion-based model for conditional generation of high-quality scRNA-seq data.	Supports condition-guided single-cell generation, including cell-type-specific generation, but does not use GARAGE-style GAT-selected leakage.
Oversampling control	GAN+ROS	Tests whether a standard GAN trained on randomly oversampled rare-cell classes can match GARAGE.	Uses simple random oversampling of minority classes.
Oversampling control	VAE+ROS	Tests whether a VAE trained on randomly oversampled rare-cell classes can match GARAGE.	Uses simple random oversampling of minority classes.
Proposed method	GARAGE	Tests graph-attention-guided leakage of high-priority real-cell anchors into GAN training.	Uses GAT-derived attention scores to select rare-cell-relevant seed cells.

7.1 FGAN

In comparison to f-GAN (Nowozin at al. [9]) for generating scRNA-seq synthetic data, the primary difference between GARAGE and f-GAN is how they augment GAN training and data synthesis. GARAGE, by virtue of its utilization of Graph Attention Networks, is especially well-suited to learning and leveraging the complex relational patterns characteristic of single-cell data and is specifically tuned to generate biologically plausible synthetic data through the use of attention mechanisms. f-GAN, on the other hand, is optimized to stabilize GAN training by matching real and synthetic sample statistics

Table 7: Additional comparison of GARAGE with scRNA-seq-specific, diffusion-based, and oversampling-based baselines.

**Dataset**	**Method**	**ARI**	**NMI**	**F1**
Yan	scGAN	0.5989	0.6763	0.1097
Yan	scVAE	0.7249	0.7808	0.0588
Yan	scDiffusion	0.7249	0.7808	0.0588
Yan	GAN+ROS	0.8234	0.8362	0.1429
Yan	VAE+ROS	0.6427	0.7243	0.1541
Yan	GARAGE	0.935	0.93	0.307
Pollen	scGAN	0.5594	0.7183	0.0223
Pollen	scVAE	0.7121	0.83	0.0018
Pollen	scDiffusion	0.6447	0.7605	0.0204
Pollen	GAN+ROS	0.7479	0.8712	0.0019
Pollen	VAE+ROS	0.6664	0.7922	0.0087
Pollen	GARAGE	0.882	0.896	0.035
CBMC	scGAN	0.1602	0.2524	0.0104
CBMC	scVAE	0.023	0.1147	0.0135
CBMC	scDiffusion	0.0602	0.1948	0.0096
CBMC	GAN+ROS	0.0267	0.1276	0.0139
CBMC	VAE+ROS	0.0848	0.2979	0.0182
CBMC	GARAGE	0.541	0.613	0.13
Muraro	scGAN	0.1213	0.1725	0.0156
Muraro	scVAE	0.1003	0.2155	0.1079
Muraro	scDiffusion	0.2283	0.3371	0.064
Muraro	GAN+ROS	0.1035	0.2422	0.039
Muraro	VAE+ROS	0.3864	0.5294	0.0911
Muraro	GARAGE	0.375	0.483	0.205

in a feature space, enhancing generator convergence. While f-GAN's feature matching mechanism can be used to augment the overall quality of generated data, it lacks the specific capability to model and leverage the graph-structured relationships at the core of scRNA-seq data. Therefore, GARAGE's attention mechanism allows it to selectively focus on important cell-cell interactions and gene regulatory networks and produce synthetic data more reflective of the biological complexity of single-cell datasets. This targeted focus allows GARAGE to better maintain rare cell populations and gene interaction patterns and hence is more appropriate for downstream applications such as feature selection and cell clustering, which are the norm in single-cell research, over the more general training stabilization offered by f-GAN.

7.1.1 Fano Index Feature Selection

Parameters: For the CBMC dataset, the (n_neighbors, n_pcs, resolution) are (20, 30, 0.18) for generated samples and (20, 20, 0.05) for combined samples. For the Muraro dataset, the values are (30, 20, 1.3) for generated samples and (30, 20, 1.1) for combined samples. For the Yan dataset, the parameters are (20, 20, 1.5) for generated samples and (20, 20, 1.15) for combined samples. For the Pollen dataset, the values are (30, 15, 1.29) for generated samples and (30, 20, 2.36) for combined samples.

7.1.2 CV2 Feature Selection

Parameters: For the CBMC dataset, the (n_neighbors, n_pcs, resolution) are (20, 17, 0.13) for generated samples and (30, 10, 0.07) for combined samples. For the Muraro dataset, the values are (30, 19, 1.01) for generated samples and (30, 5, 0.75) for combined samples. For the Pollen dataset, both generated and combined samples share the same parameters: (20, 20, 1.5). For the Yan dataset, the parameters are (20, 17, 1.28) for generated samples and (20, 20, 0.88) for combined samples.

7.2 WGAN

We compare the performance of GARAGE with WGAN (Arjovsky et al. [10]) on all benchmark datasets to evaluate improvements in sample fidelity.

7.2.1 Fano Index Feature Selection

Parameters: For WGAN, the CBMC dataset used (n_neighbors, n_pcs, resolution) = (20, 20, 0.15) for generated samples and (20, 20, 0.25) for combined samples. In Muraro, the values were (30, 15, 1.35) for generated samples and (30, 5, 0.9) for combined samples. For Yan, both generated and combined samples used (20, 17) with resolutions 1.17 and 1.15, respectively. In Pollen, generated samples had (30, 20, 1.95) while combined samples used (30, 18, 1.73).

7.2.2 CV2 Feature Selection

Parameters: For WGAN, the CBMC dataset used (n_neighbors, n_pcs, resolution) = (40, 7, 0.09) for generated samples and (20, 15, 0.10) for combined samples. In Muraro, the values were (30, 17, 1.20) for generated samples and (30, 27, 0.68) for combined samples. For Pollen, generated samples had (20, 20, 1.63) while combined samples used (30, 7, 0.12). In Yan, generated samples used (20, 20, 1.3) and combined samples used (20, 15, 1.2).

7.3 GAN

We compare the performance of GARAGE with GAN (Goodfellow et al. [7]) on all benchmark datasets to observe differences in generative capability.

7.3.1 Fano Index Feature Selection

Parameters: In the GAN framework, for the CBMC dataset,(n_neighbors, n_pcs, resolution) were (20, 20, 0.26) for generated samples and (20, 20, 0.20) for combined samples. In Muraro, the values were (30, 30, 0.55) for generated samples and (30, 6, 0.35) for combined samples. For Yan, generated

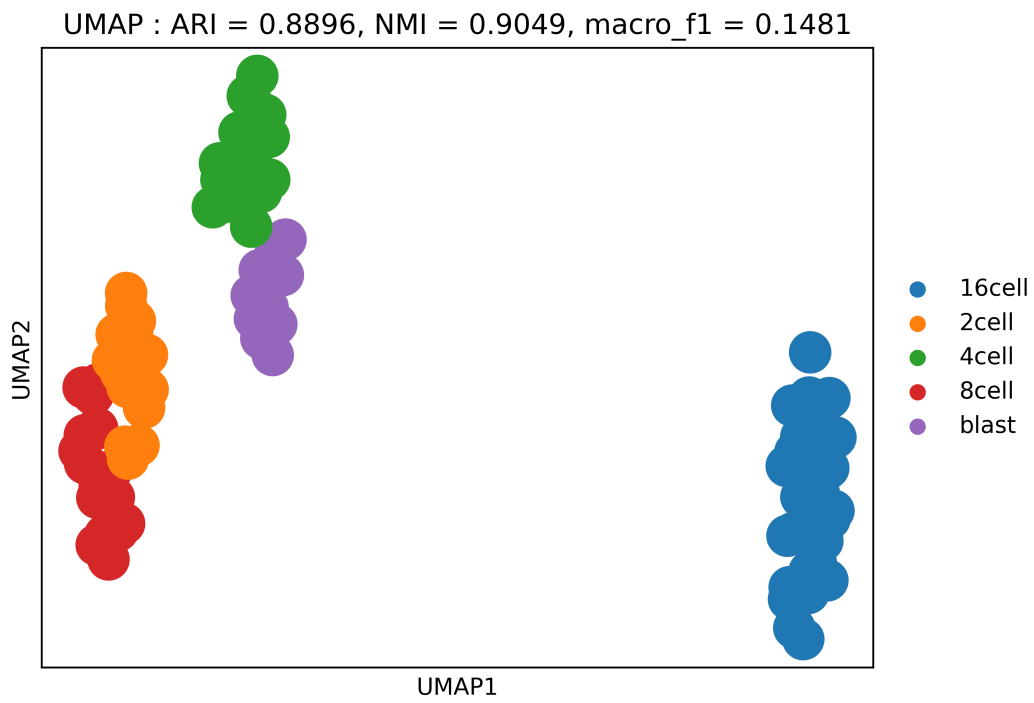

(a) Yan – Generated

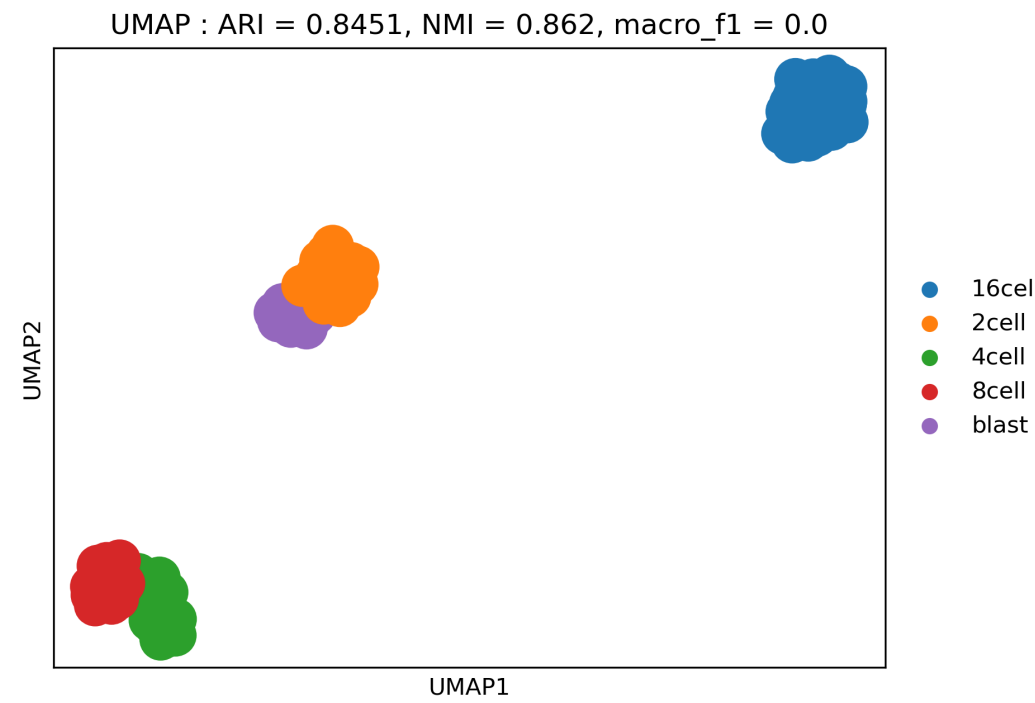

(b) Yan – Combined

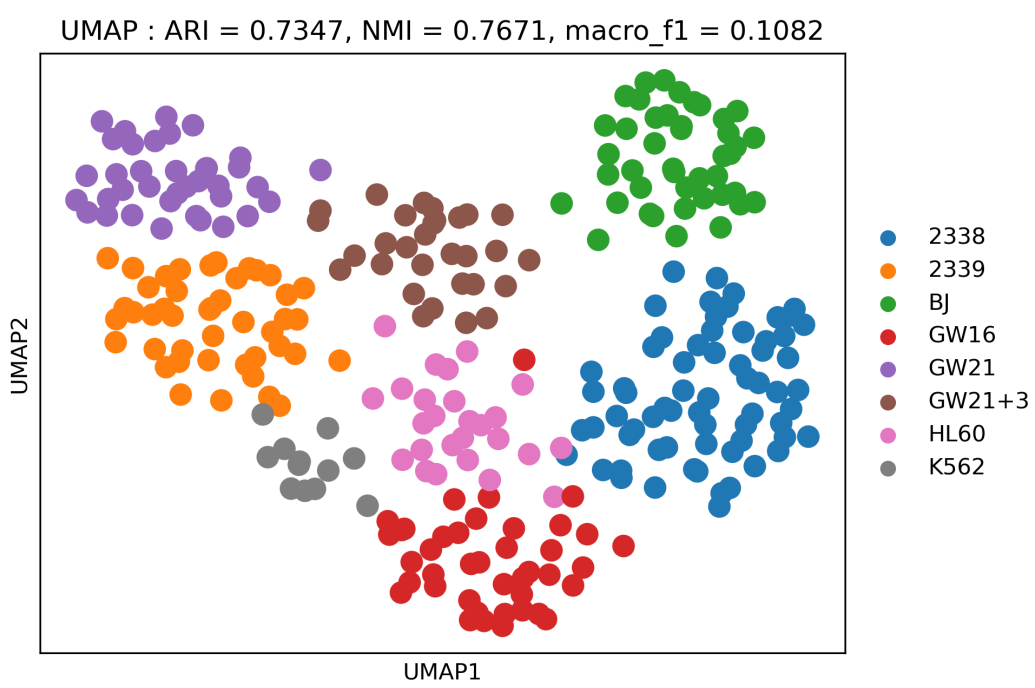

(c) Pollen – Generated

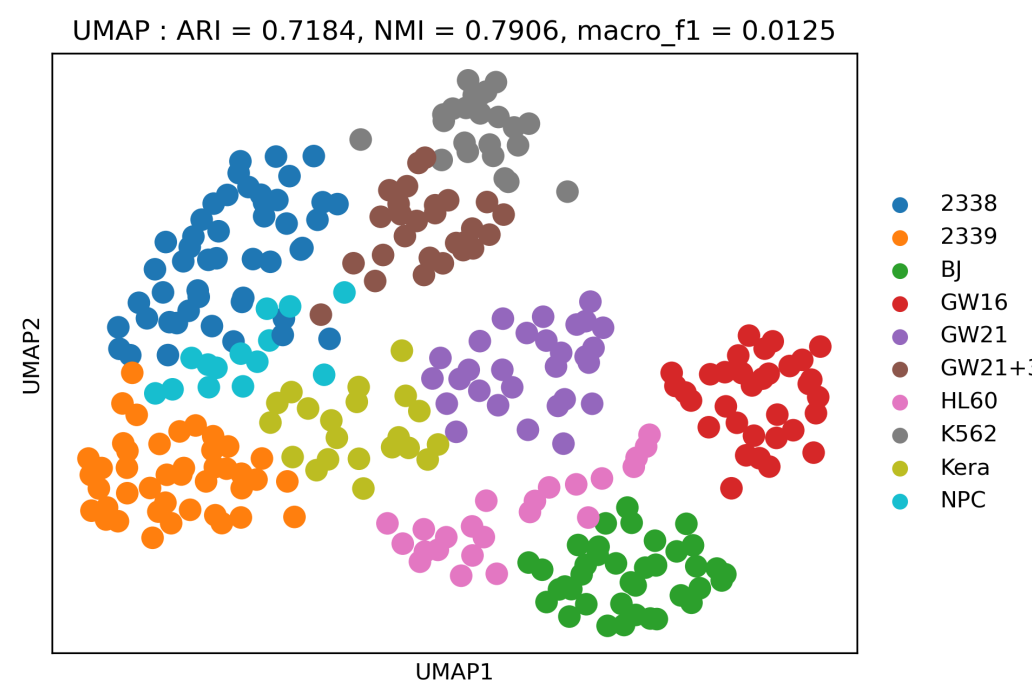

(d) Pollen – Combined

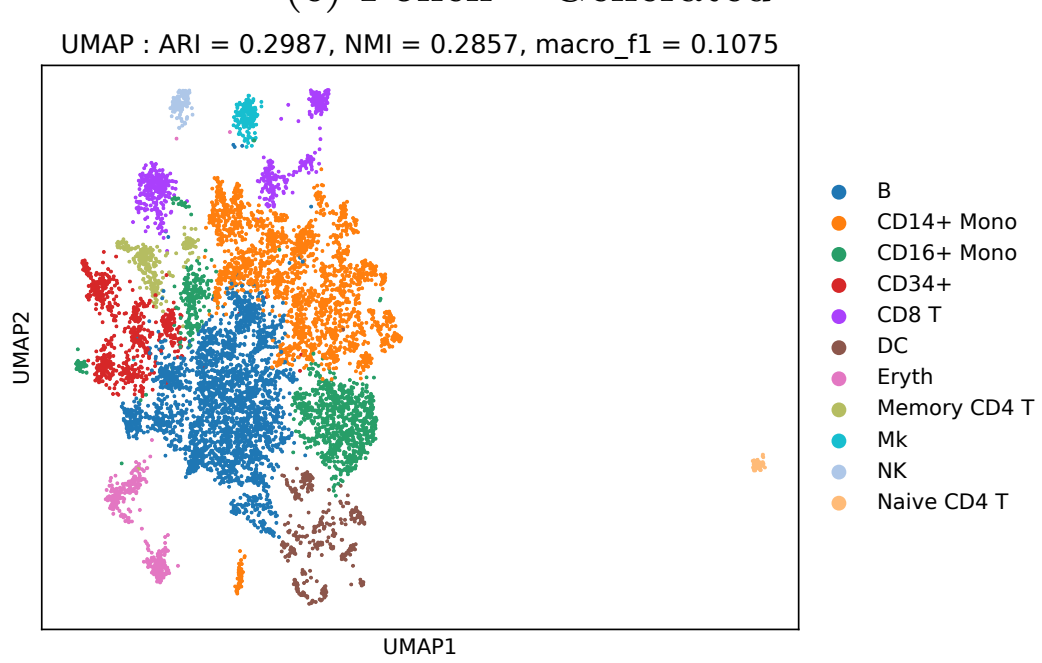

(e) CBMC – Generated

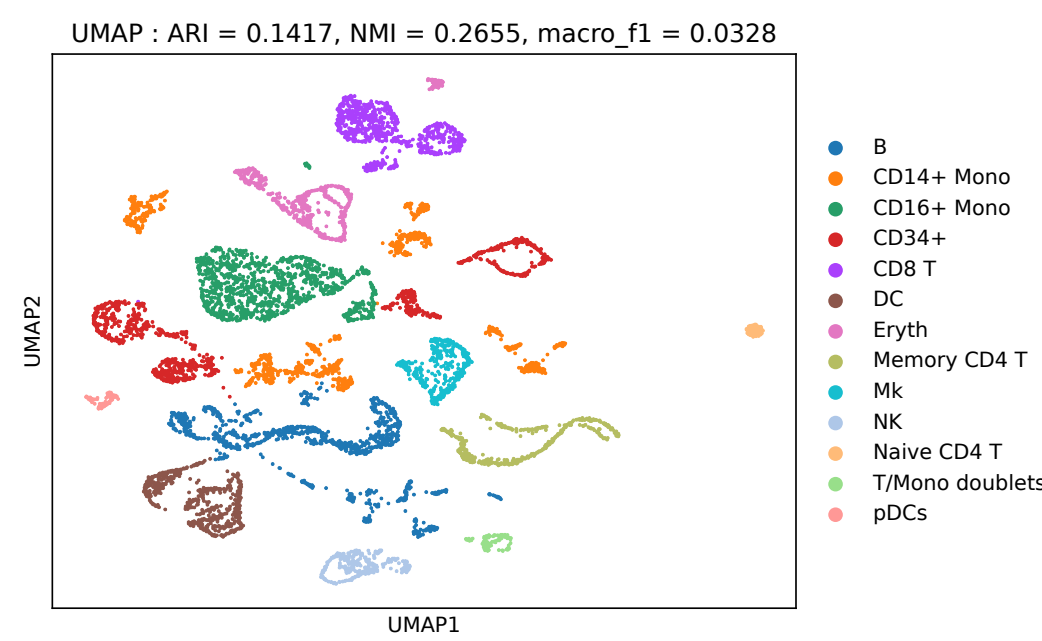

(f) CBMC – Combined

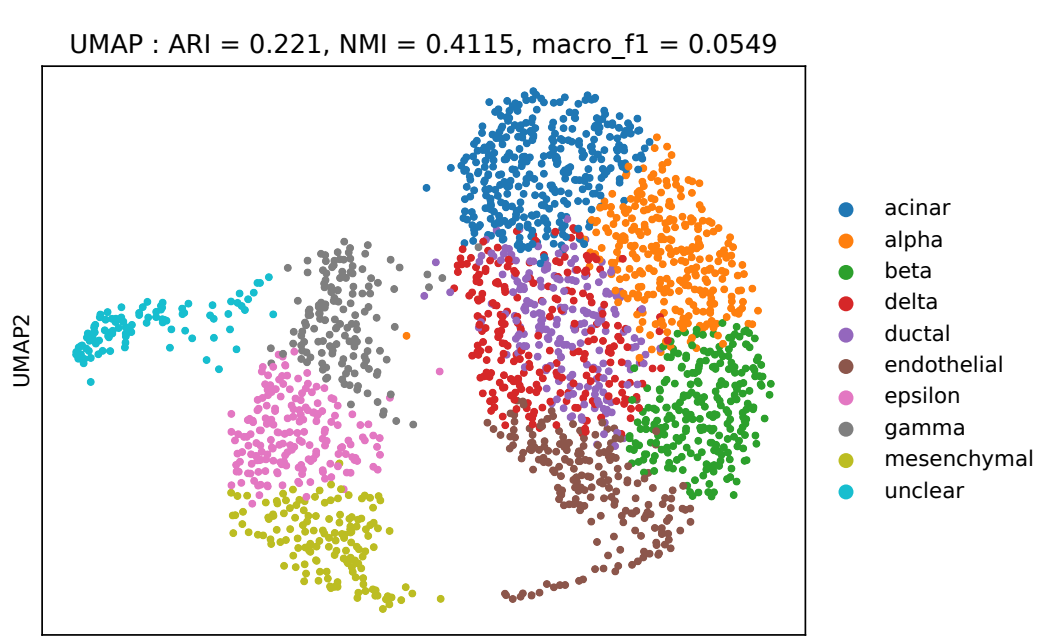

(g) Muraro – Generated

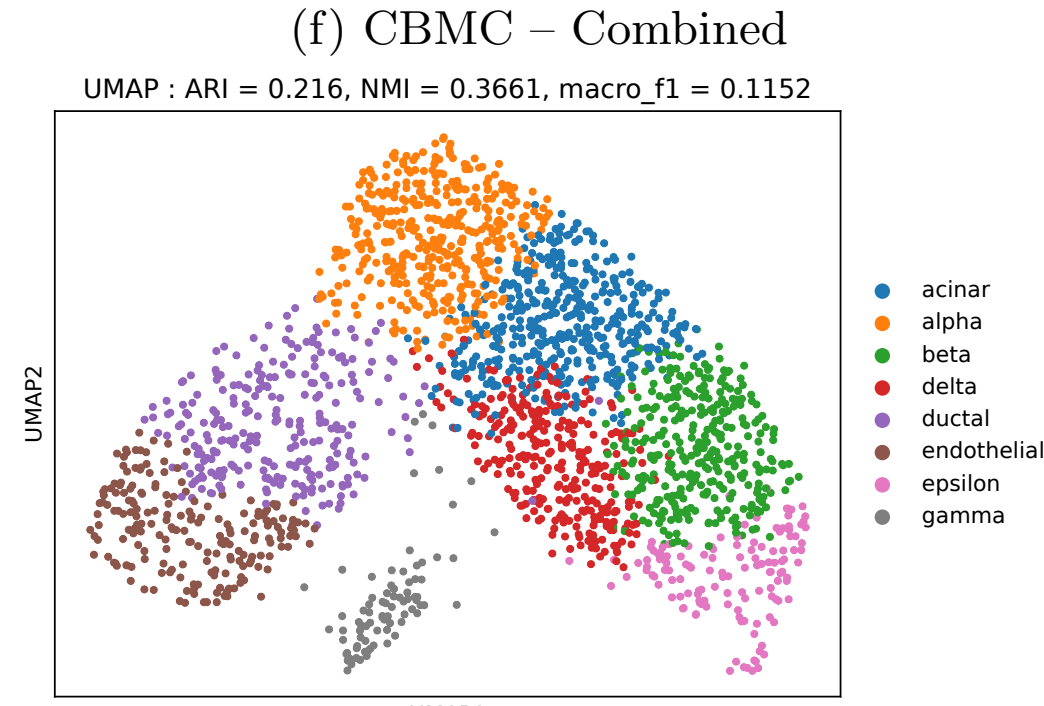

(h) Muraro – Combined

Fig 25: FGAN results for generated(left) and combined(right) dataset across Yan, Pollen, CBMC, and Muraro for feature selection using Fano index.

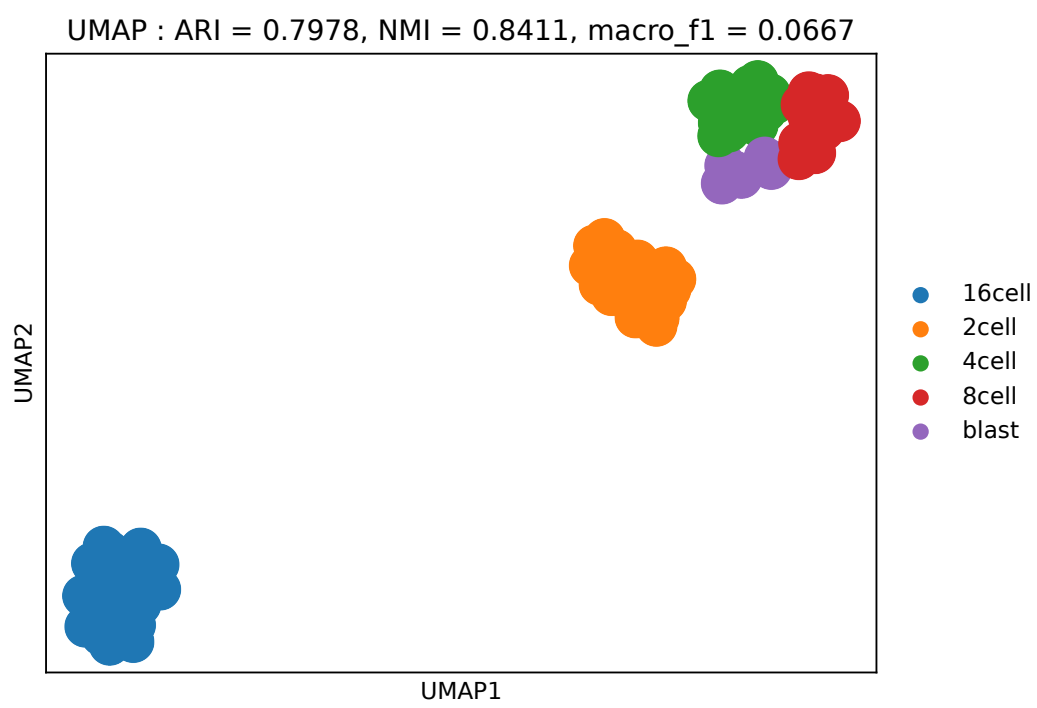

(a) Yan – Generated

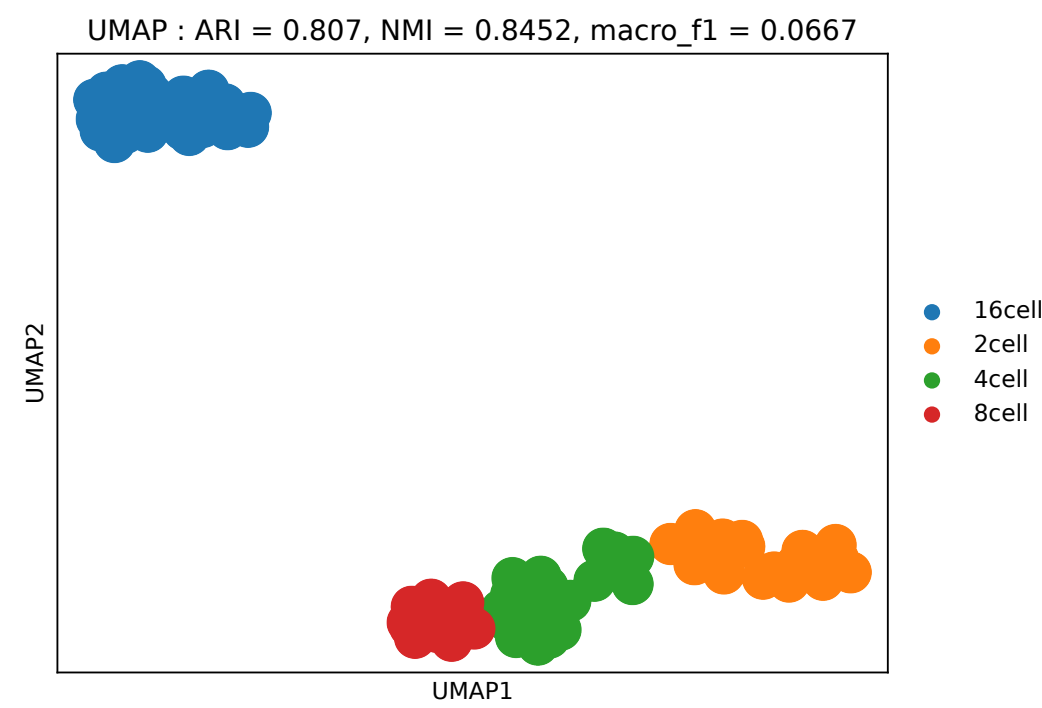

(b) Yan – Combined

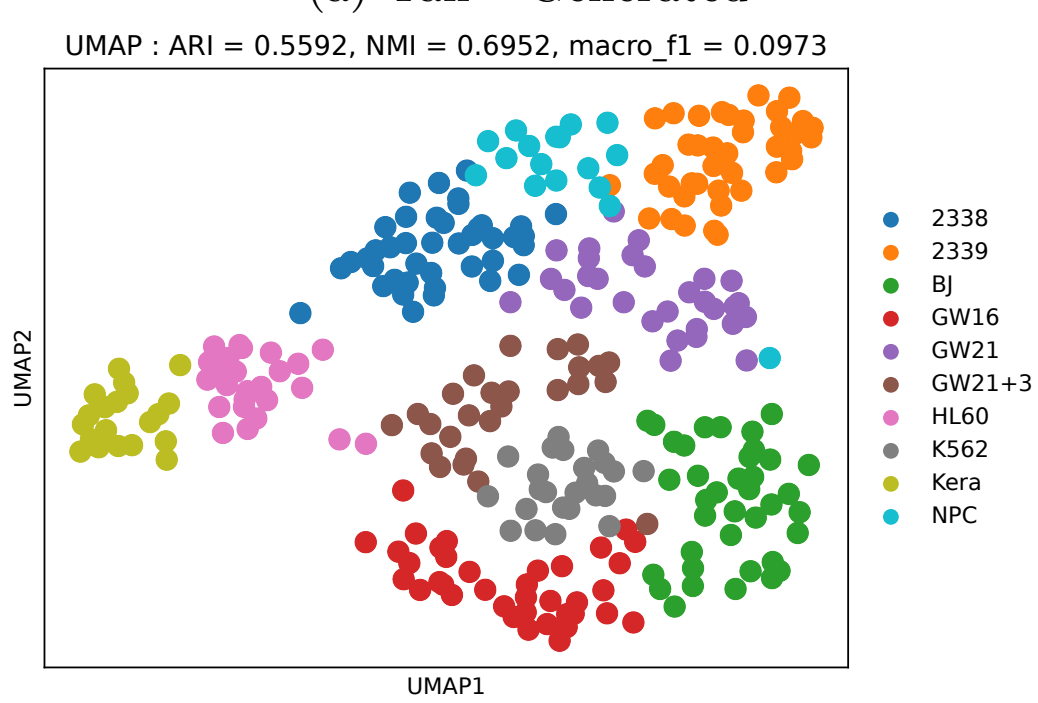

(c) Pollen – Generated

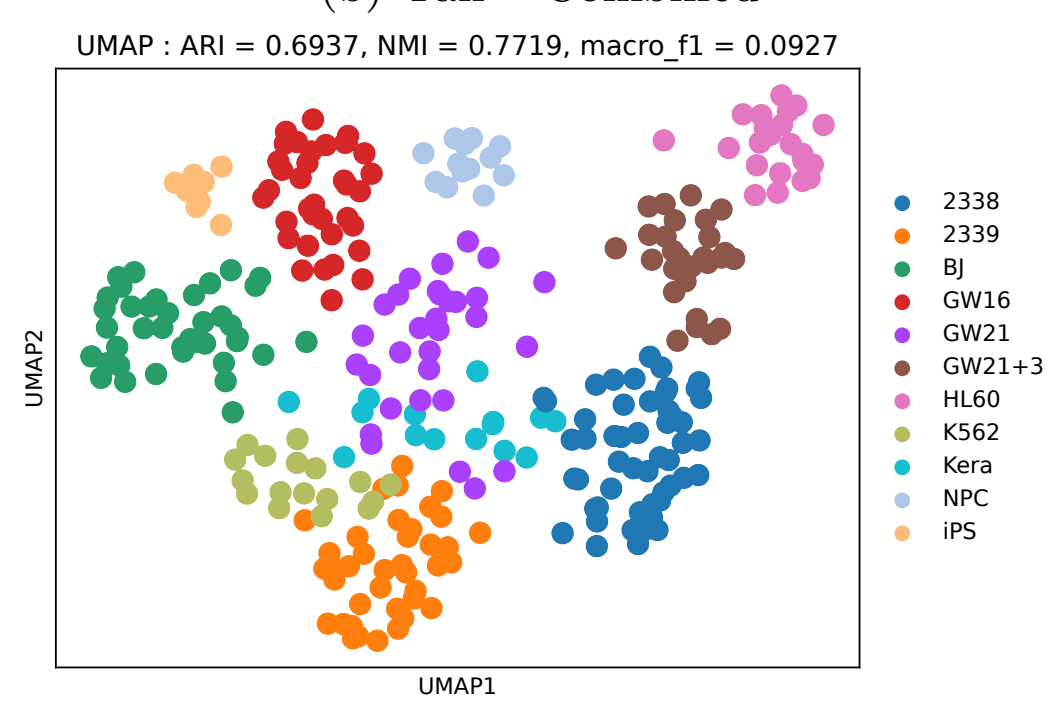

(d) Pollen – Combined

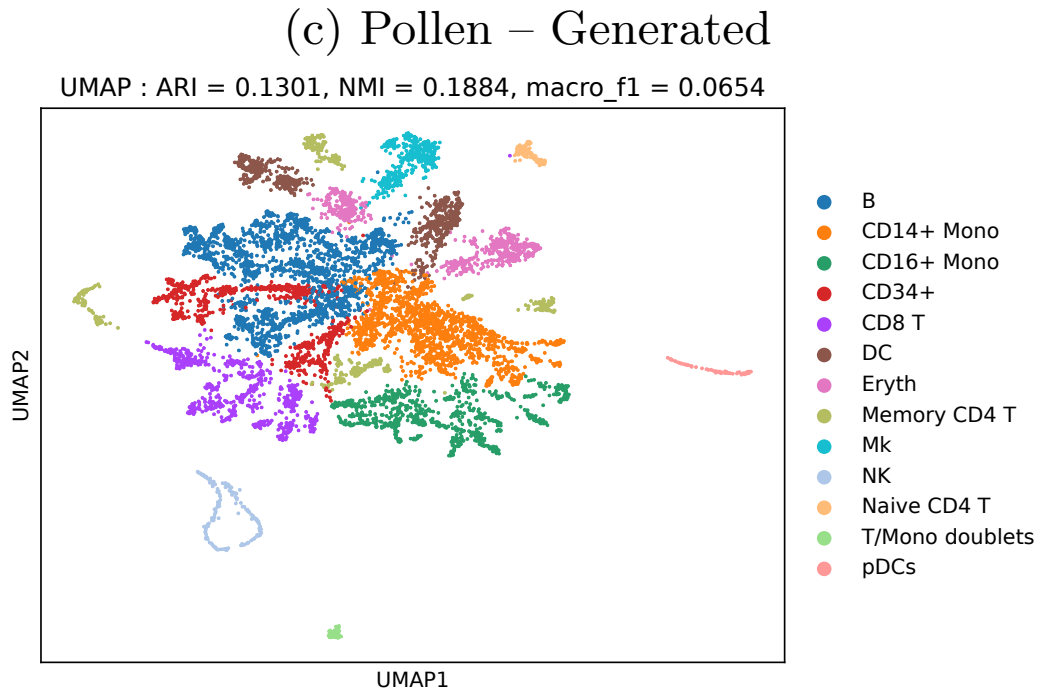

(e) CBMC – Generated

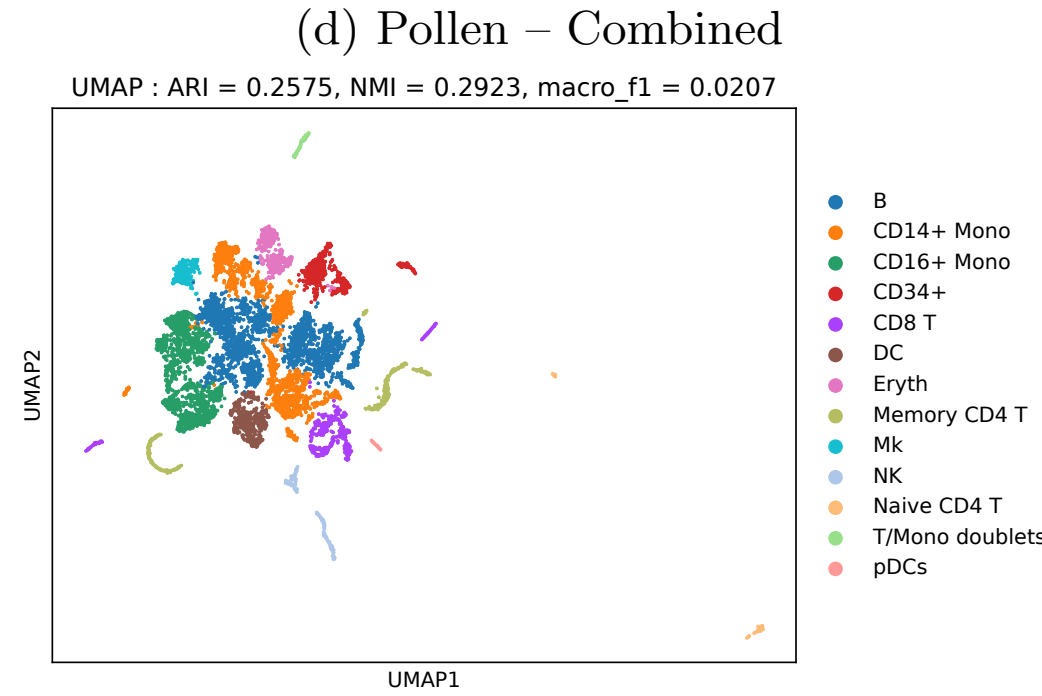

(f) CBMC – Combined

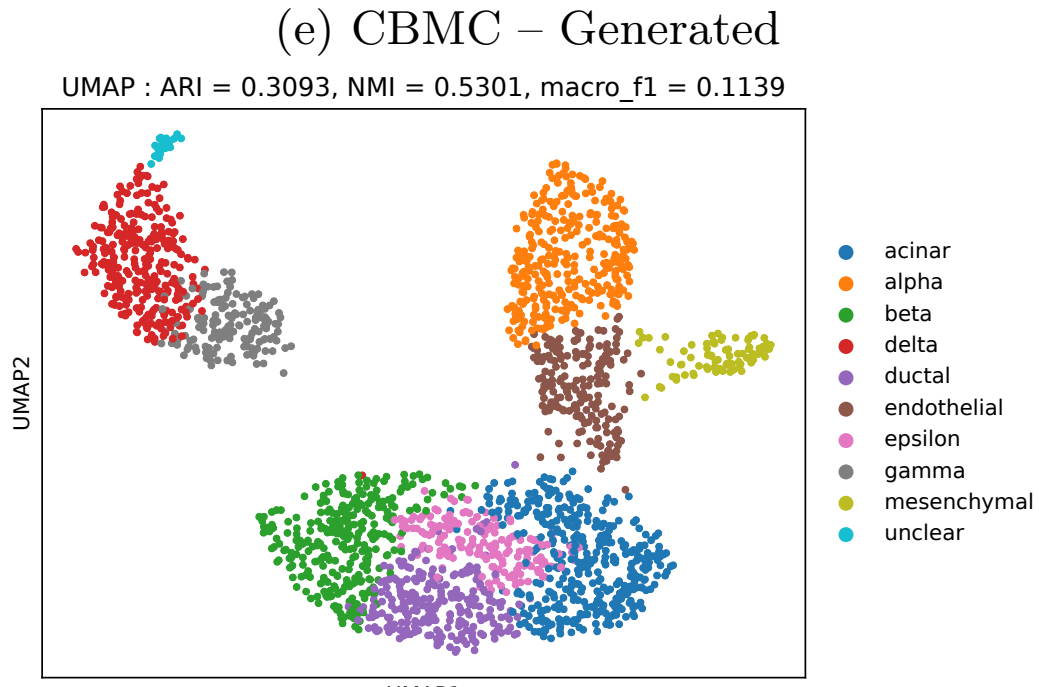

(g) Muraro – Generated

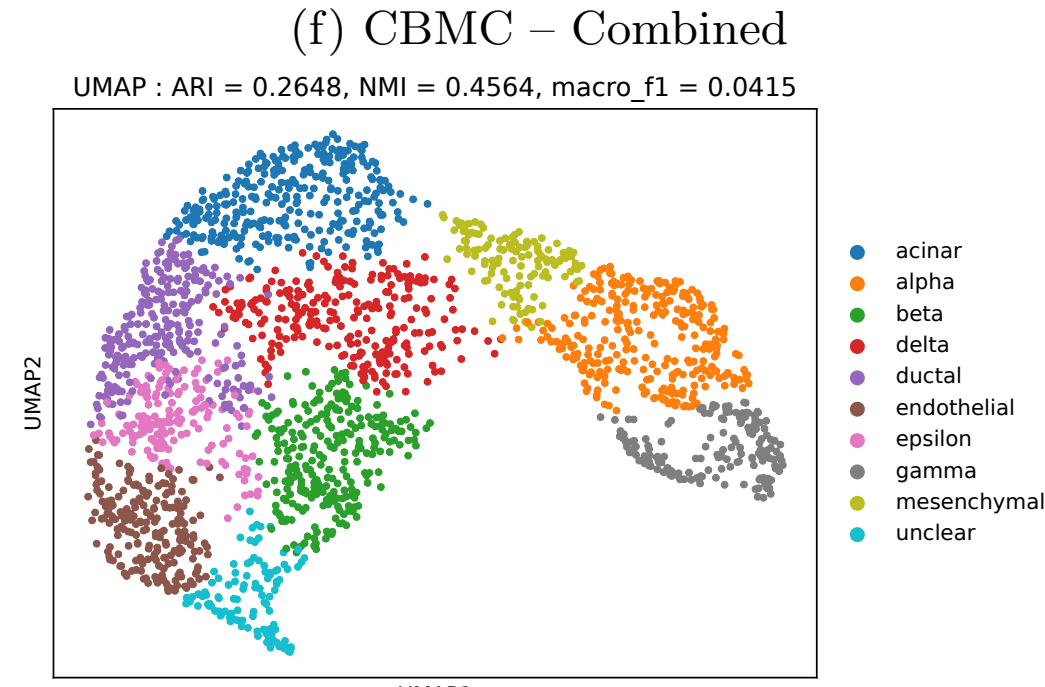

(h) Muraro – Combined

Fig 26: FGAN results for generated(left) and combined(right) dataset across Yan, Pollen, CBMC, and Muraro for feature selection using CV2.

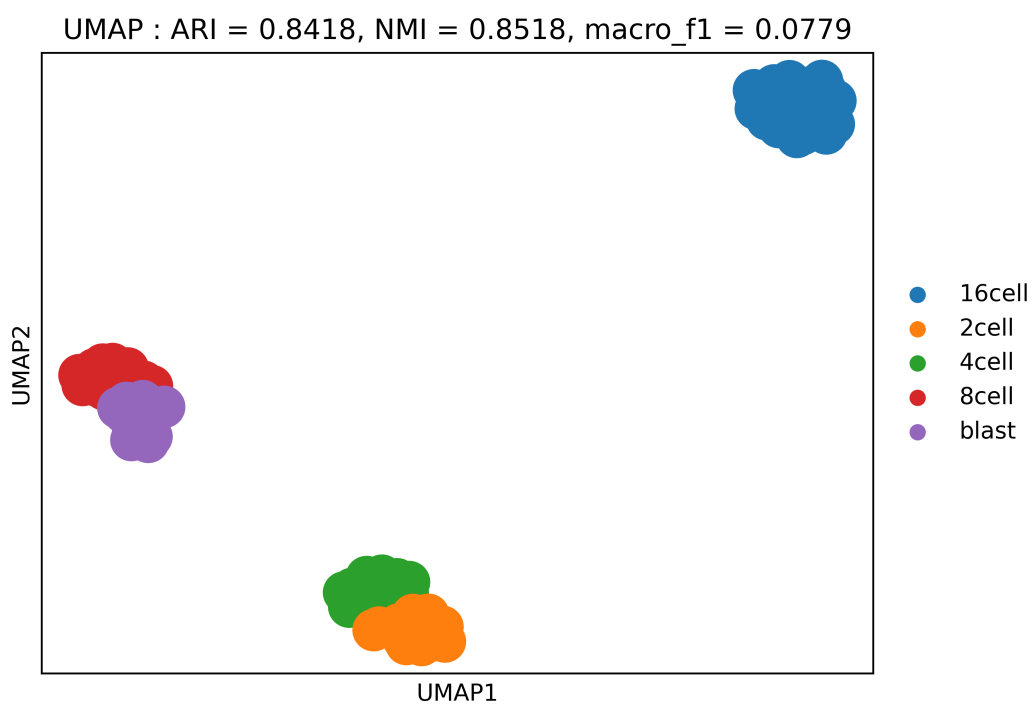

(a) Yan – Generated

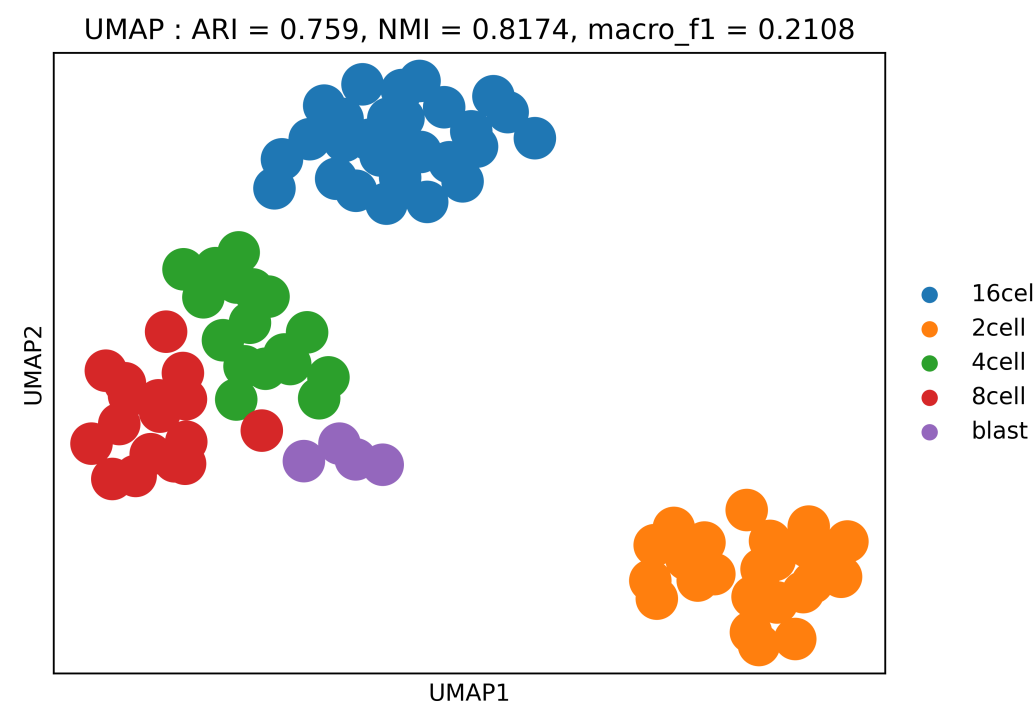

(b) Yan – Combined

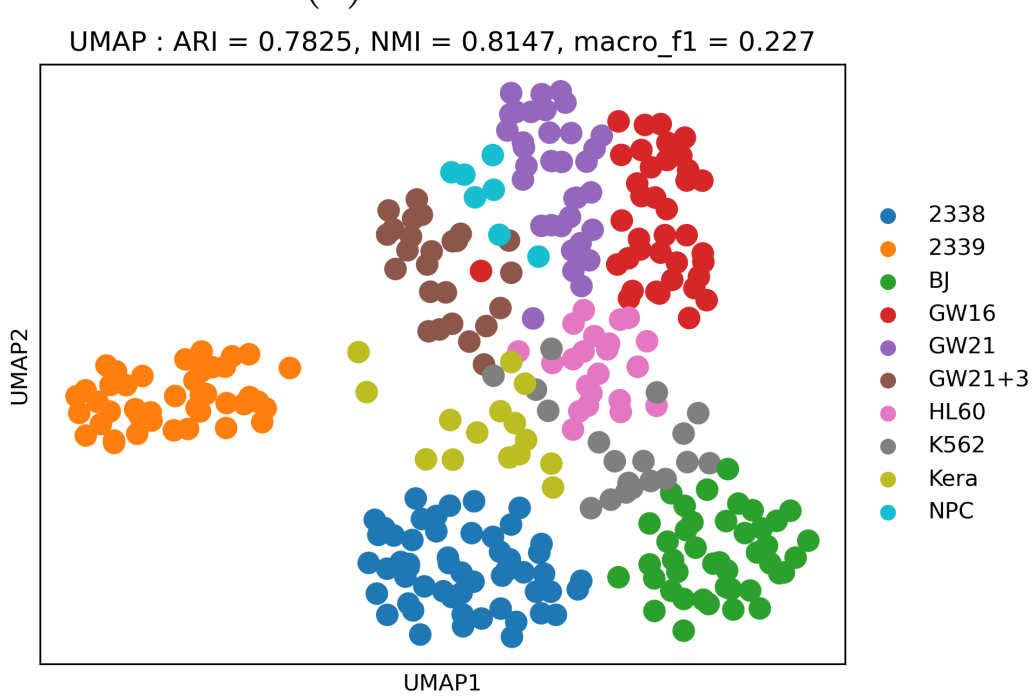

(c) Pollen – Generated

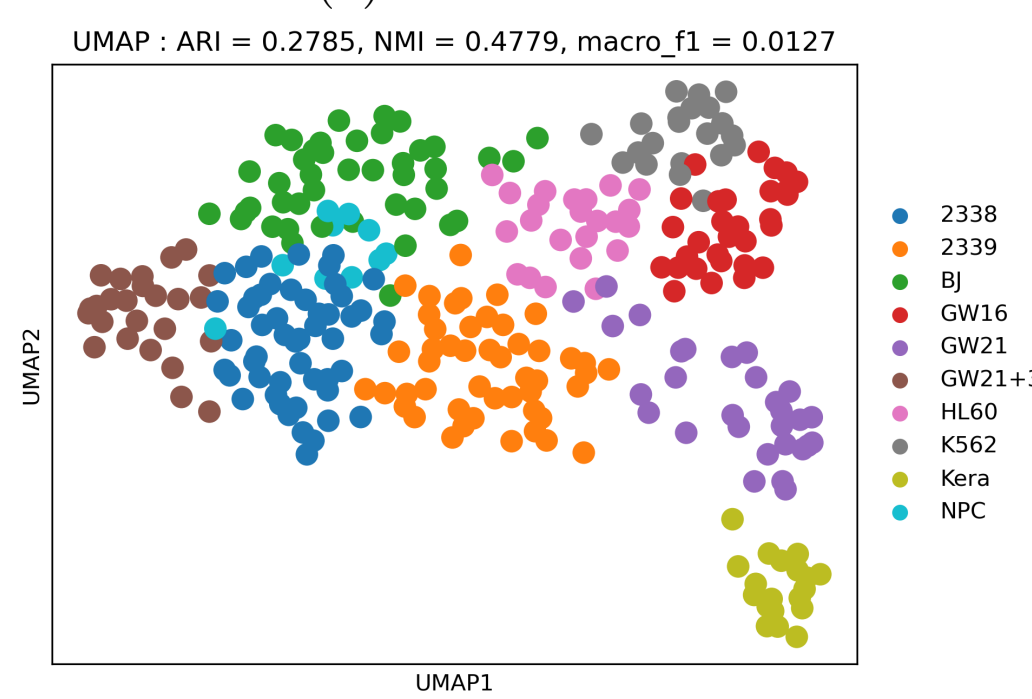

(d) Pollen – Combined

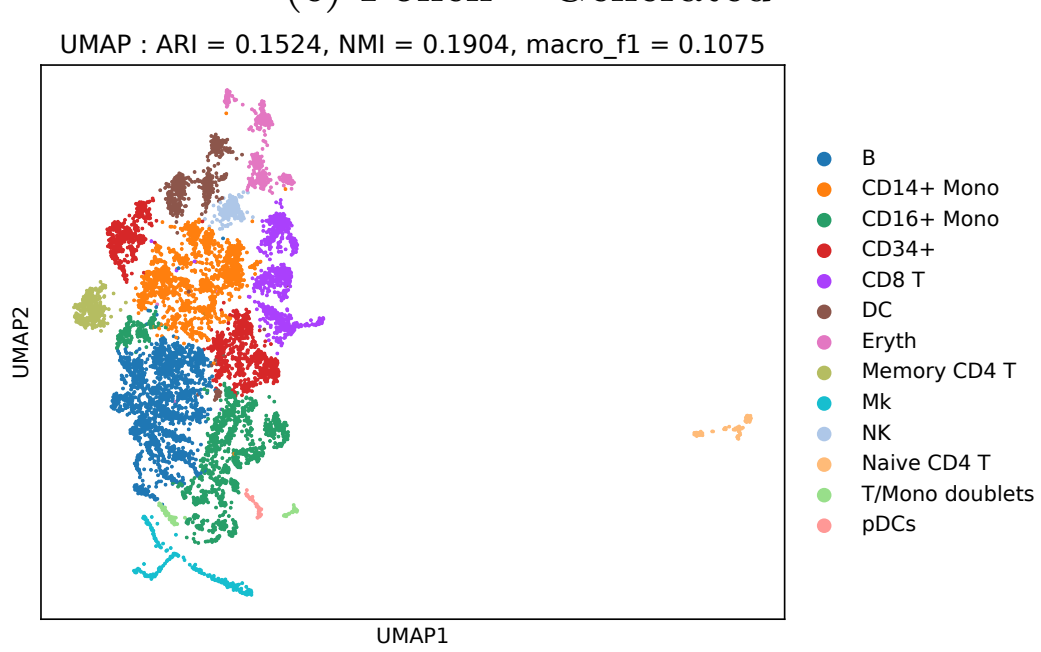

(e) CBMC – Generated

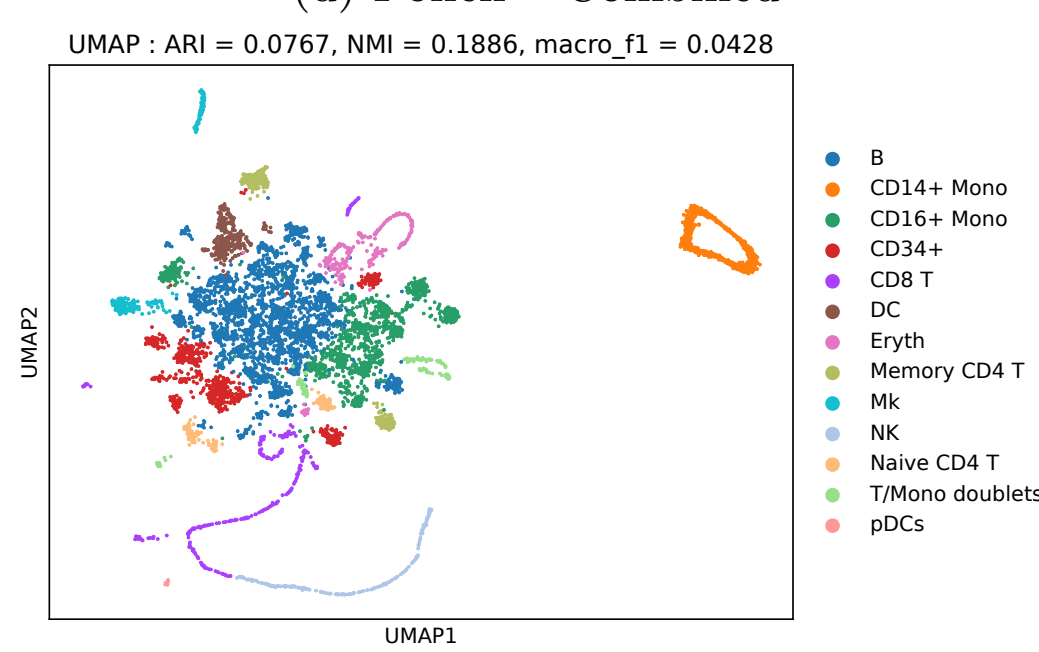

(f) CBMC – Combined

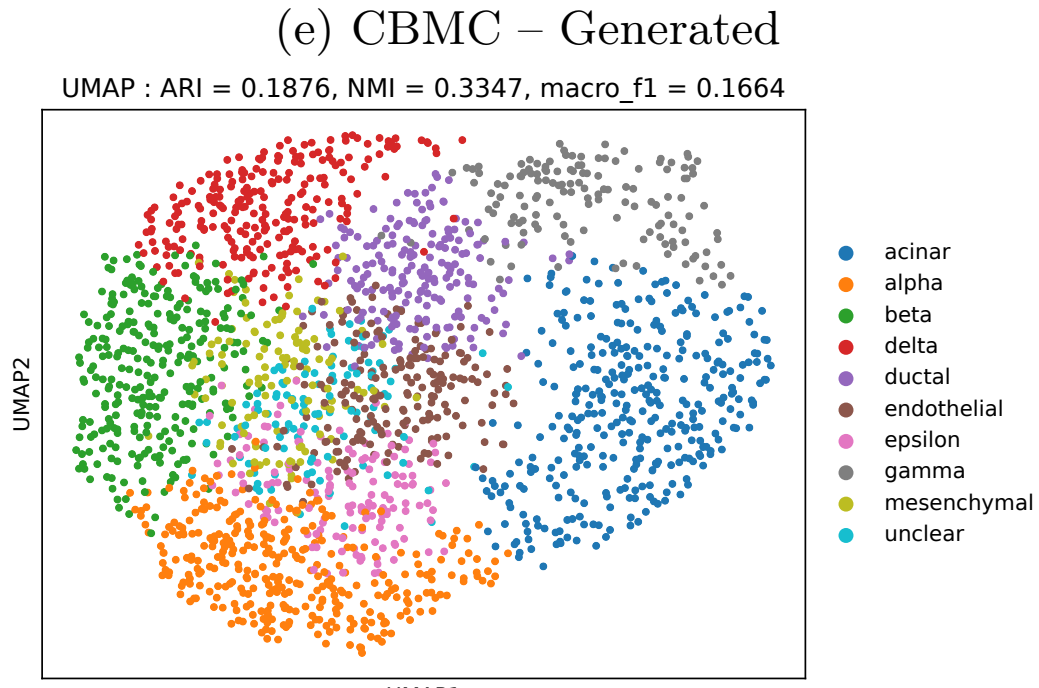

(g) Muraro – Generated

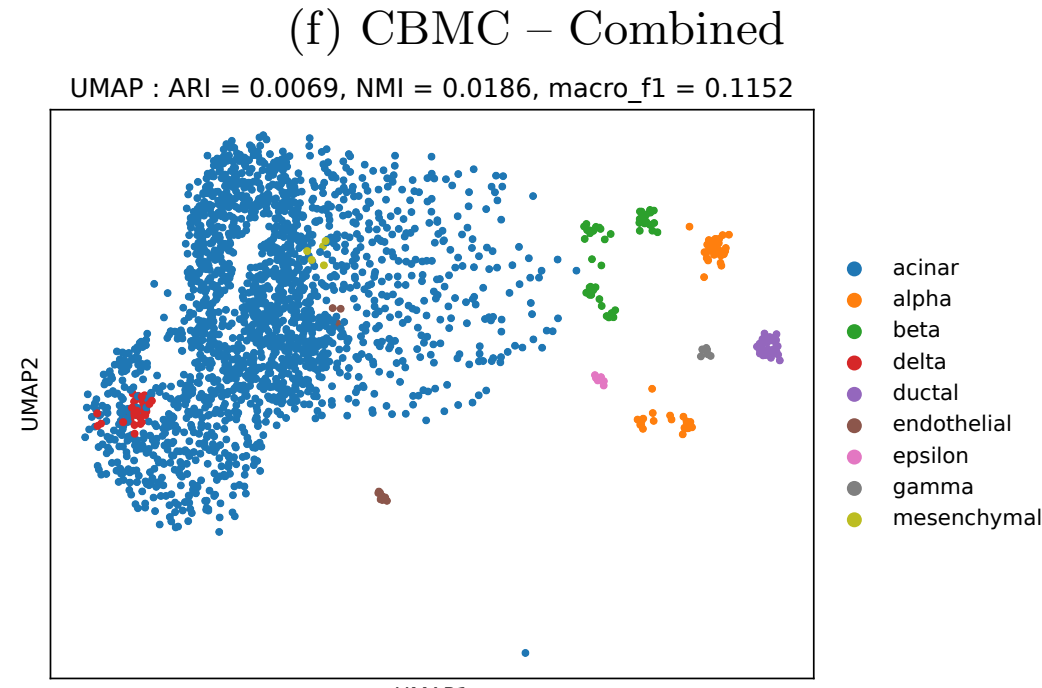

(h) Muraro – Combined

Fig 27: WGAN results for generated(left) and combined(right) dataset across Yan, Pollen, CBMC, and Muraro for feature selection using Fano index.

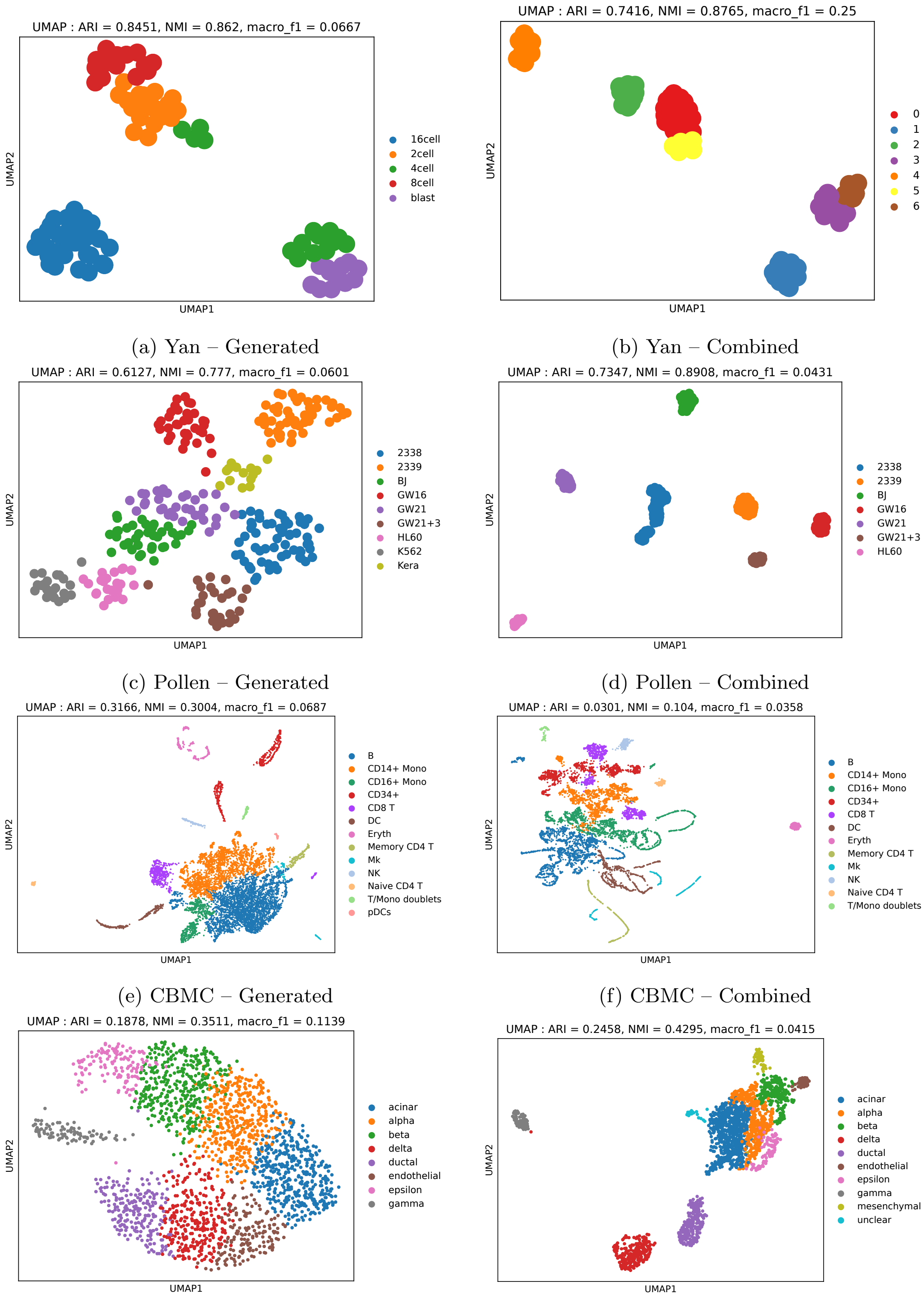

(a) Yan – Generated

(b) Yan – Combined

(c) Pollen – Generated

(d) Pollen – Combined

(e) CBMC – Generated

(f) CBMC – Combined

(g) Muraro – Generated

(h) Muraro – Combined

Fig 28: WGAN results for generated(left) and combined(right) dataset across Yan, Pollen, CBMC, and Muraro for feature selection using CV2.

samples used (20, 20, 1.5) and combined samples used (20, 17, 1.15). In Pollen, generated samples had (20, 20, 1.8) while combined samples used (20, 20, 1.45).

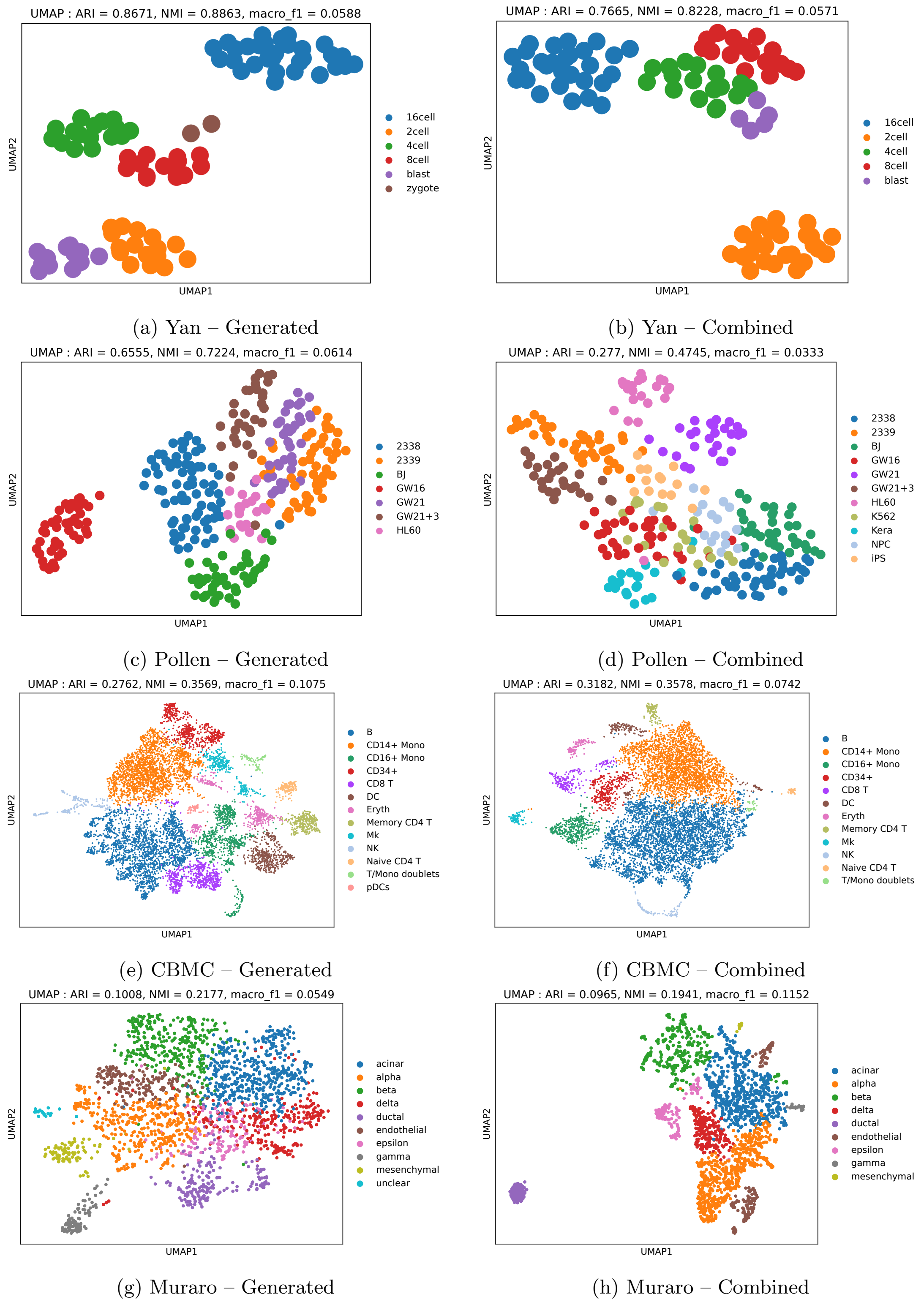

(a) Yan – Generated (b) Yan – Combined

(c) Pollen – Generated (d) Pollen – Combined

(e) CBMC – Generated (f) CBMC – Combined

(g) Muraro – Generated (h) Muraro – Combined

Fig 29: GAN results for generated(left) and combined(right) dataset across Yan, Pollen, CBMC, and Muraro for feature selection using Fano index.

7.3.2 CV2 Feature Selection

Parameters: In the GAN setting, the CBMC dataset used (n_neighbors, n_pcs, resolution) as (20, 28, 0.16) for generated samples and (20, 29, 0.06) for combined samples. In Muraro, generated samples used (20, 20, 0.70) and combined samples used (30, 5, 0.60). For Yan, generated samples had (20, 20, 2.0) while combined samples used (20, 11, 1.3). In Pollen, generated samples used (20, 23, 1.56) and combined samples used (20, 30, 0.34).

7.4 LSH

We compare the performance of GARAGE with LSH (Lall at al. [8]) on all benchmark datasets to examine representation quality.

7.4.1 Fano Index Feature Selection

Parameters: For LSH, the CBMC dataset used (n_neighbors, n_pcs, resolution) as (20, 17, 0.21) for generated samples and (20, 15, 0.20) for combined samples. In Muraro, generated samples used (30, 20, 0.20) and combined samples used (30, 20, 0.25). For Yan, generated samples had (20, 17, 1.37) while combined samples used (20, 17, 1.54). In Pollen, generated samples used (30, 20, 1.2) and combined samples used (33, 30, 1.26).

7.4.2 CV2 Feature Selection

Parameters: For LSH, for the CBMC dataset, (n_neighbors, n_pcs, resolution) were (20, 27, 0.40) for generated samples and (20, 25, 0.30) for combined samples. In Muraro, generated samples used (10, 5, 0.53) and combined samples used (20, 20, 0.43). For Pollen, both generated and combined samples used (20, 20) with resolutions 0.10 and 0.20, respectively. In Yan, generated samples used (20, 20, 1.5) and combined samples used (20, 5, 0.1).

7.5 VAE

We compare the performance of GARAGE with VAE (Kingma et al. [11]) on all benchmark datasets to examine representation quality.

7.5.1 Fano Index Feature Selection

Parameters: For VAE, for the CBMC dataset, (n_neighbors, n_pcs, resolution) were (20, 18, 0.08) for generated samples and (20, 20, 0.20) for combined samples. In Muraro, generated samples used (30, 30, 0.65) and combined samples used (30, 30, 0.97). For Yan, generated samples had (20, 22, 1.5) while combined samples used (20, 17, 1.15). In Pollen, generated samples used (30, 17, 1.06) and combined samples used (30, 17, 1.38).

7.5.2 CV2 Feature Selection

Parameters: For VAE, for the CBMC dataset, (n_neighbors, n_pcs, resolution) were (20, 17, 0.10) for generated samples and (20, 20, 0.10) for combined samples. In Muraro, generated samples used (20, 20, 0.55) and combined samples used (20, 20, 0.48). For Pollen, generated samples had (20, 5, 0.50) while combined samples used (20, 2, 0.20). In Yan, generated samples used (20, 20, 1.5) and combined samples used (20, 27, 1.4).

7.6 Fixed-marker-gene clustering analysis

To reduce the dependence of clustering performance on model-specific feature selection, we performed an additional evaluation using fixed biological marker-gene sets. For each dataset, marker genes were selected independently of the generated data using known cell-type marker information and/or marker annotations reported in the original dataset studies. The same marker-gene set was applied to all generative methods for a given dataset. Therefore, this analysis evaluates whether the generated data preserve biologically meaningful structure under a common, fixed feature space. Table 8 and table 9 show the complete results.

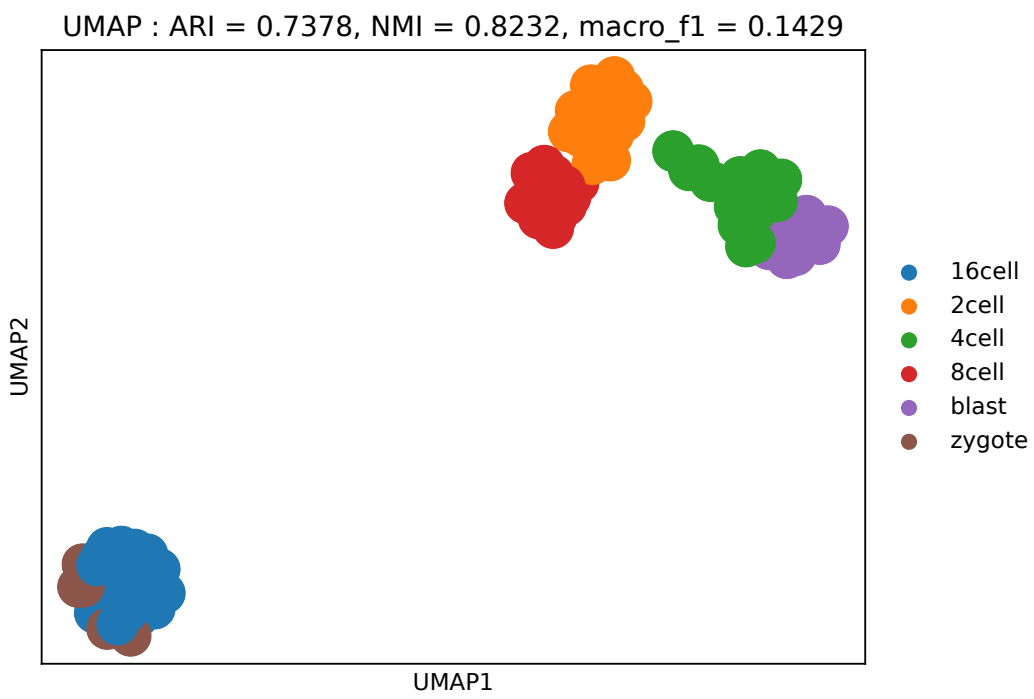

(a) Yan – Generated

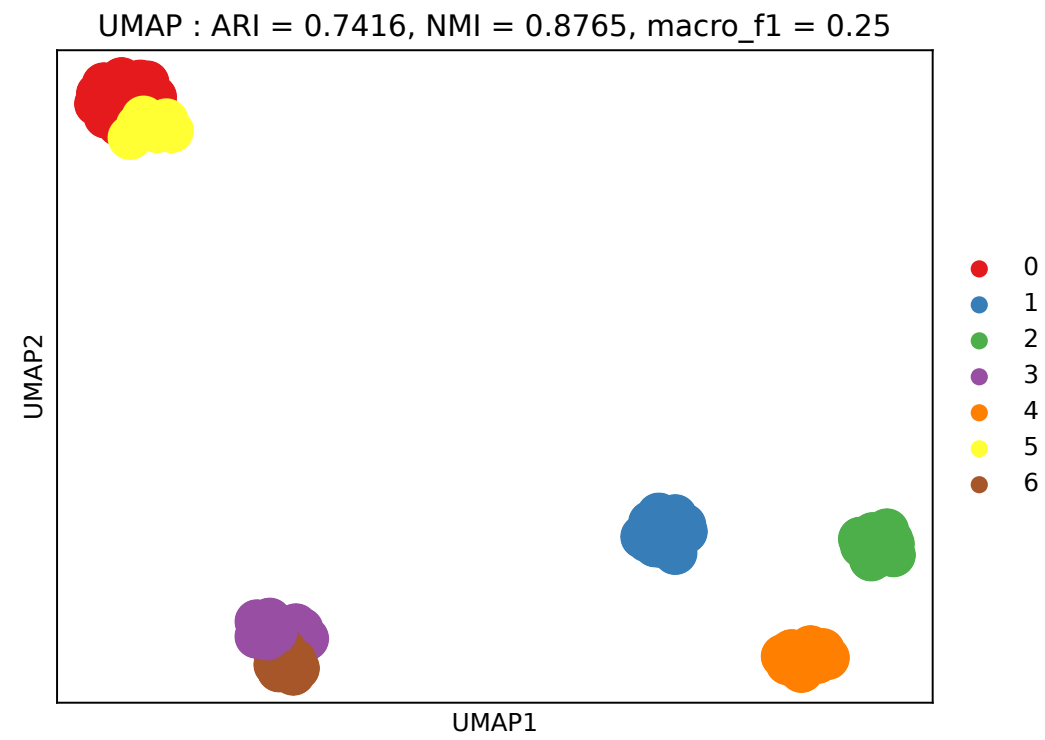

(b) Yan – Combined

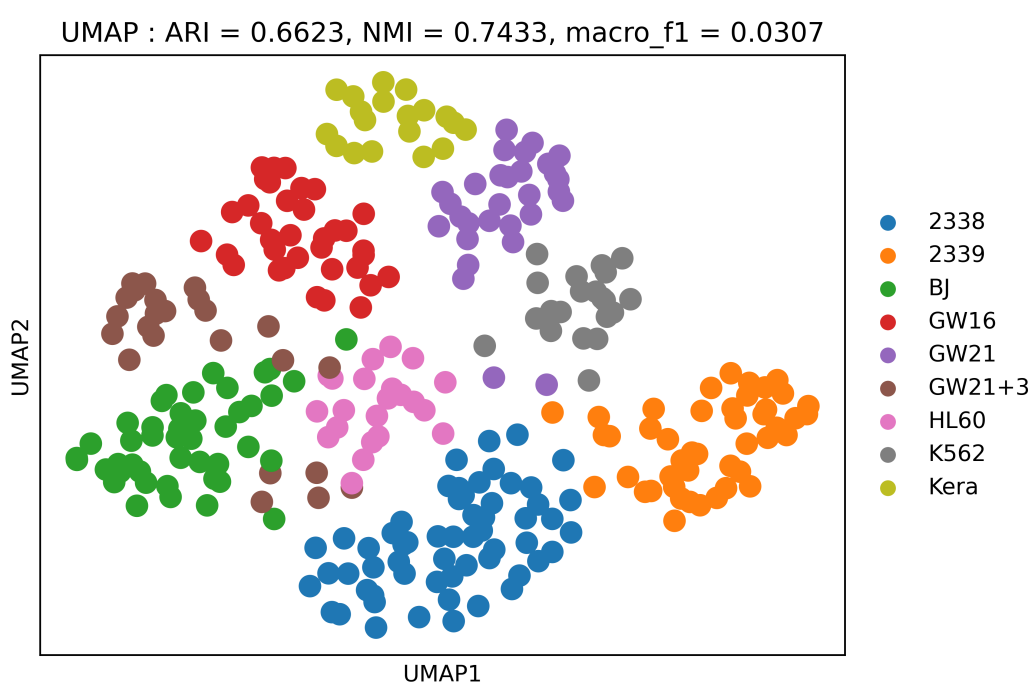

(c) Pollen – Generated

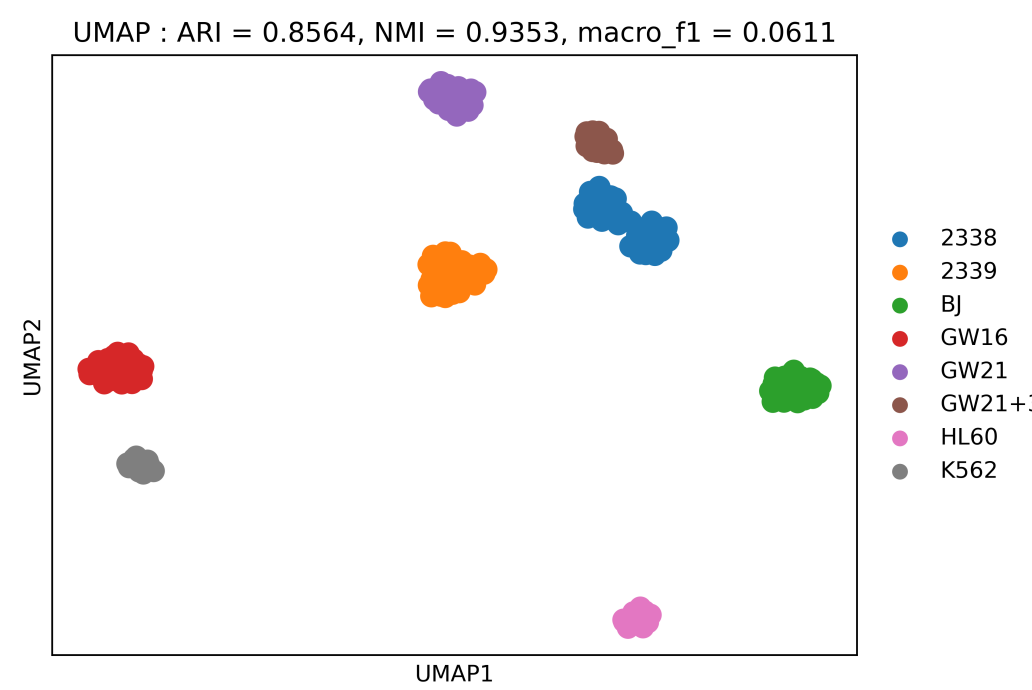

(d) Pollen – Combined

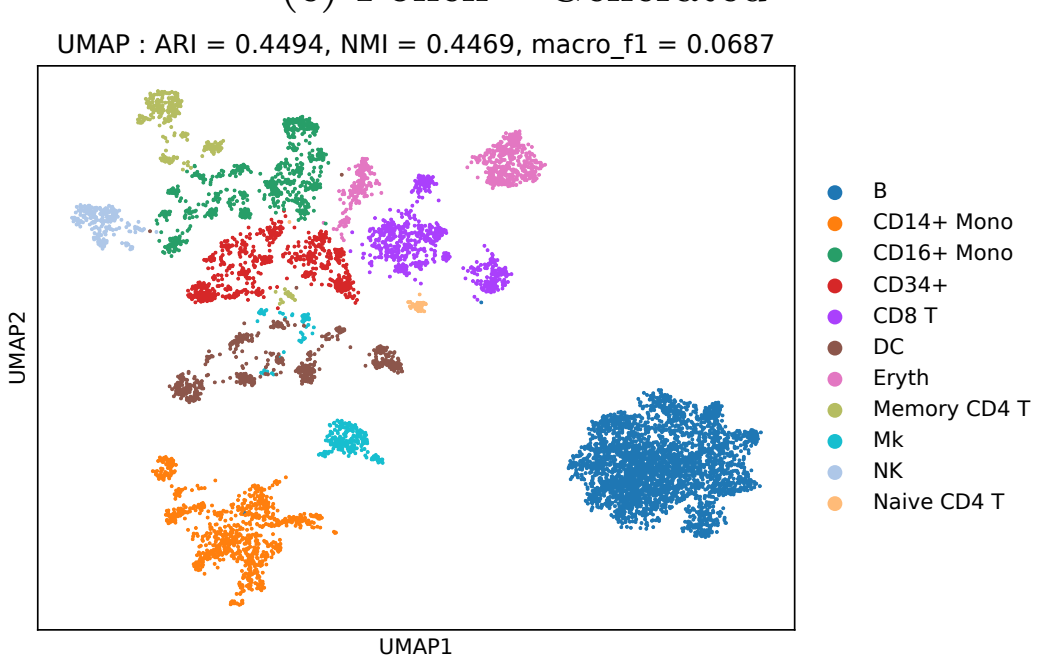

(e) CBMC – Generated

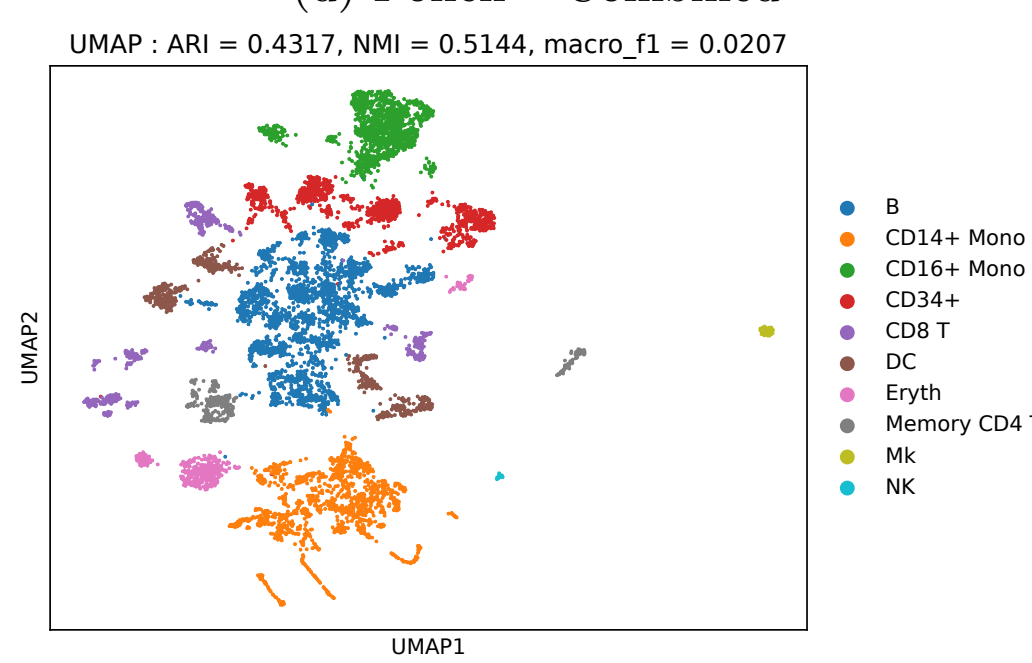

(f) CBMC – Combined

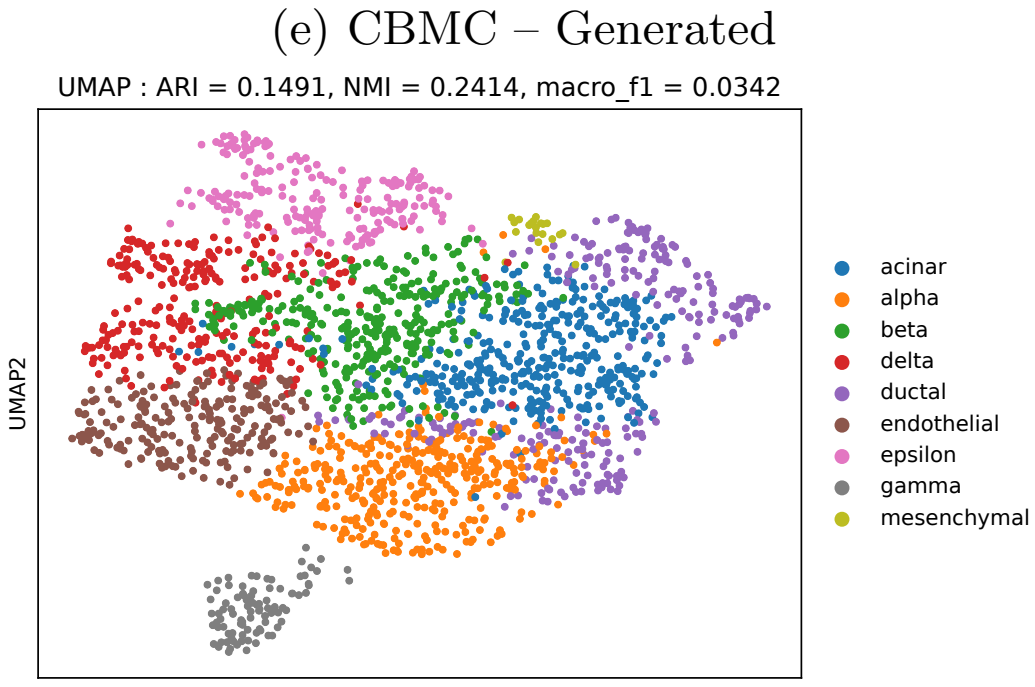

(g) Muraro – Generated

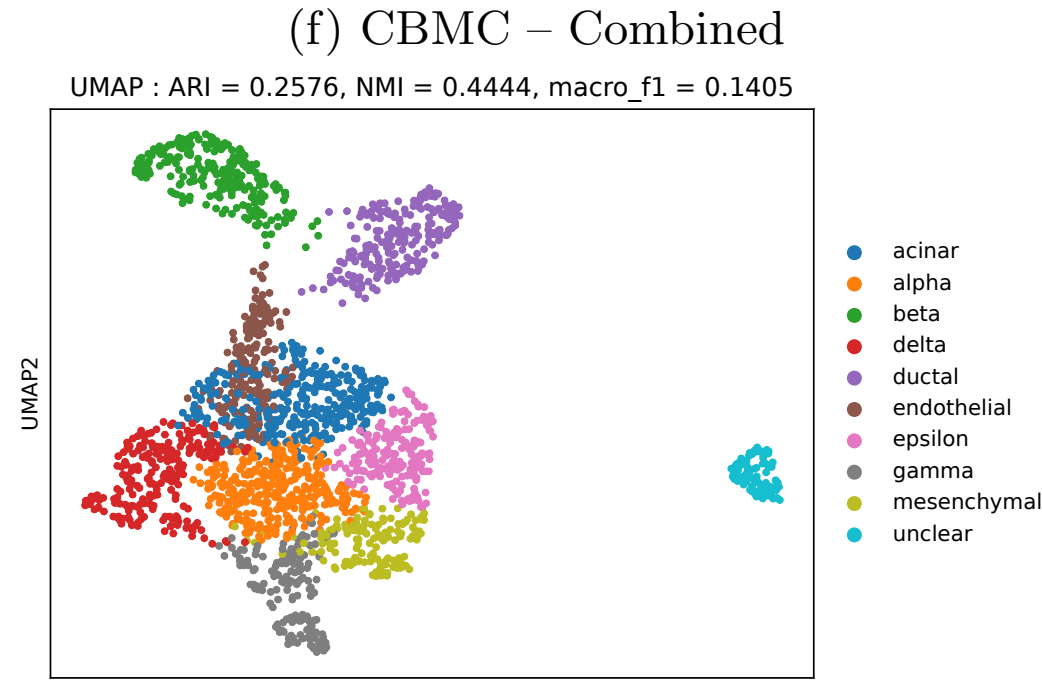

(h) Muraro – Combined

Fig 30: GAN results for generated(left) and combined(right) dataset across Yan, Pollen, CBMC, and Muraro for feature selection using CV2.

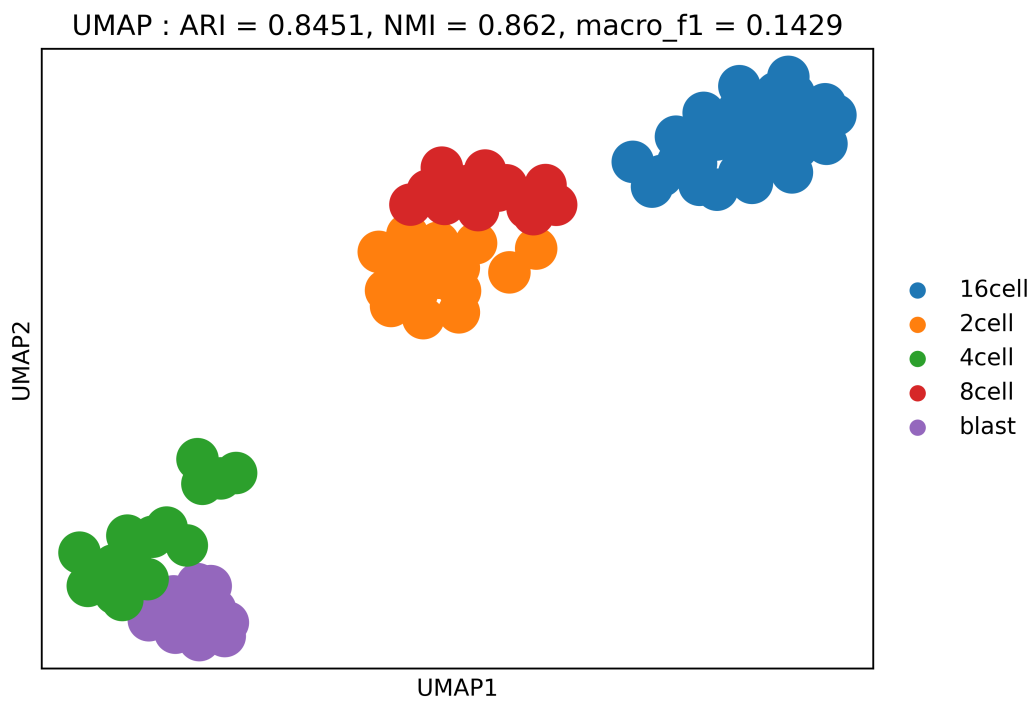

(a) Yan – Generated

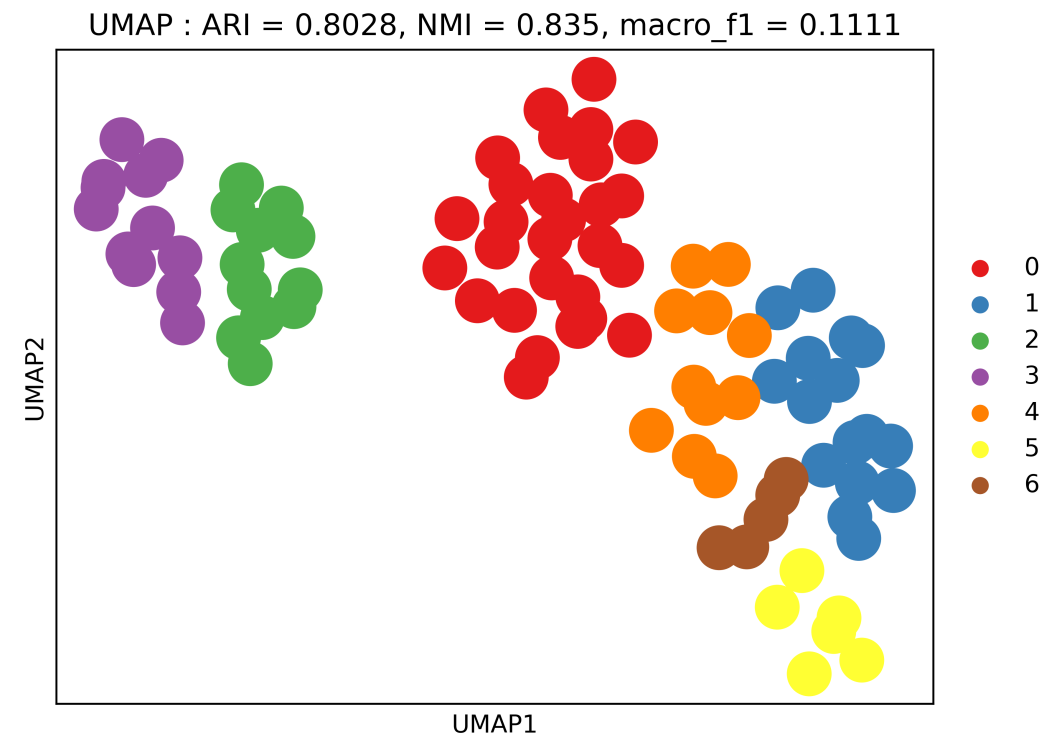

(b) Yan – Combined

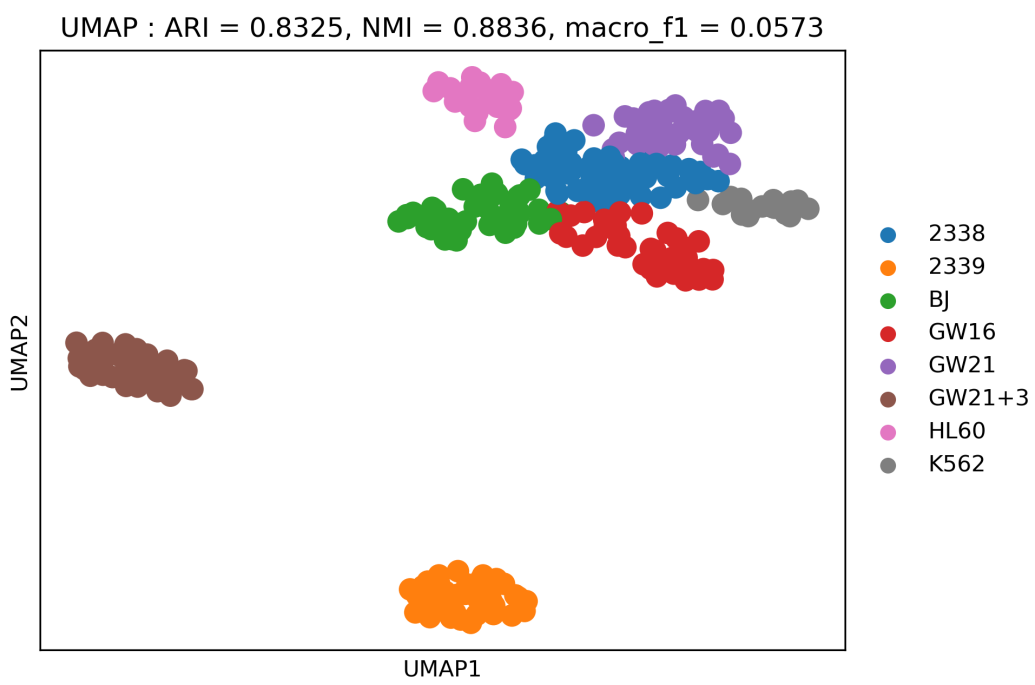

(c) Pollen – Generated

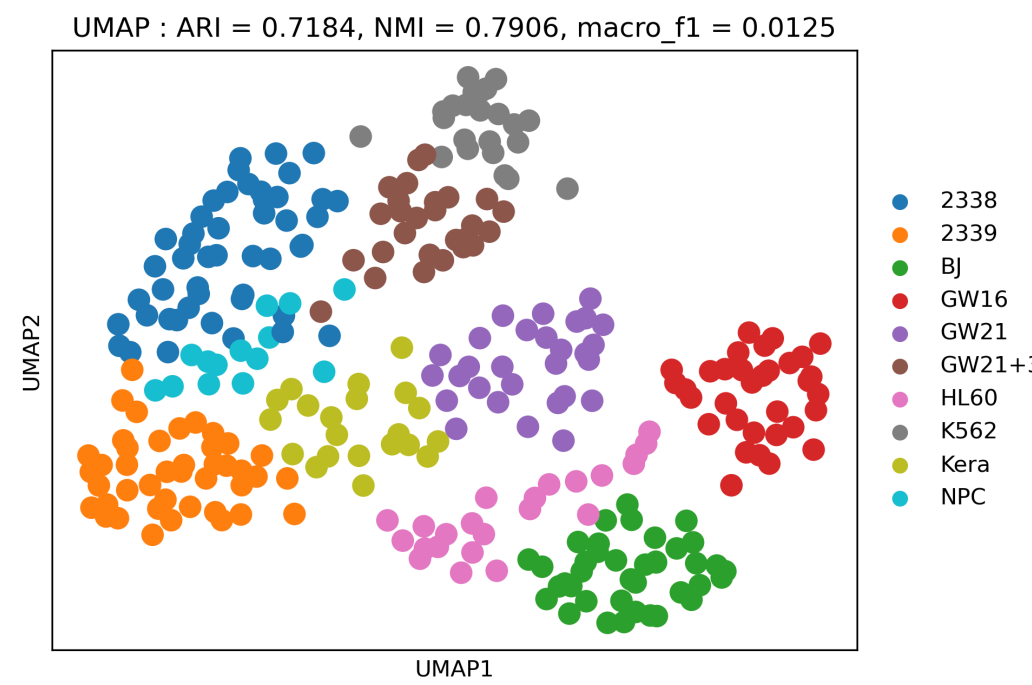

(d) Pollen – Combined

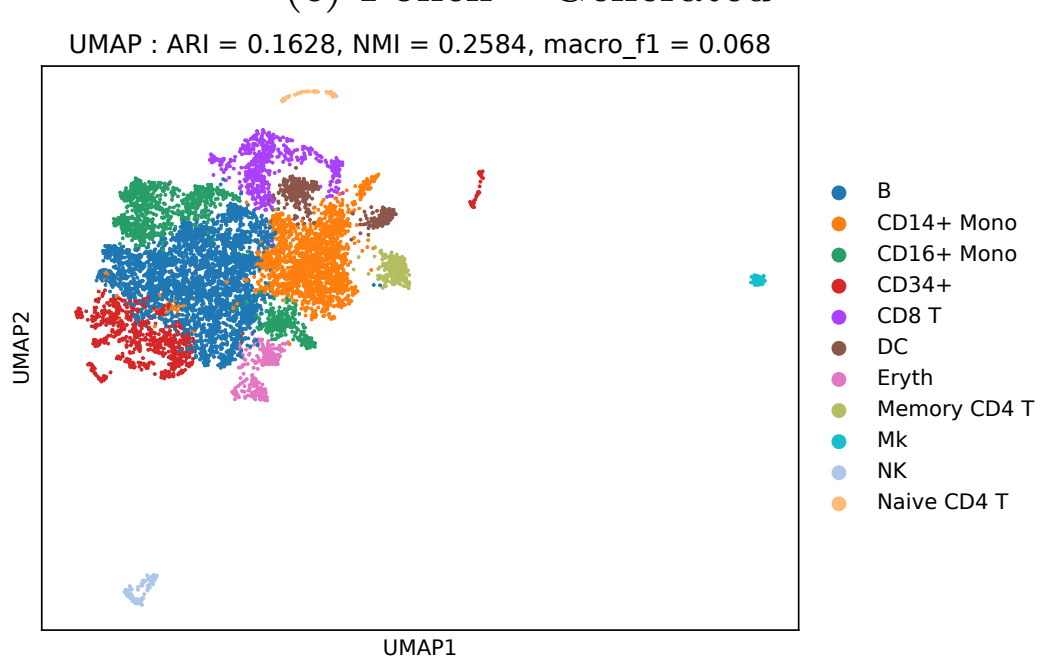

(e) CBMC – Generated

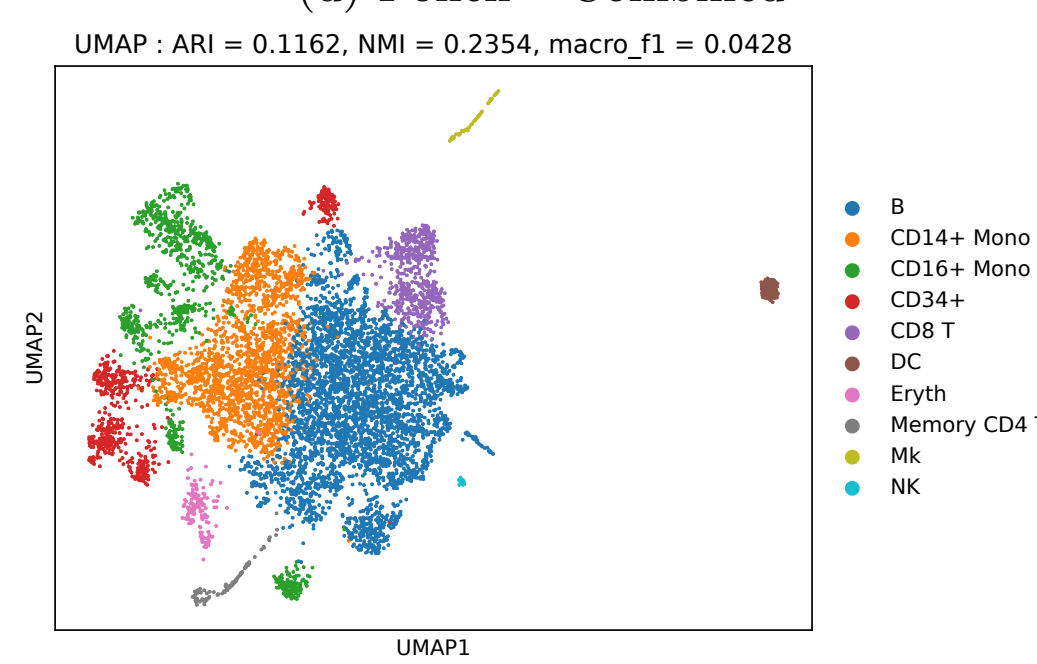

(f) CBMC – Combined

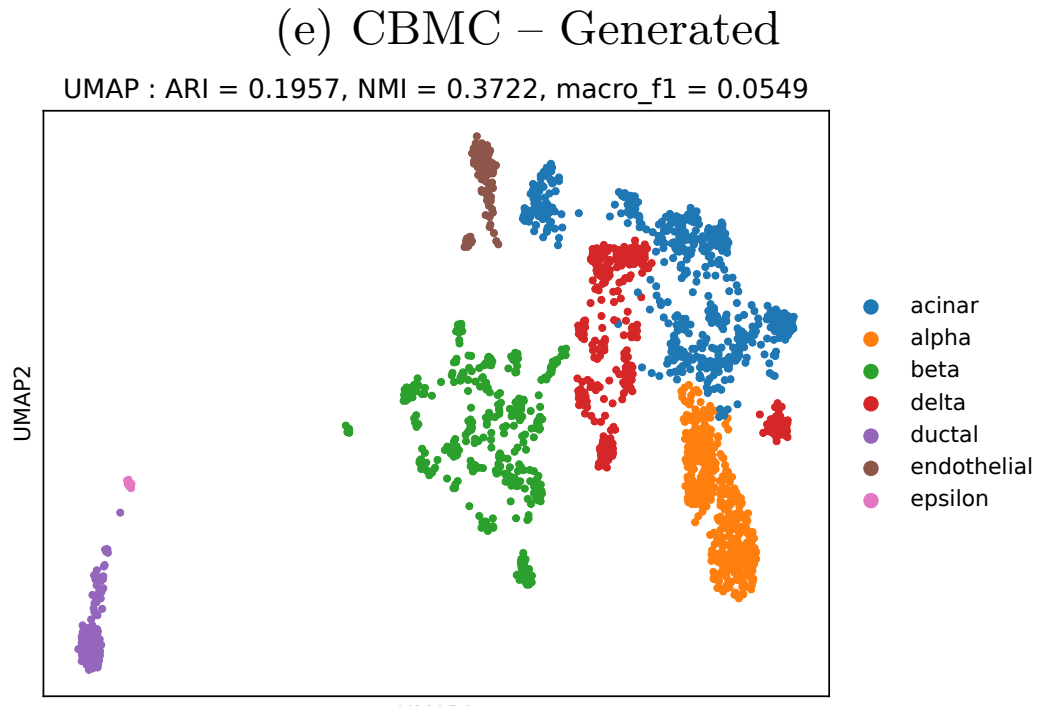

(g) Muraro – Generated

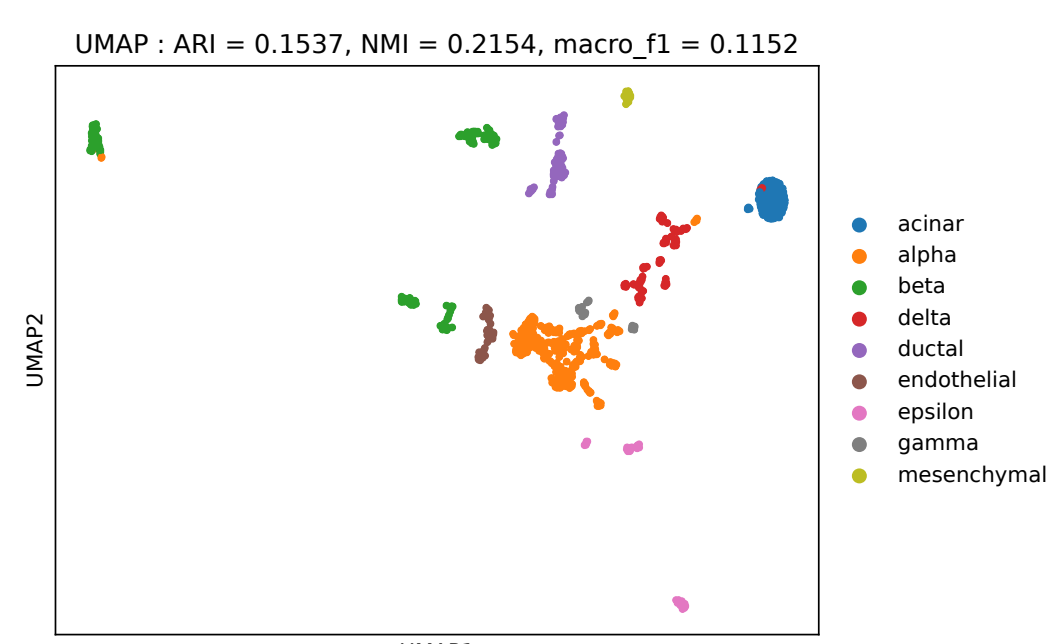

(h) Muraro – Combined

Fig 31: LSH results for generated(left) and combined(right) dataset across Yan, Pollen, CBMC, and Muraro for feature selection using Fano index.

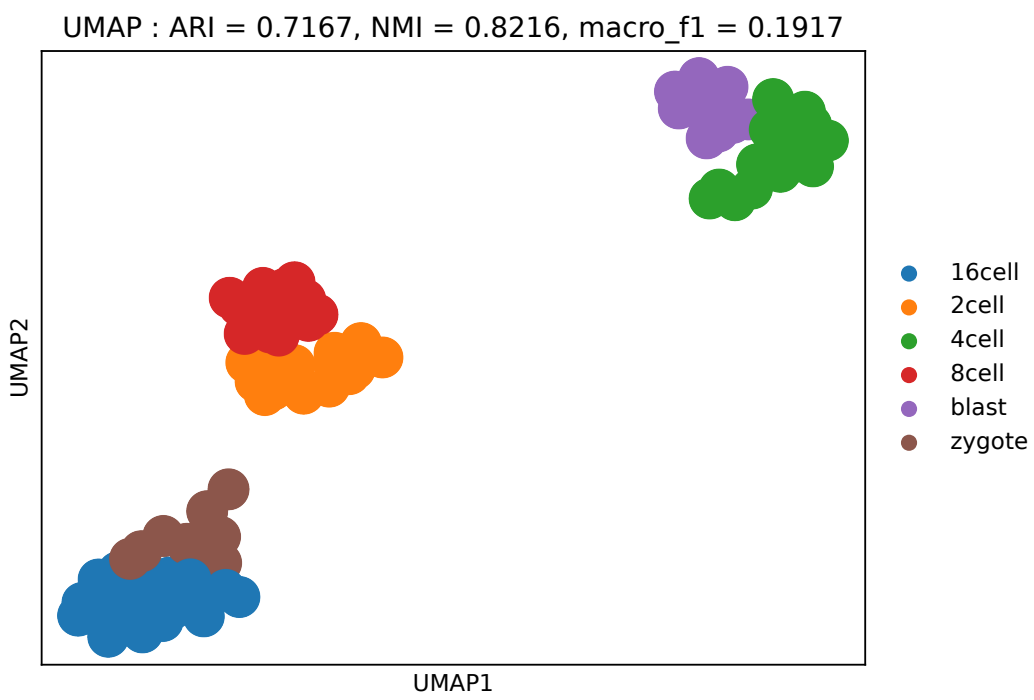

(a) Yan – Generated

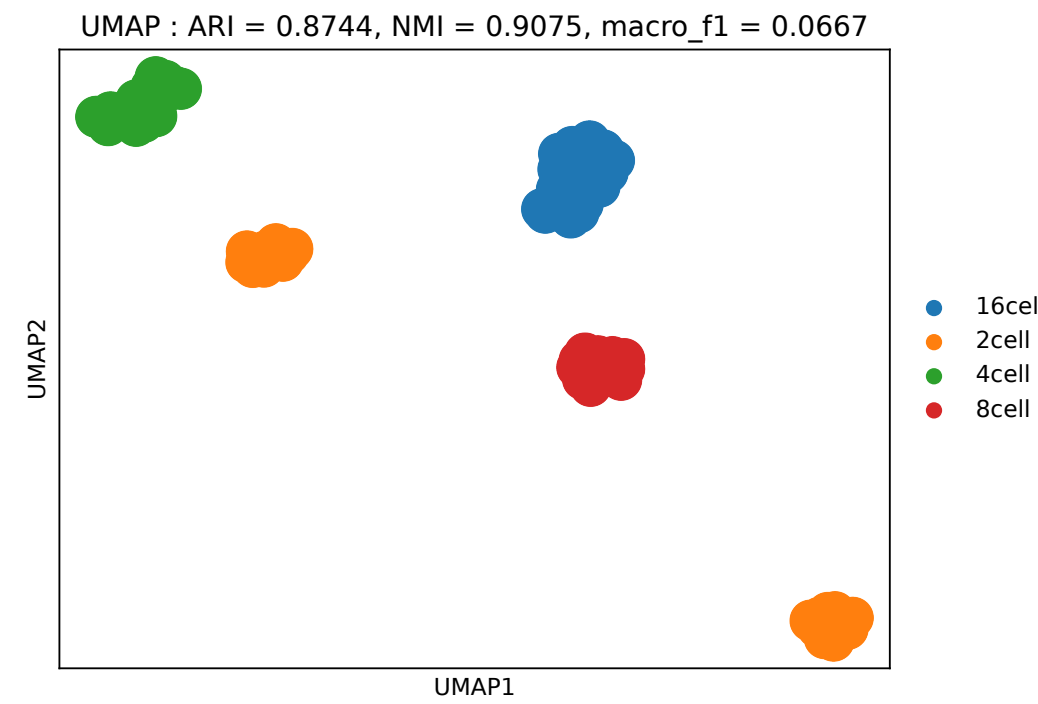

(b) Yan – Combined

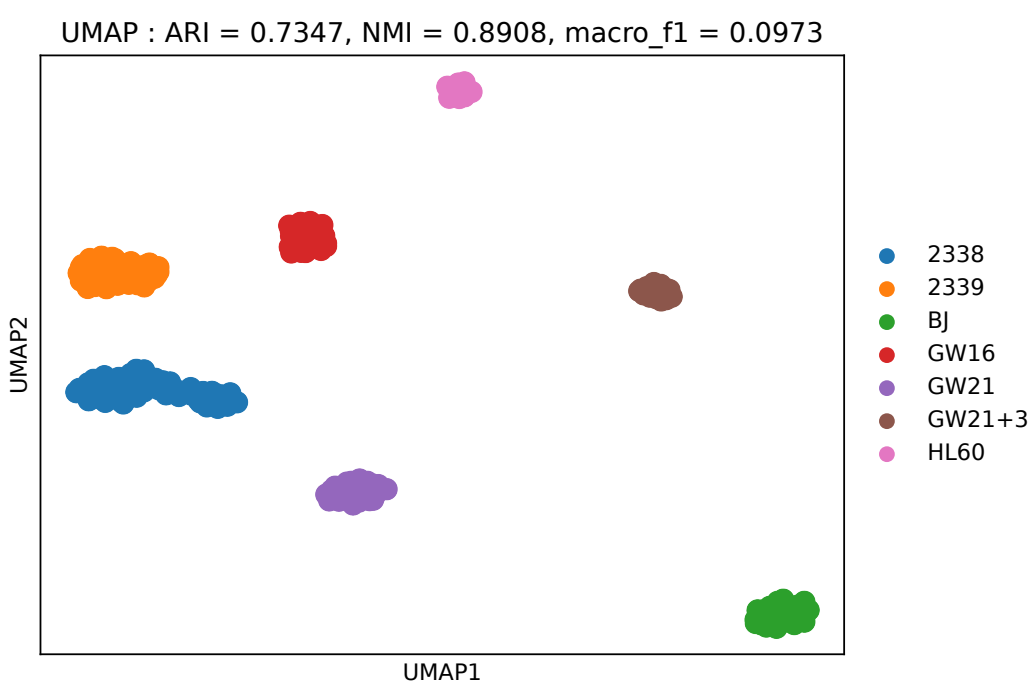

(c) Pollen – Generated

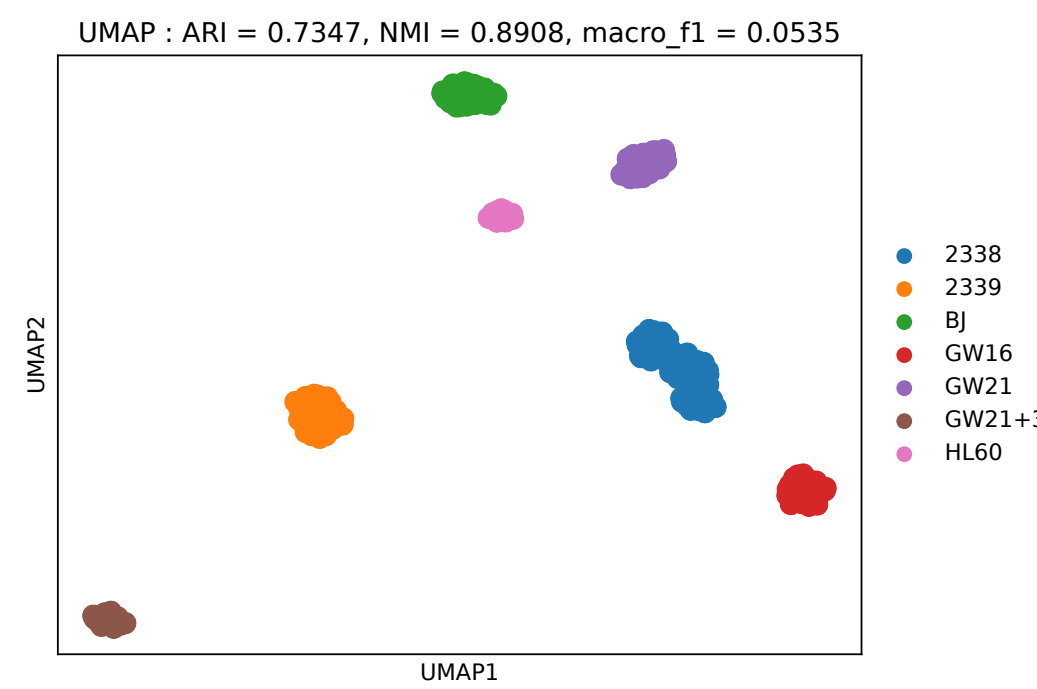

(d) Pollen – Combined

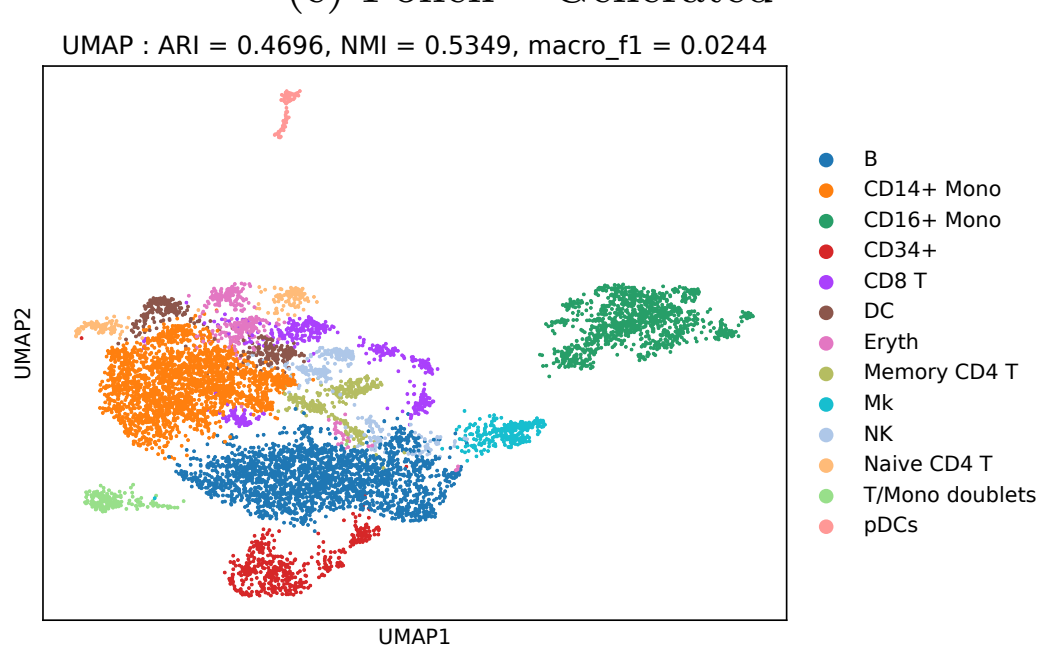

(e) CBMC – Generated

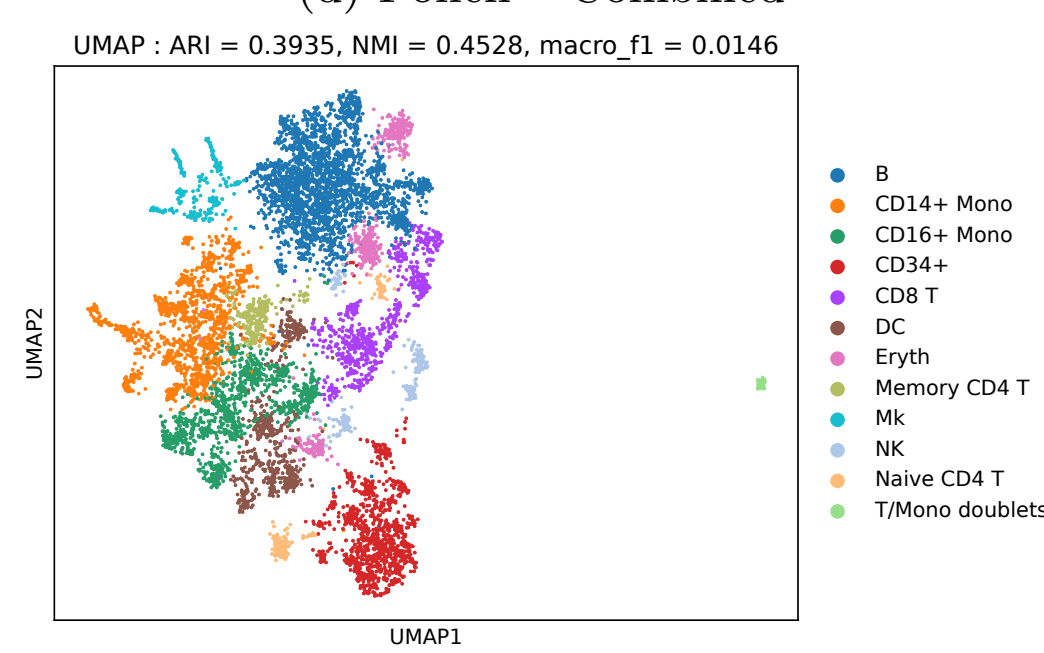

(f) CBMC – Combined

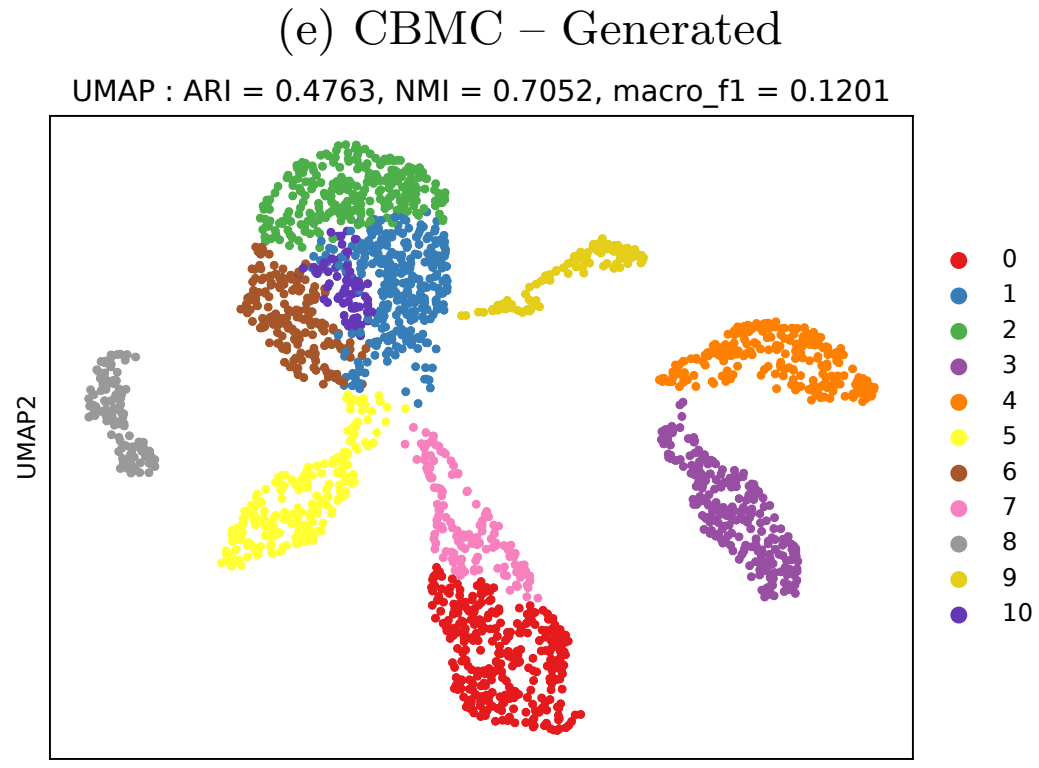

(g) Muraro – Generated

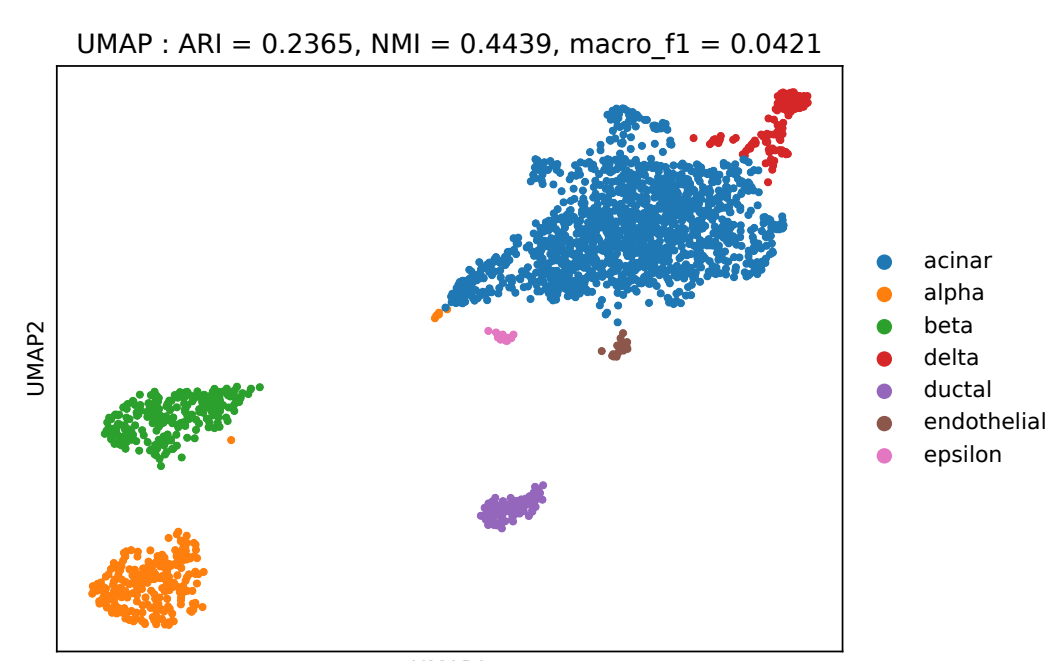

(h) Muraro – Combined

Fig 32: LSH results for generated(left) and combined(right) dataset across Yan, Pollen, CBMC, and Muraro for feature selection using CV2.

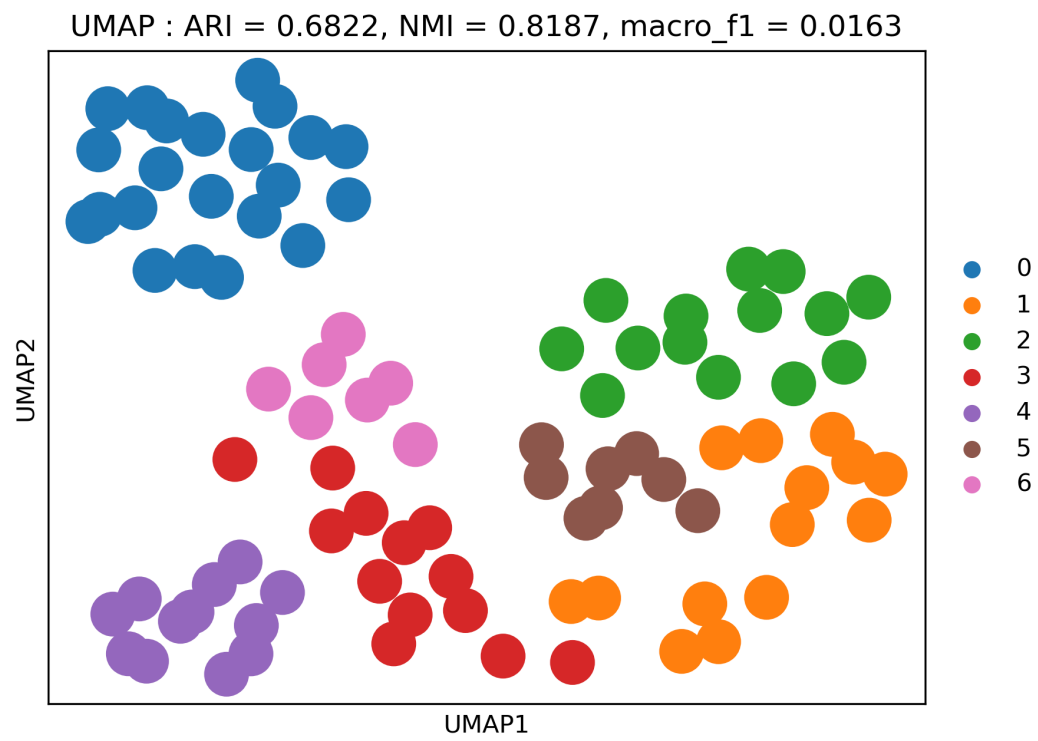

(a) Yan – Generated

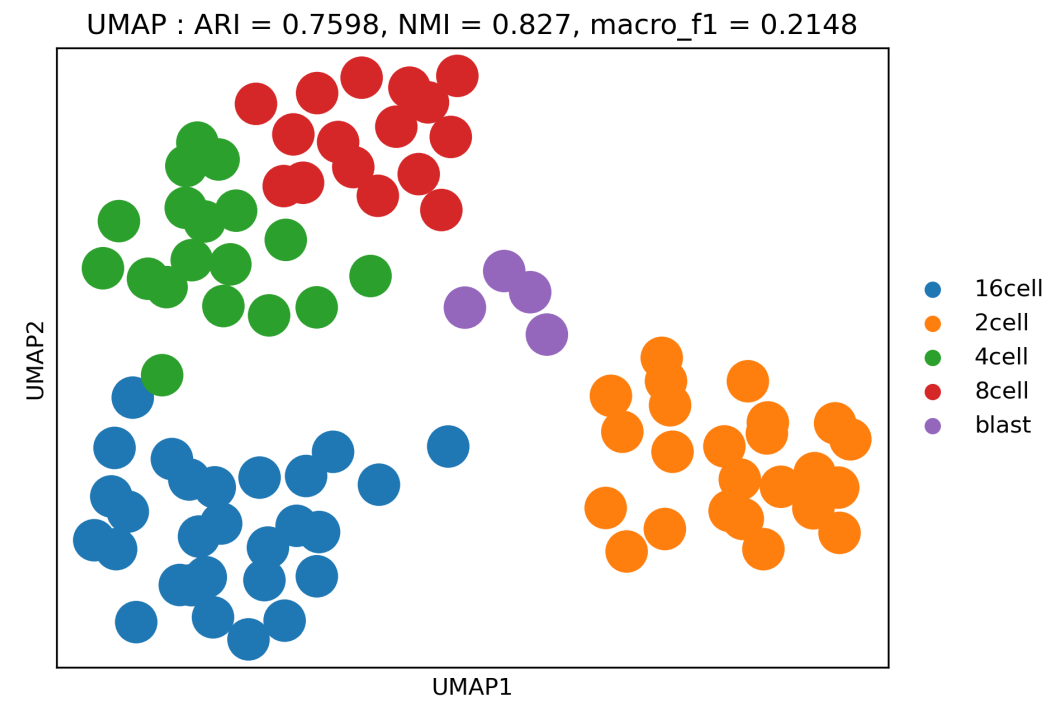

(b) Yan – Combined

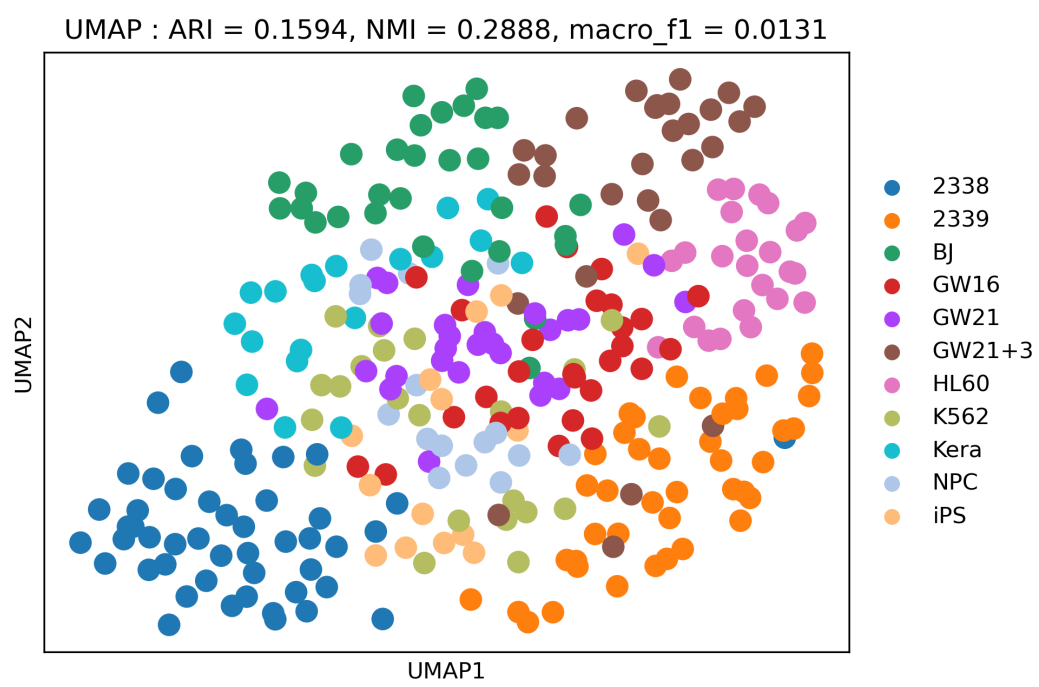

(c) Pollen – Generated

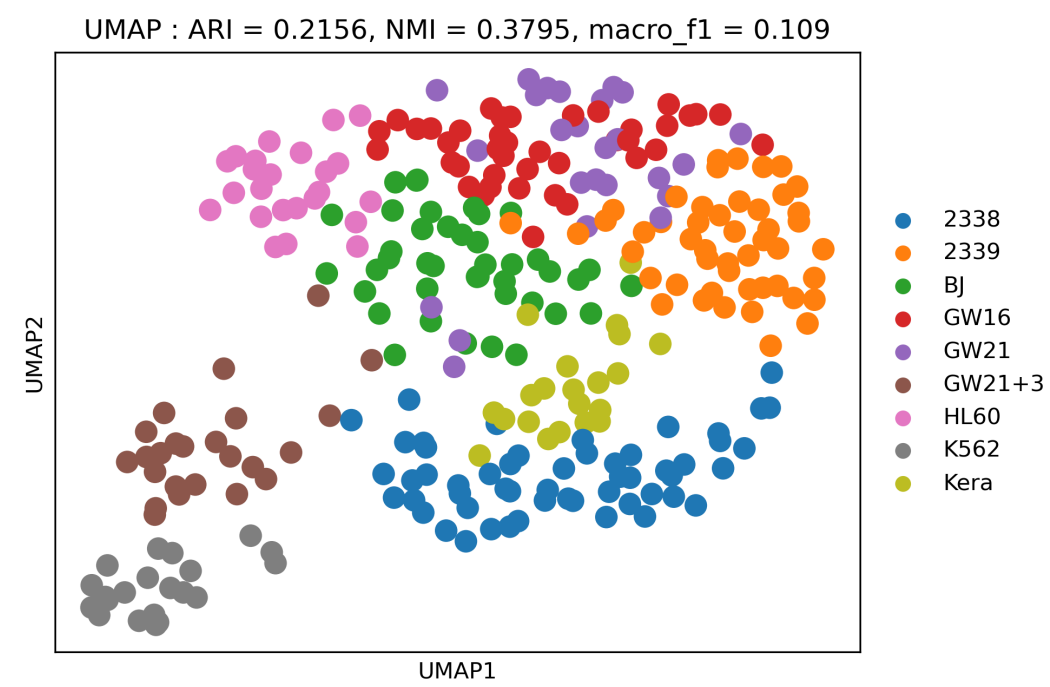

(d) Pollen – Combined

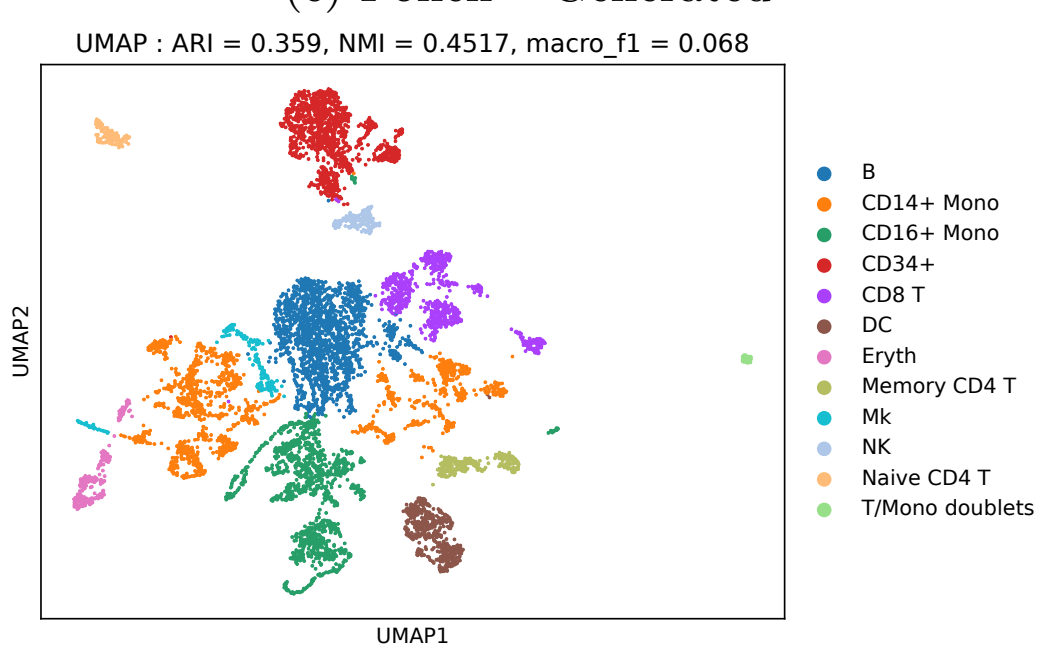

(e) CBMC – Generated

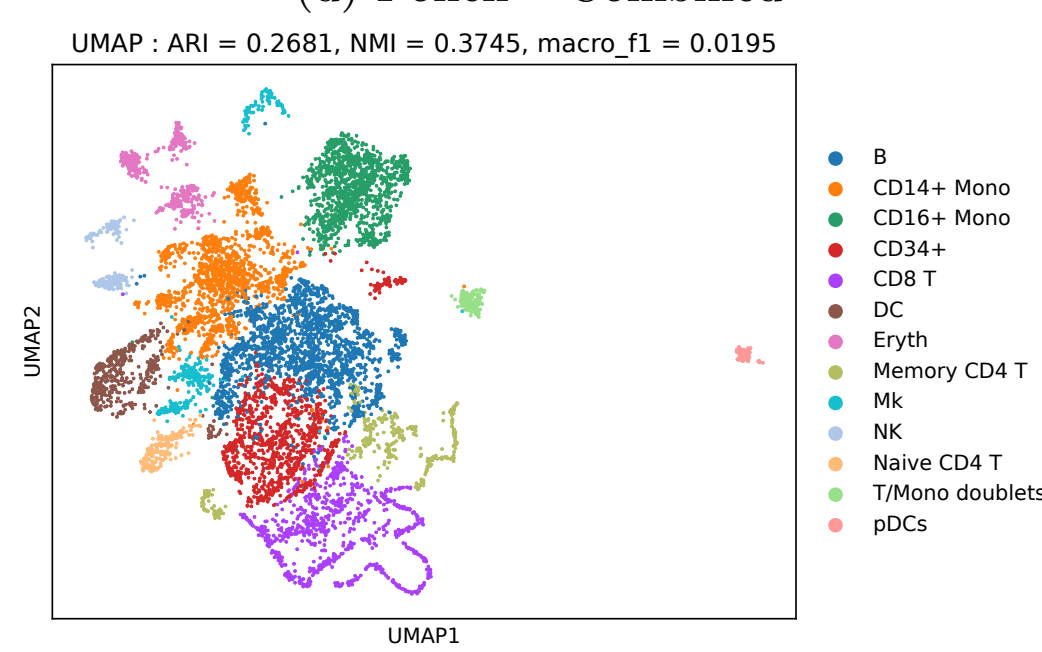

(f) CBMC – Combined

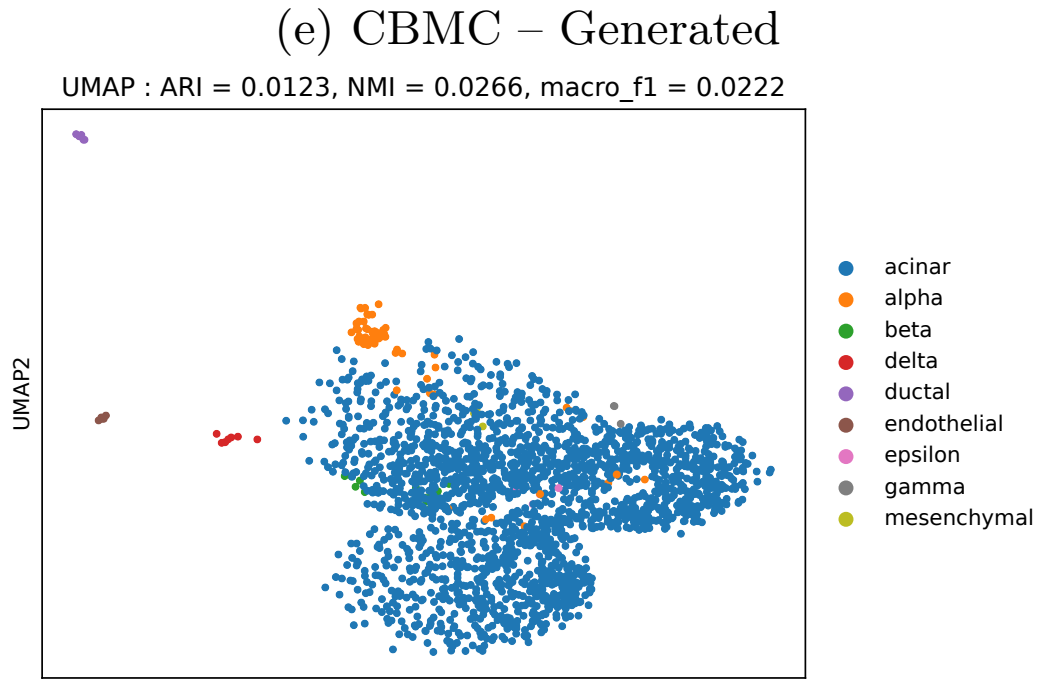

(g) Muraro – Generated

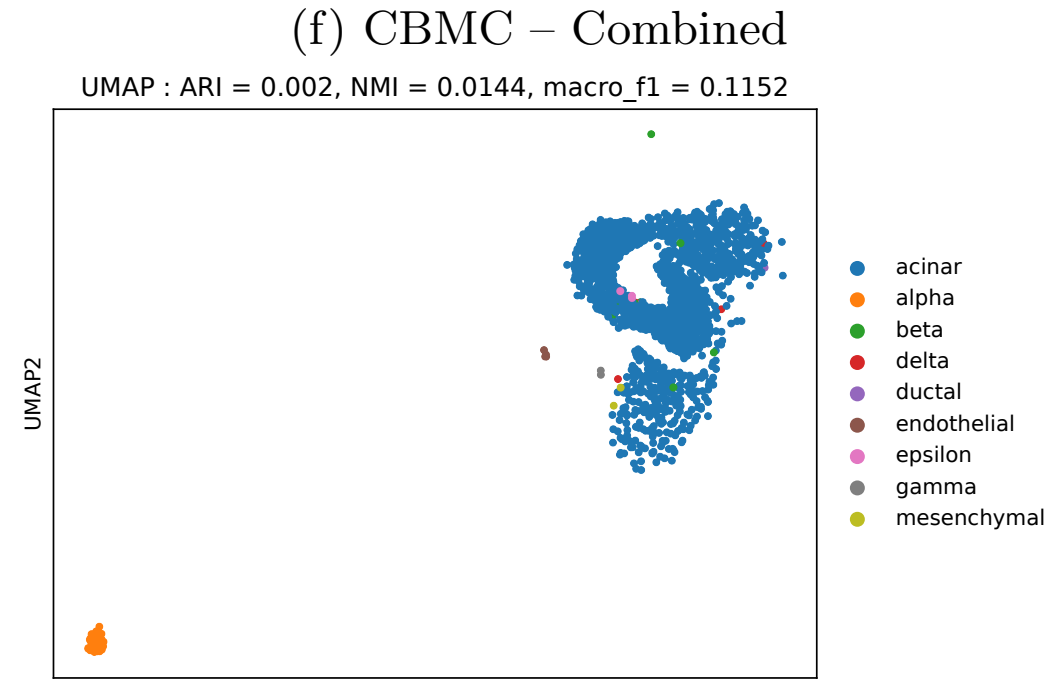

(h) Muraro – Combined

Fig 33: VAE results for generated(left) and combined(right) dataset across Yan, Pollen, CBMC, and Muraro for feature selection using Fano index.

Table 8: Dataset-specific fixed marker-gene sets used for clustering evaluation. Marker genes were selected independently of generated data.

Dataset	Cell type	Marker genes	Selection method
Yan	zygote	DPPA3, NLRP5, GDF9, HSF1	Literature-backed maternal legacy markers
Yan	2cell	DPRX, PRAMEF1, ZNF281, KDM4A	Literature-backed early-cleavage stage markers
Yan	4cell	DUX4, LEUTX, ZSCAN4, CPHX1	Literature-backed minor genome activation markers
Yan	8cell	DPPA2, KHDC3L, POU5F1, NANOG	Literature-backed major genome activation markers
Yan	16cell	GATA3, CLDN19, POU5F1, CDX2	Literature-backed morula stage compaction markers
Yan	blast	NANOG, CDX2, GATA6, KRT18	Literature-backed blastocyst lineage specification markers
Pollen	HL60	MPO, CD33, LYZ, ITGAM	Literature-backed myeloid lineage markers
Pollen	K562	GYPA, HBG1, HBG2, TFRC	Literature-backed erythroid lineage markers
Pollen	Kera	KRT14, KRT5, KRT10, KRT1	Literature-backed skin epidermal markers
Pollen	BJ	COL1A1, COL1A2, FN1, ACTA2	Literature-backed skin dermal fibroblast markers
Pollen	GW16	VIM, SOX2, PAX6, SLC1A3	Literature-backed cortical progenitor & radial glia markers
Pollen	iPS	POU5F1, NANOG, TDGF1, SOX2	Literature-backed stem cell pluripotency markers
Pollen	2338	EPCAM, KRT8, KRT18, CDH1	Literature-backed epithelial breast carcinoma markers
Pollen	2339	CD19, MS4A1, CD79A, CD79B	Literature-backed B-lymphoblastoid cell markers
Pollen	GW21+3	SNAP25, GRIA2, CAMKV, ADRA2A	Literature-backed maturing cortical neuron markers
Pollen	NPC	NES, PAX6, SOX1, SOX2	Literature-backed neural progenitor lineage markers
Pollen	GW21	DCX, STMN2, NEUROD6, TBR1	Literature-backed cortical neuron migration markers
CBMC	Eryth	HBB, HBA1, HBA2, GYPA, AHSP, ALAS2, SLC4A1, KLF1	Literature-based blood cell markers
CBMC	NK	NKG7, GNLY, KLRF1, PRF1, GZMB, KLRD1	Literature-based blood cell markers
CBMC	CD14+ Mono	CD14, S100A8, S100A9, CST3, FCN1	Literature-based blood cell markers
CBMC	CD16+ Mono	FCGR3A, CDKN1C	Literature-based blood cell markers
CBMC	CD34+	CD34, KIT, PROM1	Literature-based blood cell markers
CBMC	CD8 T	CD8A, CD8B, GZMK	Literature-based blood cell markers
CBMC	Memory CD4 T	S100A4, CD4, IL7R, CD44	Literature-based blood cell markers
CBMC	Naive CD4 T	CCR7, SELL, LEF1, TCF7	Literature-based blood cell markers
CBMC	B	CD19, MS4A1, CD79A, CD79B, BANK1	Literature-based blood cell markers
CBMC	Mk	PPBP, PF4, ITGA2B, GP9, GP1BA	Literature-based blood cell markers
CBMC	pDCs	LILRA4, TCF4, CLEC4C, IRF7, IRF8	Literature-based blood cell markers
CBMC	DC	FCER1A, CLEC10A, FLT3	Literature-based blood cell markers
CBMC	T/Mono doublets	CD3D, CD3E, CD14, LYZ	Literature-based blood cell markers
Muraro	alpha	GCG, TTR, ARX, IRX2	Literature-based pancreatic cell markers
Muraro	beta	INS, IAPP, NKX6-1, MAFA, PDX1, DLK1	Literature-based pancreatic cell markers
Muraro	delta	SST, HHEX, RBP4, LEPR	Literature-based pancreatic cell markers
Muraro	ductal	KRT19, CFTR, SOX9, SPP1, MMP7, TFF1, TFF3	Literature-based pancreatic cell markers
Muraro	acinar	CPA1, PRSS1, CTRB1, CTRB2, CELA3A, AMY2A, REG1A, REG1B	Literature-based pancreatic cell markers
Muraro	endothelial	PECAM1, CDH5, VWF, CLDN5, ENG, KDR, PLVAP	Literature-based pancreatic cell markers
Muraro	gamma	PPY, PAX6	Literature-based pancreatic cell markers
Muraro	epsilon	GHRL	Literature-based pancreatic cell markers
Muraro	mesenchymal	COL1A1, COL1A2, PDGFRA, THY1, LUM, DCN	Literature-based pancreatic cell markers
Muraro	unclear	variance-selected	Literature-based pancreatic cell markers

Table 9: Clustering performance using fixed biological marker-gene sets. The same marker-gene set was used for all methods within each dataset.

**Dataset**	**Method**	**ARI**	**NMI**
Yan	GAN	0.0247	0.1813
Yan	VAE	0.1909	0.3089
Yan	LSH-GAN	0.0697	0.1658
Yan	GARAGE	0.4482	0.6747
Pollen	GAN	0.0801	0.1718
Pollen	VAE	0.0707	0.3462
Pollen	LSH-GAN	0.0148	0.0805
Pollen	GARAGE	0.7079	0.6096
CBMC	GAN	0.0573	0.1593
CBMC	VAE	0.1978	0.5401
CBMC	LSH-GAN	0.0094	0.0351
CBMC	GARAGE	0.2843	0.3986
Muraro	GAN	0.006	0.0157
Muraro	VAE	0.0963	0.407
Muraro	LSH-GAN	0.1052	0.211
Muraro	GARAGE	0.0684	0.2073

7.7 Training-stability ablation

To evaluate whether attention-guided leakage improves adversarial training stability, we compared the no-leakage GAN baseline ($\lambda = 0$) with GARAGE under leakage settings $\lambda \in \{0.10, 0.20, 0.30\}$. All models used the same preprocessing pipeline, generator architecture, discriminator architecture, optimizer settings, and training schedule. The only difference among the compared settings was the construction of the generator input batch. For $\lambda = 0$, the generator received only random noise vectors. For GARAGE, the generator received a batch-level mixture of random noise vectors and GAT-prioritized seed cells.

For each dataset and leakage setting, we monitored the generator loss and discriminator loss across training iterations (see table 10 and figure 34). To summarize training stability quantitatively, we computed the mean final generator loss, mean final discriminator loss, and the standard deviation of each loss over the final training window. The loss standard deviation was used as a measure of oscillation, where lower values indicate more stable adversarial training. We also computed the final distributional distance between real and generated cells to assess whether improved loss stability was accompanied by better agreement between the real and synthetic transcriptomic distributions.

For a loss trajectory $\mathcal{L}(t)$ over training iterations, the loss oscillation over the final window $\mathcal{T}_{\text{final}}$ was computed as

$$\text{Oscillation}(\mathcal{L}) = \text{Std}\left(\{\mathcal{L}(t) : t \in \mathcal{T}_{\text{final}}\}\right).$$

This was computed separately for the generator and discriminator losses. Lower oscillation values indicate smoother training dynamics.

Table 10: Training-stability ablation comparing no-leakage GAN ($\lambda = 0$) with GARAGE under different leakage levels. Reported values are the mean and standard deviation of generator and discriminator losses across runs.

**Dataset**	**Leakage λ**	**g_loss** (mean)	**g_loss** (std)	**d_loss** (mean)	**d_loss** (std)
Yan	0.00	1.8084	0.4297	0.7825	0.0586
Yan	0.10	1.5196	0.3040	0.8264	0.0771
Yan	0.20	1.7311	0.5025	0.9632	0.4192
Yan	0.30	1.7521	0.5525	0.9842	0.4322
Pollen	0.00	2.1195	0.3206	0.6954	0.1193
Pollen	0.10	2.1611	0.4556	0.7397	0.1213
Pollen	0.20	2.0689	0.3100	0.7382	0.0625
Pollen	0.30	2.0954	0.2305	0.7984	0.2190
CBMC	0.00	2.0720	0.1923	0.6700	0.0085
CBMC	0.10	2.0931	0.3996	0.7022	0.0204
CBMC	0.20	2.0553	0.4461	0.7603	0.0489
CBMC	0.30	2.0358	0.4181	0.7201	0.0979
Muraro	0.00	1.7896	0.6483	0.8181	0.4357
Muraro	0.10	2.0384	0.3353	0.7381	0.1468
Muraro	0.20	2.0357	0.3928	0.7463	0.1544
Muraro	0.30	2.0927	0.2919	0.9287	0.4933

Overall, this ablation was designed to determine whether the attention-guided leakage mechanism stabilizes adversarial learning beyond the conventional noise-driven GAN setting. The comparison isolates the role of leakage because all other architectural and optimization settings were kept fixed across the no-leakage and leakage-based models.

7.8 Consolidated leakage-sensitivity analysis

To provide a clearer interpretation of the leakage hyperparameter λ, we consolidated the existing leakage-sensitivity results across all benchmark datasets. The goal of this analysis was to compare how changes in λ influence clustering performance and distributional agreement across datasets with

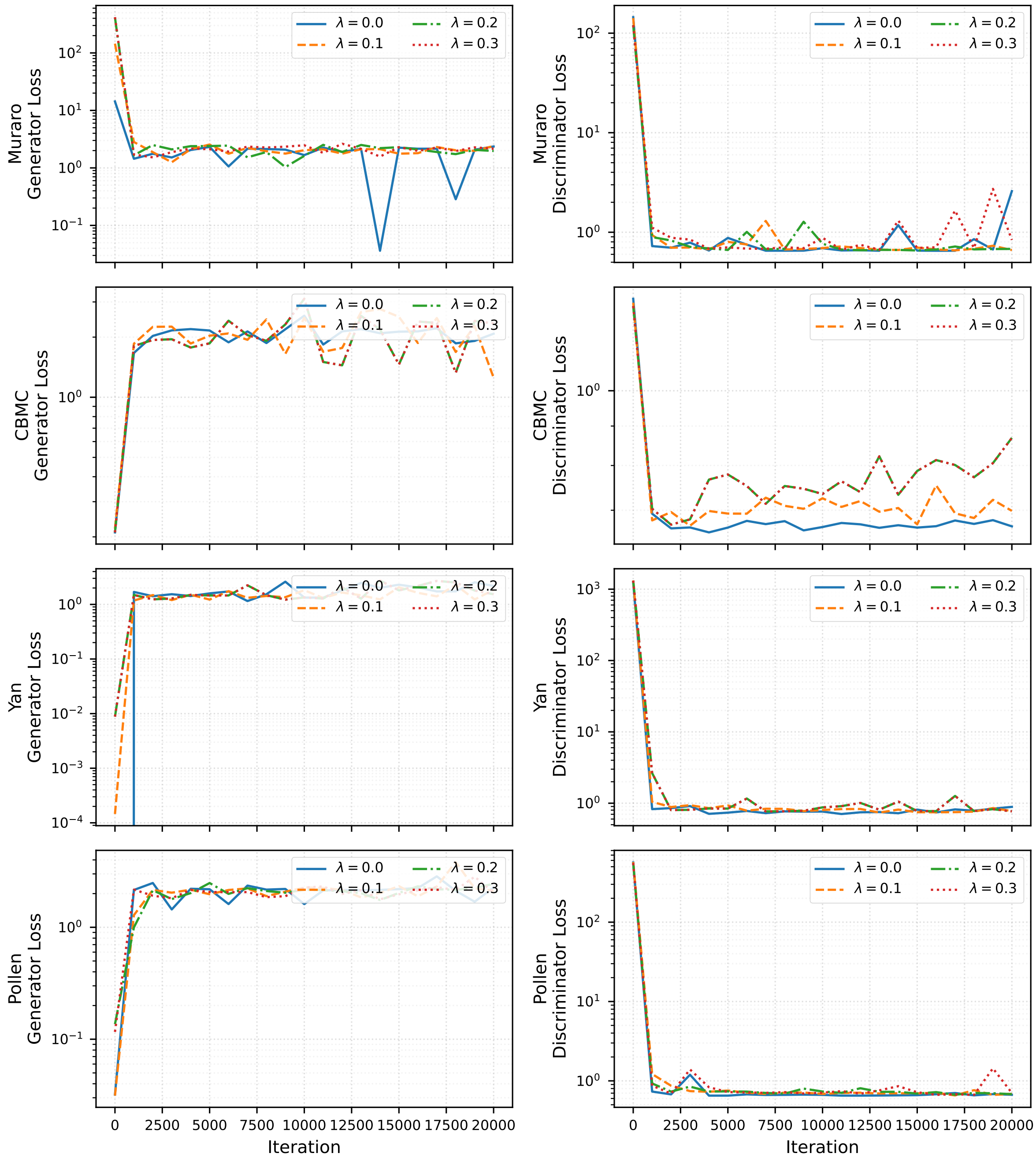

Fig 34: Generator and discriminator loss curves for the no-leakage GAN baseline ($\lambda = 0$) and GARAGE with attention-guided leakage. Curves summarize the training dynamics across independent runs, with shaded regions indicating the standard deviation where applicable.

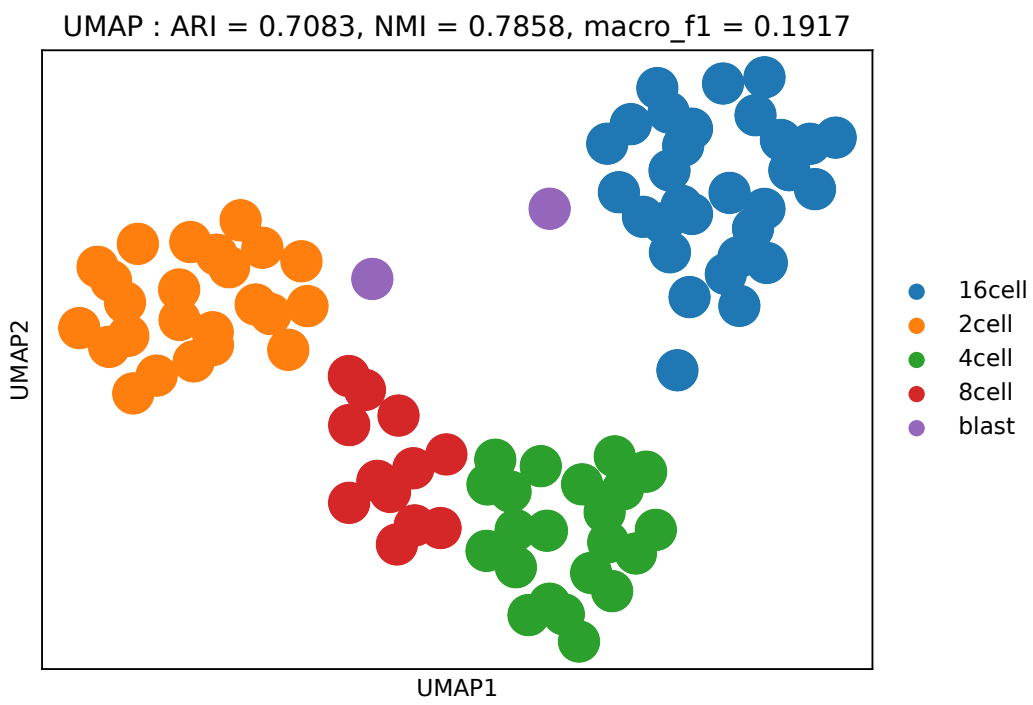

(a) Yan – Generated

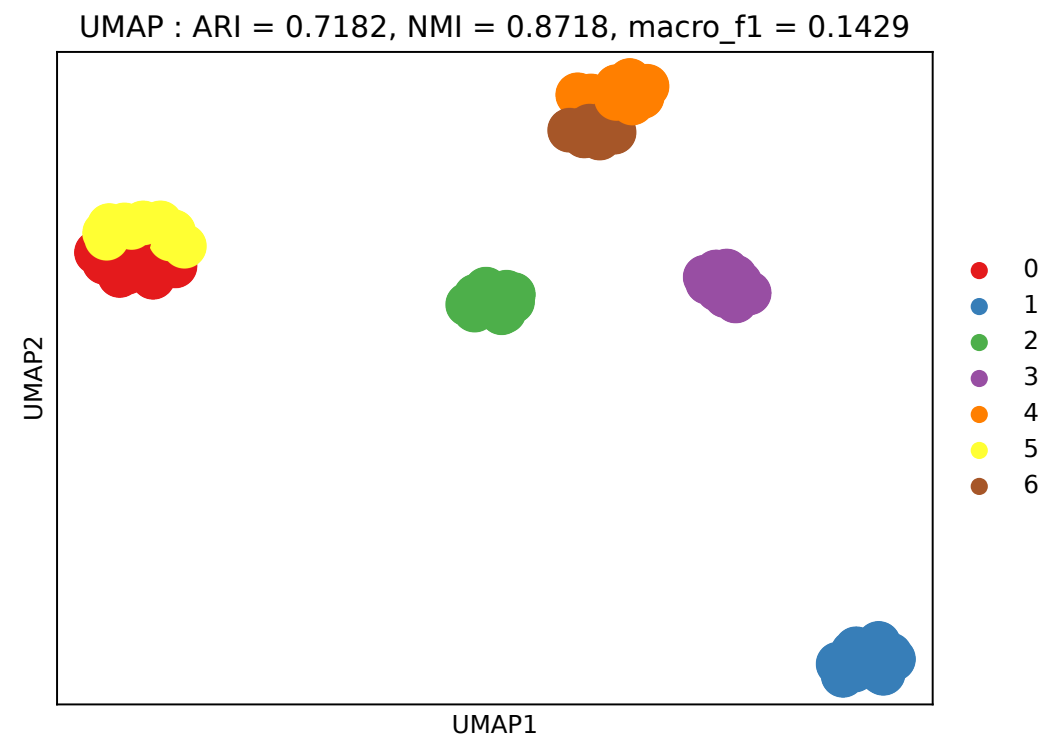

(b) Yan – Combined

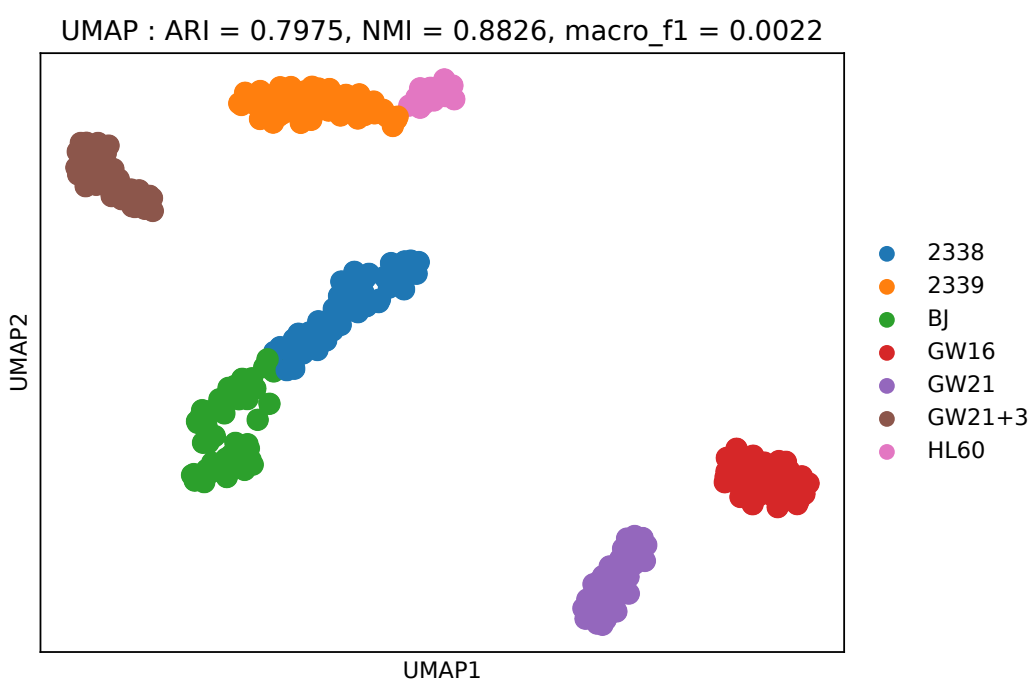

(c) Pollen – Generated

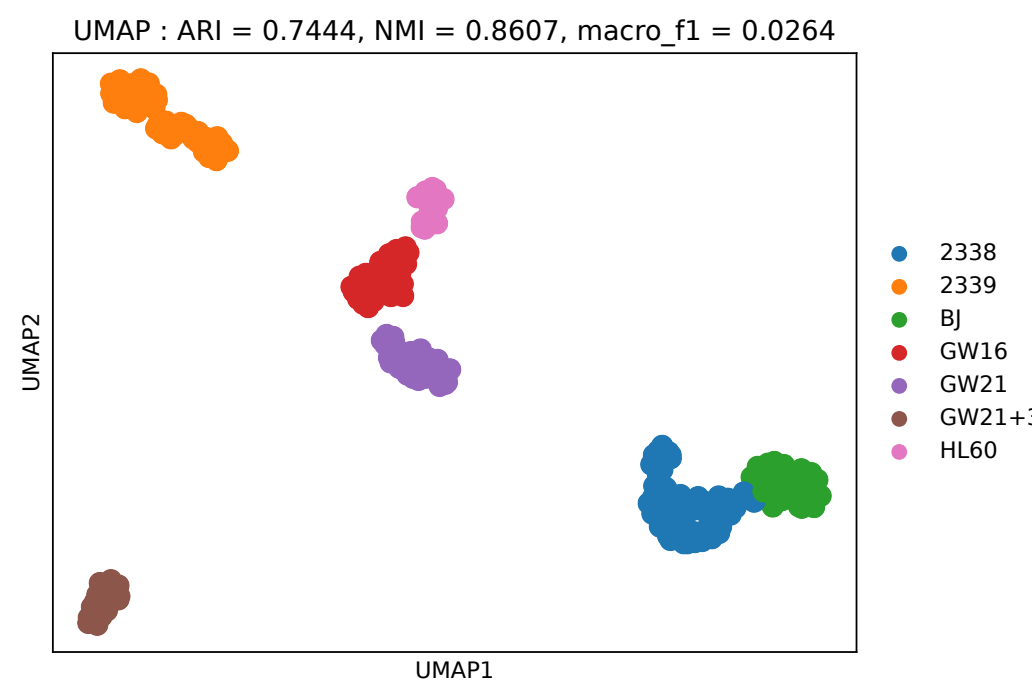

(d) Pollen – Combined

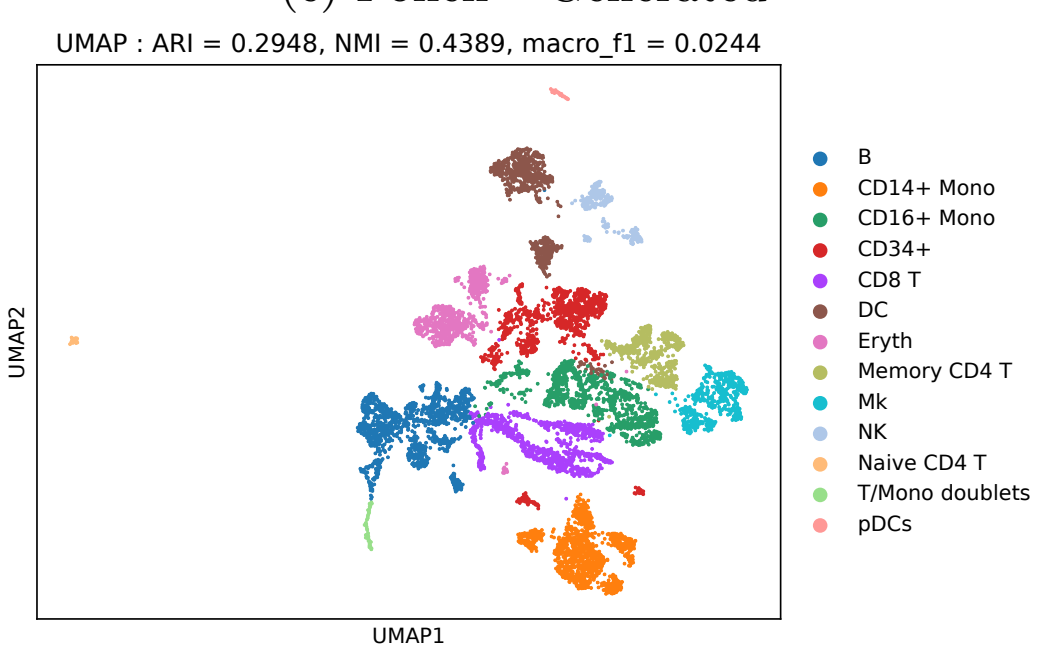

(e) CBMC – Generated

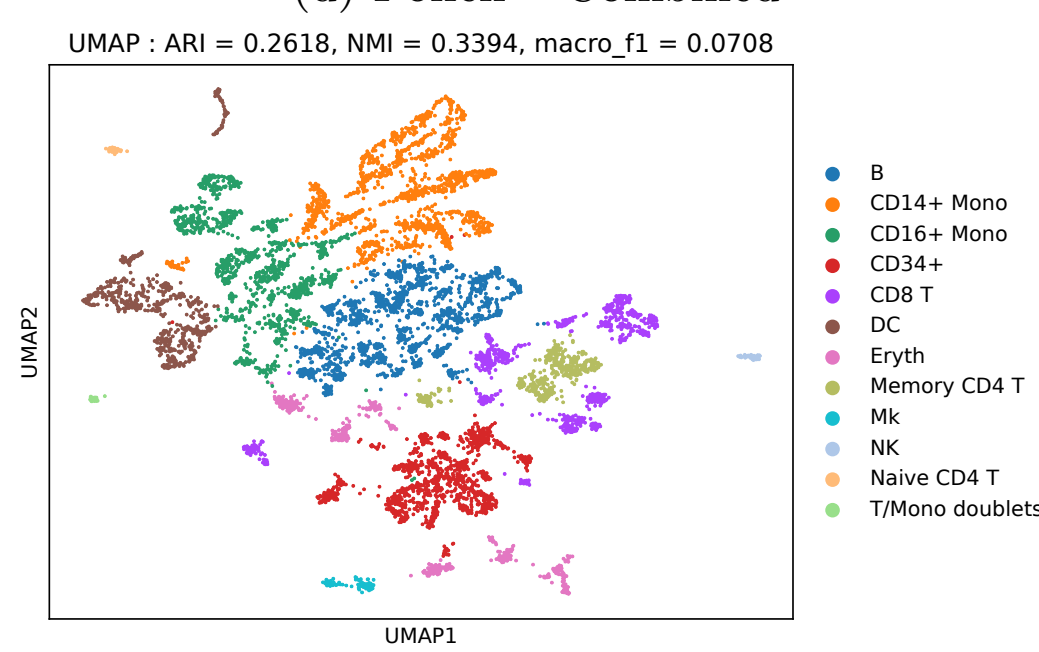

(f) CBMC – Combined

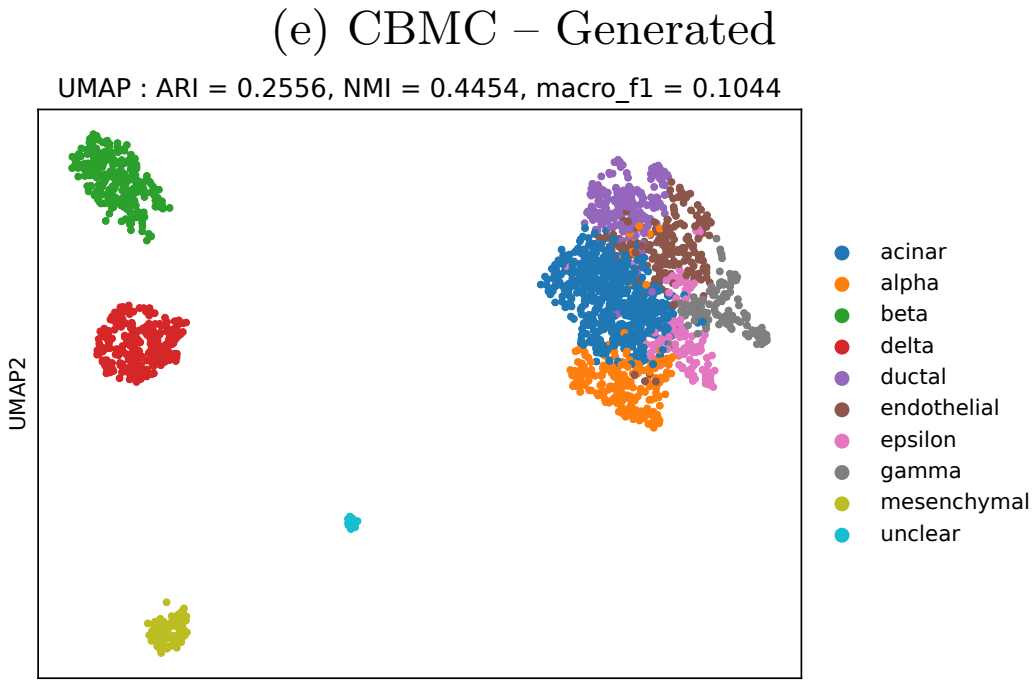

(g) Muraro – Generated

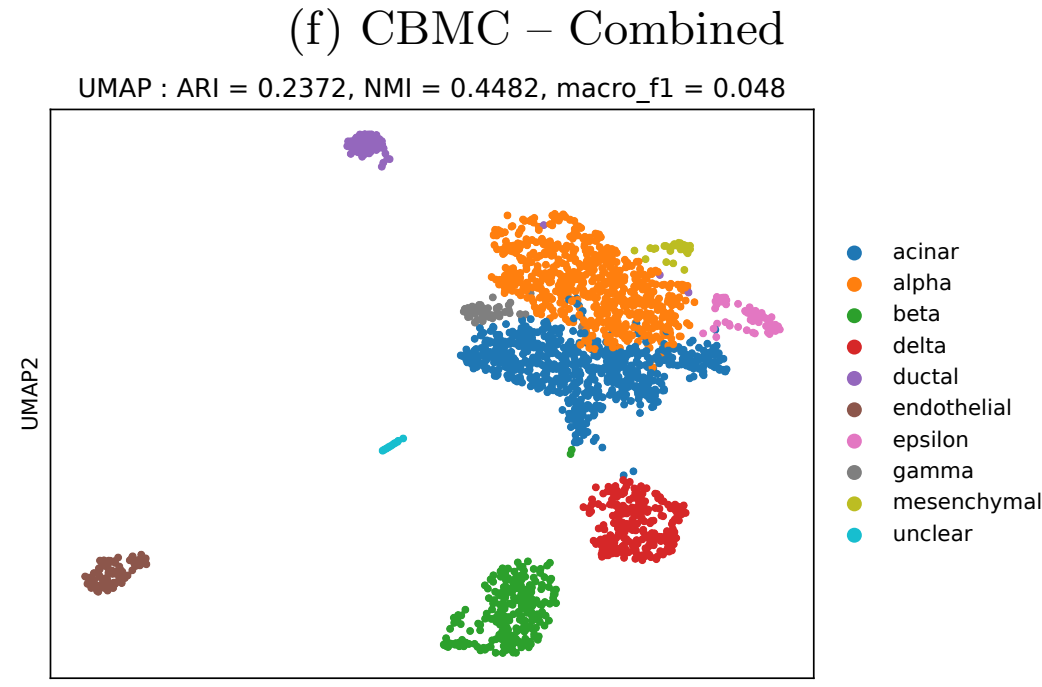

(h) Muraro – Combined

Fig 35: VAE results for generated(left) and combined(right) dataset across Yan, Pollen, CBMC, and Muraro for feature selection using CV2.

different levels of imbalance and transcriptomic complexity. The leakage values evaluated were $\lambda = 0.10$, $\lambda = 0.20$, and $\lambda = 0.30$.

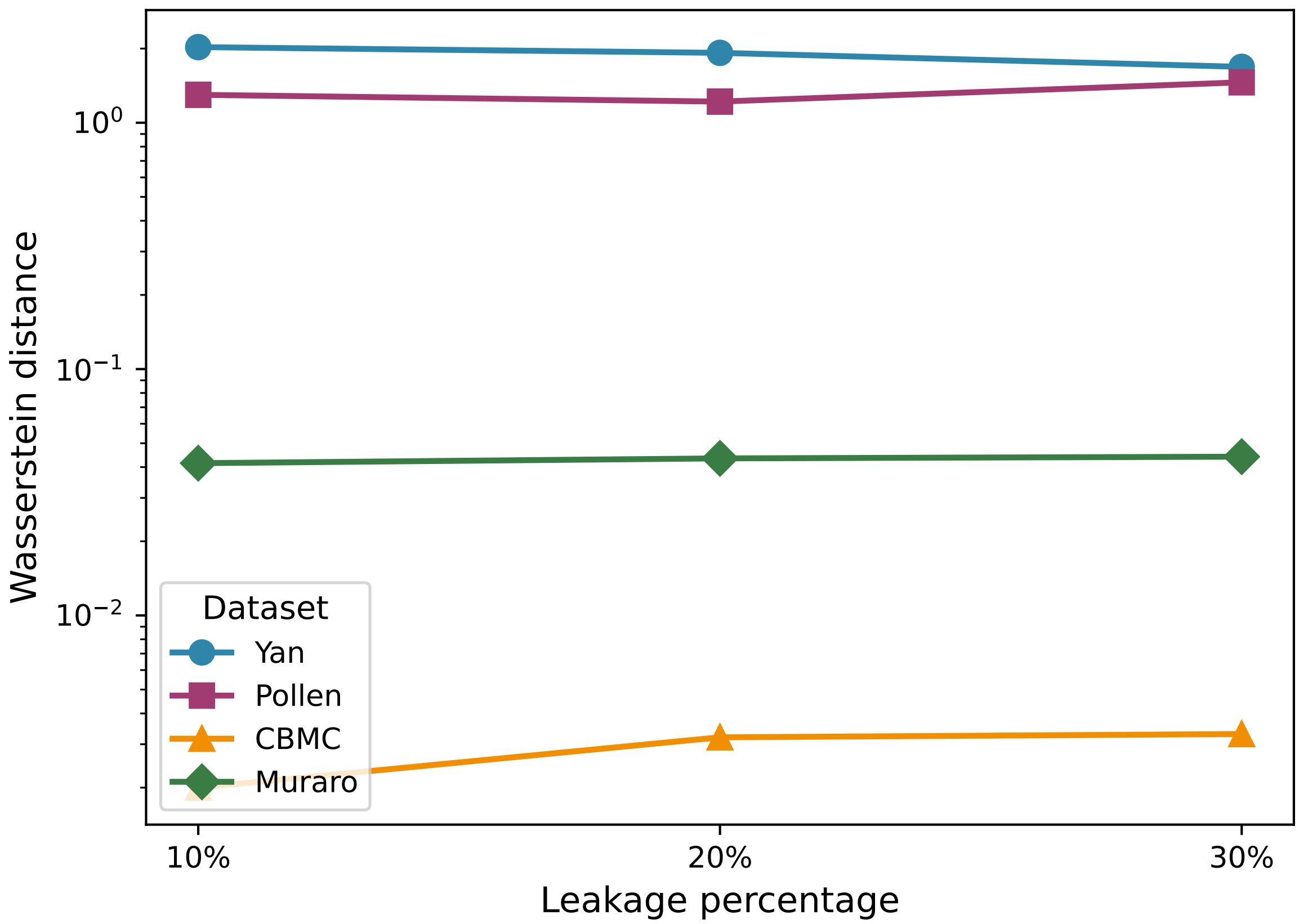

Fig 36: Consolidated Wasserstein distance (WD) trends across all benchmark datasets. Performance is shown as a function of the leakage parameter λ. The figure summarizes the dataset-specific response to increasing real-cell leakage and illustrates that the optimal leakage value is dataset-dependent.

Overall, the sensitivity analysis indicates that moderate leakage is generally sufficient for datasets with clearer subtype structure, whereas stronger leakage may help when rare-cell representation is limited or the manifold is more difficult to recover. However, very high leakage can reduce flexibility by anchoring the generator too strongly to selected real-cell examples. Therefore, λ should be selected by jointly considering downstream clustering metrics and label-agnostic distributional fidelity.

7.9 Biological validation of GAT-prioritized high-attention cells

To assess whether the GAT-prioritized cells correspond to biologically meaningful cell states, we performed a post hoc marker-gene validation using the CBMC dataset. After training the GAT module, cells were ranked according to their aggregated attention scores. The top-20% of cells were defined as the high-attention subset, and the bottom-20% of cells were defined as the low-attention subset. We then compared the expression of known immune and progenitor marker genes between these two subsets.

The marker panel contained 24 literature-supported marker genes representing eight CBMC-associated populations, including monocytes, NK cells, B cells, erythroid cells, megakaryocytes, hematopoietic progenitors, plasmacytoid dendritic cells, and conventional dendritic cells. For each marker gene, we computed the mean expression across all cells, the mean expression in high-attention cells, and the mean expression in low-attention cells. We also calculated the $\log_2$ fold-change between the high- and low-attention subsets and performed a Wilcoxon rank-sum test. The marker genes were not used during GARAGE model training; they were used only for post hoc biological interpretation of the attention-prioritized seed cells.

Table 11: Marker-gene validation of GAT-prioritised high-attention cells in the CBMC dataset.

Cell type	Gene	Marker class	Mean expr. (all)	Mean expr. (high)	Mean expr. (low)	$\log_2$FC	Wilcoxon p	Sig
CD14+ Mono	S100A8	Common	0.0232	0.1617	0.0109	3.8852	0.0228	*
CD14+ Mono	CD14	Common	0.0346	0.0998	0.0240	2.0532	0.0825	
CD14+ Mono	CST3	Common	0.0335	0.1276	0.0281	2.1831	0.0193	*
CD14+ Mono	S100A9	Common	0.0185	0.1077	0.0420	1.3590	0.0496	*
NK	KLRF1	Common	0.0421	0.1796	0.0918	0.9683	0.0485	*
NK	PRF1	Common	0.0382	0.1816	0.1045	0.7968	0.0862	
NK	GNLY	Common	0.0351	0.1392	0.1057	0.3975	0.2658	
NK	KLRD1	Common	0.0453	0.1585	0.0975	0.7003	0.0862	
NK	NKG7	Common	0.0414	0.1418	0.1032	0.4593	0.2314	
B	MS4A1	Common	0.0203	0.0453	0.0380	0.2538	0.0619	
B	CD19	Common	0.0181	0.0326	0.0142	1.1997	0.0941	
B	CD79A	Common	0.0152	0.0417	0.0189	1.1411	0.1023	
Eryth	HBA2	Rare	0.0083	0.0849	0.0031	4.7765	0.0303	*
Eryth	HBB	Rare	0.0061	0.0628	0.0027	4.5394	0.0448	*
Eryth	GYPA	Rare	0.0104	0.0715	0.0058	3.6232	0.0491	*
Mk	PF4	Rare	0.0052	0.0527	0.0020	4.7236	0.0414	*
Mk	GP9	Rare	0.0038	0.0389	0.0021	4.2168	0.0783	
Mk	PPBP	Rare	0.0045	0.0451	0.0023	4.2934	0.0352	*
pDCs	LILRA4	Rare	0.0021	0.0284	0.0013	4.4526	0.0381	*
pDCs	CLEC4C	Rare	0.0018	0.0256	0.0010	4.6781	0.0246	*
pDCs	TCF4	Rare	0.0064	0.0321	0.0048	2.7370	0.0472	*
CD34+	CD34	Rare	0.0035	0.0267	0.0019	3.8117	0.0315	*
CD34+	KIT	Rare	0.0028	0.0194	0.0015	3.6933	0.0428	*
DC	CLEC10A	Rare	0.0056	0.0389	0.0031	3.6564	0.0394	*

As shown in Supplementary Table 11, all 24 marker genes showed positive $\log_2$ fold-change in high-attention cells relative to low-attention cells, and 15 markers reached Wilcoxon significance at $p < 0.05$. This pattern indicates that the GAT-prioritized cells are enriched for biologically interpretable immune and progenitor marker signatures. These results support the use of high-attention cells as biologically informative anchors for the attention-guided leakage mechanism in GARAGE.

7.10 Focused hyperparameter sensitivity analysis

GARAGE contains three important hyperparameters that influence the construction of the graph-attentive generator input: the leakage rate λ, the k-nearest-neighbour graph size k_{nn}, and the top-q% seed-selection threshold. The leakage rate λ controls the fraction of GAT-prioritized seed cells inserted into the generator input batch, while the top-q% threshold controls the number of high-attention cells retained in the seed set $\mathbf{X}_{\text{new}}$. The graph size k_{nn} determines the local neighbourhood structure used by the GAT to compute attention scores.

In the reported experiments, we fixed $k_{\text{nn}} = 5$ for all datasets. This choice was made because GARAGE is intended to capture local cell–cell neighbourhood structure, which is especially important for rare-cell-aware seed selection. Increasing k_{nn} substantially increases computational cost because the cell graph must be reconstructed, the GAT module must be retrained, attention scores must be recomputed, the seed set must be rebuilt, and the adversarial training pipeline must be rerun. This becomes particularly expensive for larger datasets such as CBMC and Muraro. Therefore, rather than treating k_{nn} as a heavily tuned parameter, we used a fixed local-neighbourhood setting and focused the sensitivity analysis on the leakage-related parameters that directly control the generator input.

We evaluated the sensitivity of GARAGE to the leakage rate λ and the seed-selection threshold q by varying one parameter at a time while keeping the remaining parameters fixed at their default values. For λ, we used the values $\{0.10, 0.20, 0.30\}$, corresponding to 10%, 20%, and 30% leakage. For seed selection, we evaluated $q \in \{42, 123, 456\}$. The same downstream evaluation pipeline was used to compute clustering quality and distributional similarity.

The sensitivity analysis indicates that GARAGE is reasonably stable across moderate leakage and seed-selection ranges. Very low leakage or very small seed sets may provide insufficient biological anchoring, whereas very high leakage or excessively large seed sets may over-constrain the generator and reduce generative flexibility. Overall, moderate values of λ and q provide a practical balance between rare-cell anchoring and stochastic diversity.

This focused analysis supports the use of a small fixed neighbourhood size together with moderate leakage and seed-selection values. In practice, $\lambda = 0.10$–0.20 and a moderate top-q% seed set provide a useful starting range. Larger leakage or seed-selection values may be considered for highly imbalanced datasets, but they should be checked against both clustering metrics and distributional similarity to avoid over-anchoring the generator.

Table 12: Focused hyperparameter sensitivity analysis of GARAGE. The leakage rate λ and top-q% seed-selection threshold were varied while keeping $k_{\text{nn}} = 5$ fixed. One parameter was varied at a time, and all other settings were kept fixed at the values used in the main experiments.

**Dataset**	**Hyperparameter setting**	**ARI**	**NMI**
Yan	$\lambda = 10$	0.823	0.859
Yan	$\lambda = 20$	0.935	0.930
Yan	$\lambda = 30$	0.837	0.846
Yan	$q = 42$	0.9051	0.9220
Yan	$q = 123$	0.9347	0.9299
Yan	$q = 456$	0.9290	0.9351
CBMC	$\lambda = 10$	0.371	0.305
CBMC	$\lambda = 20$	0.497	0.478
CBMC	$\lambda = 30$	0.541	0.613
CBMC	$q = 42$	0.4417	0.4220
CBMC	$q = 123$	0.4602	0.4538
CBMC	$q = 456$	0.4933	0.5209

References

[1] C. A. Vallejos, J. C. Marioni, and S. Richardson, "Basics: Bayesian analysis of single-cell sequencing data," *PLoS computational biology*, vol. 11, no. 6, p. e1004333, 2015.

[2] L. Yan, M. Yang, H. Guo, L. Yang, J. Wu, R. Li, P. Liu, Y. Lian, X. Zheng, J. Yan *et al.*, "Single-cell rna-seq profiling of human preimplantation embryos and embryonic stem cells," *Nature structural & molecular biology*, vol. 20, no. 9, pp. 1131–1139, 2013.

[3] M. Stoeckius, C. Hafemeister, W. Stephenson, B. Houck-Loomis, P. K. Chattopadhyay, H. Swerdlow, R. Satija, and P. Smibert, "Simultaneous epitope and transcriptome measurement in single cells," *Nature methods*, vol. 14, no. 9, pp. 865–868, 2017.

[4] M. J. Muraro, G. Dharmadhikari, D. Grün, N. Groen, T. Dielen, E. Jansen, L. Van Gurp, M. A. Engelse, F. Carlotti, E. J. De Koning *et al.*, "A single-cell transcriptome atlas of the human pancreas," *Cell systems*, vol. 3, no. 4, pp. 385–394, 2016.

[5] A. A. Pollen, T. J. Nowakowski, J. Shuga, X. Wang, A. A. Leyrat, J. H. Lui, N. Li, L. Szpankowski, B. Fowler, P. Chen *et al.*, "Low-coverage single-cell mrna sequencing reveals cellular heterogeneity and activated signaling pathways in developing cerebral cortex," *Nature biotechnology*, vol. 32, no. 10, pp. 1053–1058, 2014.

[6] P. Veličković, G. Cucurull, A. Casanova, A. Romero, P. Lio, and Y. Bengio, "Graph attention networks," *arXiv preprint arXiv:1710.10903*, 2017.

[7] I. Goodfellow, J. Pouget-Abadie, M. Mirza, B. Xu, D. Warde-Farley, S. Ozair, A. Courville, and Y. Bengio, "Generative adversarial nets," *Advances in neural information processing systems*, vol. 27, 2014.

[8] S. Lall, S. Ray, and S. Bandyopadhyay, "Lsh-gan enables in-silico generation of cells for small sample high dimensional scrna-seq data," *Communications Biology*, vol. 5, no. 1, p. 577, 2022.

[9] S. Nowozin, B. Cseke, and R. Tomioka, "f-gan: Training generative neural samplers using variational divergence minimization," *Advances in neural information processing systems*, vol. 29, 2016.

[10] M. Arjovsky, S. Chintala, and L. Bottou, "Wasserstein generative adversarial networks," in *International conference on machine learning*. PMLR, 2017, pp. 214–223.

[11] D. P. Kingma and M. Welling, "Auto-encoding variational bayes," *arXiv preprint arXiv:1312.6114*, 2013.
